# Comparison of plasma- and saliva-derived exosomal miRNA profiles reveals diagnostic potential in head and neck cancer

**DOI:** 10.3389/fcell.2022.971596

**Published:** 2022-08-22

**Authors:** Linda Hofmann, Tsima Abou Kors, Jasmin Ezić, Beate Niesler, Ralph Röth, Sonja Ludwig, Simon Laban, Patrick J. Schuler, Thomas K. Hoffmann, Cornelia Brunner, Valentin Medyany, Marie-Nicole Theodoraki

**Affiliations:** ^1^ Department of Otorhinolaryngology, Head and Neck Surgery, Ulm University Medical Center, Ulm, Germany; ^2^ nCounter Core Facility, Institute of Human Genetics, University of Heidelberg, Heidelberg, Germany; ^3^ Department of Otorhinolaryngology, Head and Neck Surgery, University Hospital Mannheim, Medical Faculty Mannheim, University of Heidelberg, Mannheim, Germany

**Keywords:** exosomes, miRNA, HNSCC, liquid biopsy, plasma, saliva, HPV

## Abstract

**Background:** Head and neck squamous cell carcinomas (HNSCC) lack tumor-specific biomarkers. Exosomes from HNSCC patients carry immunomodulatory molecules, and correlate with clinical parameters. We compared miRNA profiles of plasma- and saliva-derived exosomes to reveal liquid biomarker candidates for HNSCC.

**Methods:** Exosomes were isolated by differential ultracentrifugation from corresponding plasma and saliva samples from 11 HNSCC patients and five healthy donors (HD). Exosomal miRNA profiles, as determined by nCounter^®^ SPRINT technology, were analyzed regarding their diagnostic and prognostic potential, correlated to clinical data and integrated into network analysis.

**Results:** 119 miRNAs overlapped between plasma- and saliva-derived exosomes of HNSCC patients, from which 29 tumor-exclusive miRNAs, associated with *TP53*, *TGFB1, PRDM1, FOX O 1* and *CDH1* signaling, were selected. By intra-correlation of tumor-exclusive miRNAs from plasma and saliva, top 10 miRNA candidates with the strongest correlation emerged as diagnostic panels to discriminate cancer and healthy as well as potentially prognostic panels for disease-free survival (DFS). Further, exosomal miRNAs were differentially represented in human papillomavirus (HPV) positive and negative as well as low and high stage disease.

**Conclusion:** A plasma- and a saliva-derived panel of tumor-exclusive exosomal miRNAs hold great potential as liquid biopsy for discrimination between cancer and healthy as well as HPV status and disease stage. Exosomal miRNAs from both biofluids represent a promising tool for future biomarker studies, emphasizing the possibility to substitute plasma by less-invasive saliva collection.

## 1 Background

The tumor microenvironment of head and neck squamous cell carcinoma (HNSCC) is highly immunosuppressive, with exosomes being prominent modulators of immunomodulation (Hofmann et al., 2020a). HNSCC management is hampered by the absence of specific tumor markers, and most patients present with locally advanced disease, lymph node or distant metastasis, or they experience loco-regional recurrence (Johnson et al., 2020). Therefore, biomarkers which enable early detection, diagnosis and therapy monitoring of HNSCC are highly desirable.

With a size of 30–150 nm, exosomes are the smallest of extracellular vesicles (EVs). They undergo a complex biogenesis in the endosomal compartment before they are being released into the circulation, where they mediate intercellular communication by transferring their cargo to recipient cells (Milane et al., 2015; Abels and Breakefield, 2016). Besides the surface and intra-exosomal protein cargo, the transfer of DNA (Elzanowska et al., 2021), mRNA and miRNA (Valadi et al., 2007) contributes to immunomodulation. Especially miRNAs, small, non-coding RNAs involved in a variety of cellular processes, are highly abundant in the exosomal cargo (Gallo et al., 2012). Dependent on their complementarity towards the target mRNA, they induce translational repression or degradation (Gebert and MacRae, 2019). Dysregulation of miRNA signaling is involved in the tumorigenesis of many cancers, including HNSCC. miRNAs are effectively and selectively packed into exosomes (Valadi et al., 2007; Groot and Lee, 2020), and their specific genetic profile as biomarkers fosters the potential of an easily accessible liquid biopsy.

Plasma of HNSCC patients is enriched with exosomes (Ludwig et al., 2017). They carry immunomodulatory molecules and can alter immune cell functions (Ludwig et al., 2017; Theodoraki et al., 2018a; Theodoraki et al., 2018b; Hofmann et al., 2020b). Our previous studies emphasized the potential of exosomes isolated from HNSCC patient’s plasma as liquid biomarkers for disease stage, tumor activity and progression (Theodoraki et al., 2018a; Theodoraki et al., 2018b; Theodoraki et al., 2018c; Hofmann et al., 2020b; Theodoraki et al., 2020). While these studies were based on protein and functional level, other studies highlighted the impact of exosomal miRNAs as valuable biomarkers for progression of oral, laryngeal, and esophageal cancer (Tanaka et al., 2013; Zhao et al., 2020; He et al., 2021).

Not only plasma- but also saliva-derived exosomes represent rich sources of miRNAs (Michael et al., 2010; Gallo et al., 2012). Unlike plasma, standardized salivary diagnostic is not implemented in the clinic routine. Access to saliva samples is limited and collection requires patient’s collaboration for high quality sample acquisition. By now, exosome analysis from saliva is not as frequent as plasma although collection is even less invasive and atraumatic for patients. Tumor exosomes can either be directly released into saliva by malignant cells or reach salivary glands via circulation (Nonaka and Wong, 2017). Saliva-derived exosomes were shown to have disease-indicating biomarker potential in lung (Sun et al., 2017), pancreatic (Katsiougiannis et al., 2017) and breast cancer (Lau and Wong, 2012). Even more, promising data are available using exosomal miRNAs from saliva for differentiation of patients with oral cancer from healthy subjects (Langevin et al., 2017; Gai et al., 2018; He et al., 2020).

To explore the combinatorial potential of exosomes derived from both plasma and saliva, this study systematically compared the exosomal miRNA profiles between plasma and saliva of HNSCC patients and healthy subjects. It aims to outline similarities and differences between the biofluids and evaluate the overall utility of exosomes carrying a specific genetic profile as easily accessible biomarkers for HNSCC.

## 2 Methods

### 2.1 Research subjects

Corresponding peripheral blood and saliva samples were obtained from 11 treatment-naive HNSCC patients with histologically confirmed tumors who were treated at the Department of Otorhinolaryngology, Head and Neck Surgery, Ulm University in 2020 and 2021. Additionally, corresponding blood and saliva samples from 5 age- and sex-matched HD were obtained. Blood and saliva collection were approved by the Ethics Committee of the University of Ulm (#90/15) and each patient provided informed consent.

### 2.2 Blood and saliva collection

Blood samples were collected in citrate tubes and centrifuged at 1,000 g for 10 min followed by 2,500 g for 10 min. Plasma aliquots were stored at −20°C.

For saliva collection between 8 and 12 a.m., patients were asked not to eat, drink or perform dental hygiene for at least 1 h before collection. Saliva was collected using Salivette^®^ plain cotton swabs (51.1534, Sarstedt, Nümbrecht, Germany) and kept on ice during the collection procedure. Swabs were centrifuged at 1,000 g for 2 min and saliva aliquots were stored at −80°C.

Supplementary Table S1 provides the clinicopathological characteristics of patients included in this study.

### 2.3 Exosome isolation by differential ultracentrifugation

Freshly thawed plasma (5 ml) or saliva (1 ml) was centrifuged at 2,000 g for 10 min and 12,000 g for 30 min at 4°C to remove cell debris and larger vesicles, followed by filtration through 0.22 μm syringe-filters (SLGPO33RB, Millipore, Burlington, MA, United States). The filter was washed with 5 ml (plasma) or 1 ml (saliva) PBS (yielding 1:1 dilution to reduce viscosity). Diluted plasma was ultracentrifuged at 110,000 g for 2 h at 4°C. The resulting exosome pellet was resuspended in 1 ml of PBS and again ultracentrifuged at 110,000 g for 70 min at 4°C. The washing step was repeated. Diluted saliva was ultracentrifuged at 120,000 g for 3 h at 4°C. Resulting exosome pellets were dissolved in 700 µl Qiazol, thoroughly vortexed and stored at −80°C.

### 2.4 Exosome characterization

The methods for exosome characterization from both biofluids were performed according to the minimal information for studies of extracellular vesicles (MISEV) 2018 guidelines (Théry et al., 2018) and are routinely performed as described in detail in our previous publications (Ludwig et al., 2017; Hofmann et al., 2020b) (EV-TRACK ID: EV210344).

### 2.5 miRNA profiling of exosomes

Total exosomal RNA was isolated using the miRNeasy Micro Kit (217084, Qiagen, Hilden, Germany) according to manufacturer’s instructions. Quality of exosomal RNA was assessed by Agilent 2100 Bioanalyzer (Agilent Technologies, Santa Clara, CA, United States) using a Pico Chip. Amount of exosomal RNA was determined using Qubit Fluorometer (Thermo Fisher Scientific, Waltham, MA, United States). Exosomal RNA was stored at −80°C.

miRNA profiling was performed using the nCounter^®^ SPRINT system (Nanostring Technologies, Seattle, WA, United States) at the nCounter^®^ Core Facility of the University of Heidelberg, Germany. Total exosomal RNA was applied to the Human v3 miRNA Assay comprising the measurement of 827 human miRNAs. The assay was performed with 500 pg of total exosomal RNA following the manufacturer’s instructions.

### 2.6 Computational and statistical analysis

For simplicity, “hsa” has been removed from the miRNA names throughout the manuscript. miRNA expression data were analyzed using the nSolver 4.0 software. Raw data are counts of individual miRNAs present in the exosome sample and were normalized using the positive ligation controls.

Venn diagrams were generated using InteractiVenn (http://www.interactivenn.net/) (Heberle et al., 2015).

**FIGURE 1:**
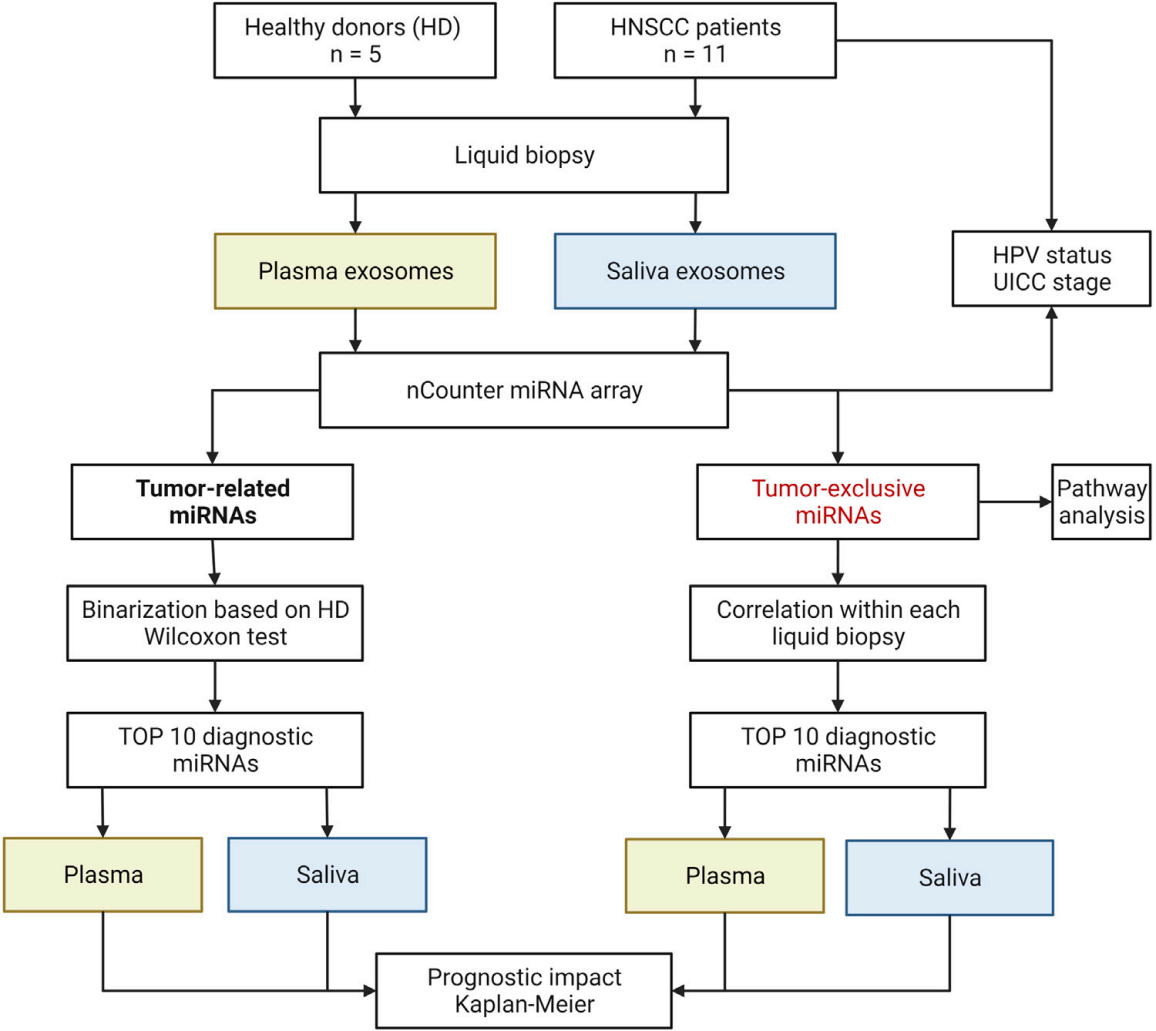
Schematic study workflow. HD, healthy donor: HPV, human papilloma virus: UICC, Union for International Cancer Control. Created with BioRender.com.

Figure 1 shows the overall analysis workflow of the study. Data analysis was performed in R (4.1.1, RRID:SCR_001905). Tumor-related miRNA counts were binarized to healthy donors (HD), matrixStats package (0.61.0), using a cut off calculated as Q3_HD_ + SD_HD_. Differential expression for tumor-exclusive miRNAs between plasma and saliva was calculated using paired Wilcoxon signed-rank test; differential expression for tumor-upregulated miRNAs between HNSCC patients and HD was calculated using unpaired Wilcoxon signed-rank test, ggpubr package (0.4.0). miRNA correlation of tumor-exclusive miRNAs was calculated, Hmisc package (4.6-0), using Pearson’s correlation. Correlation matrices were ordered using hierarchical clustering, corrplot package (0.92). N-integration of the tumor-exclusive plasma and saliva top 10 panels on individual patient level and production of the intercorrelation matrix were performed using mixOmics package (6.19.4). Waterfall and violin plots were generated using ggplot2 package (3.3.5).

Pathway and network analysis were performed using Ingenuity Pathway Analysis (IPA, Qiagen).

Volcano and box plots were generated in GraphPad Prism (version 9, GraphPad Software, San Diego, CA, United States, RRID:SCR_002798). Volcano plots were using the log2 expression ratio of each miRNA and the negative log10 of the *p*-value. In box-and-whisker blots, the line represents the median, the box shows the interquartile range (25–75%) and the whiskers indicate the range. Comparison of HPV status and UICC stage were analyzed by Mann-Whitney test for independent samples and *p* ≤ 0.05 was considered statistically significant.

Survival analysis was conducted by fitting survival curves using the survival package (3.3-1) and generating Kaplan-Meier plots utilizing the survminer package (0.4.9). Patients were binarized according to their expression of miRNAs from the panels. For Top eight tumor-upregulated panel from plasma, miRNAs were binarized to HD, as described above, and patients were grouped to “high” when >4 miRNAs from the panel were expressed. For Top 10 tumor-exclusive panel from plasma and saliva, the median was applied to the miRNAs as cut-off to determine “normal” and “high,” and patients were considered “high” when >5 miRNAs (plasma) or >1 miRNA (saliva) from the panel were expressed above the median.

## 3 Results

### 3.1 Study population

The clinicopathological characteristics of the HNSCC patients (*n* = 11) are listed in Supplementary Table S1. At time-point of diagnosis, the mean age was 59 years with a range from 49 to 64 years. The majority of patients (91%) was male. The primary tumor was located in the oral cavity (9%), pharynx (73%) or larynx (18%). 45% of patients presented with an advanced tumor stage (T3/4) and 73% had lymph node metastasis. According to the eighth edition of Union for International Cancer Control (UICC), 45% percent of the patients were UICC stage I or II, and 55% were UICC stage III or IV. The human papillomavirus (HPV) status, routinely determined by p16 immunohistochemistry and PCR for HPV-DNA, was positive in four patients, negative in six patients, and not evaluated in one patient. Two patients developed a recurrence, while nine patients remained disease-free during the follow-up of 14 months (mean).

### 3.2 Characterization of plasma- and saliva-derived exosomes and exosomal RNA

**FIGURE 2:**
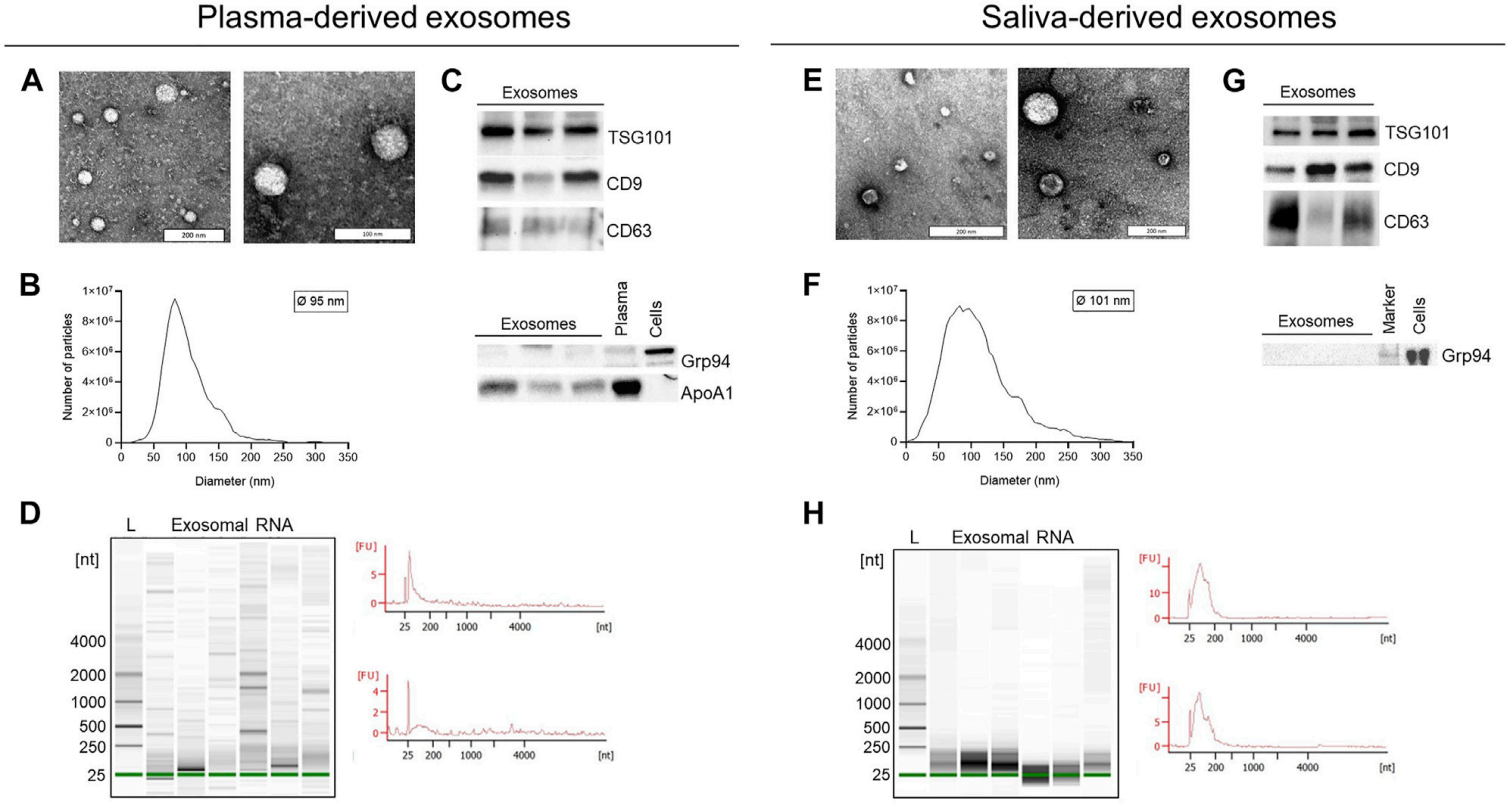
Characterization of exosomes and exosomal RNA isolated from plasma and saliva. **(A,E)** Representative transmission electron microscopy images of exosomes isolated from plasma **(A)** or saliva **(E)** by ultracentrifugation. **(B,F)** Representative size distribution histogram and mean diameter of plasma- **(B)** and saliva- **(F)** derived exosomes analyzed by nanoparticle tracking analysis (NTA). **(C,G)** Western blot analysis of exosomes isolated from plasma **(C)** or saliva **(G)**. Exosomal markers TSG101, CD9, and CD63 were present and the negative marker Grp94 was absent in all preparations. Exosomes from plasma carry low amounts of ApoA1. Plasma and cell lysates were used as positive control for ApoA1 and Grp94. **(D,H)** Representative Agilent bioanalyzer gel images and electropherograms of total exosomal RNA isolated from plasma **(D)** or saliva **(H)** and analyzed with the Pico Chip. L, ladder/size marker: Nt, nucleotides: FU, arbitrary fluorescent unit.

Exosomes isolated from plasma and saliva were evaluated for morphology by transmission electron microscopy (TEM), for size by nanoparticle tracking analysis (NTA) and for their protein composition by Western blot (Figure 2). Both plasma- and saliva-derived exosomes showed vesicular morphology (Figures 2A,E) and were within the normal range of exosome size with a median diameter of 95 and 101 nm, respectively (Figures 2B,F). Furthermore, exosome preparations from plasma and saliva contained the endosomal marker TSG101 and vesicle-associated proteins CD9 and CD63 (Figures 2C,G). The cellular marker Grp94 was not detected and the amount of ApoA1 was lower in plasma-derived exosomes compared to pure plasma. Agilent bioanalyzer analysis identified the presence of small RNAs (< 250 nucleotides) and the absence of cell-derived 18S and 28S RNA in both exosomal RNA from plasma and saliva (Figures 2D,H). The density of small RNAs was higher in saliva- compared to plasma-derived exosomes.

### 3.3 Identification of tumor-related and tumor-exclusive miRNAs from plasma and saliva of HNSCC patients and their involvement in tumorigenesis

**FIGURE 3:**
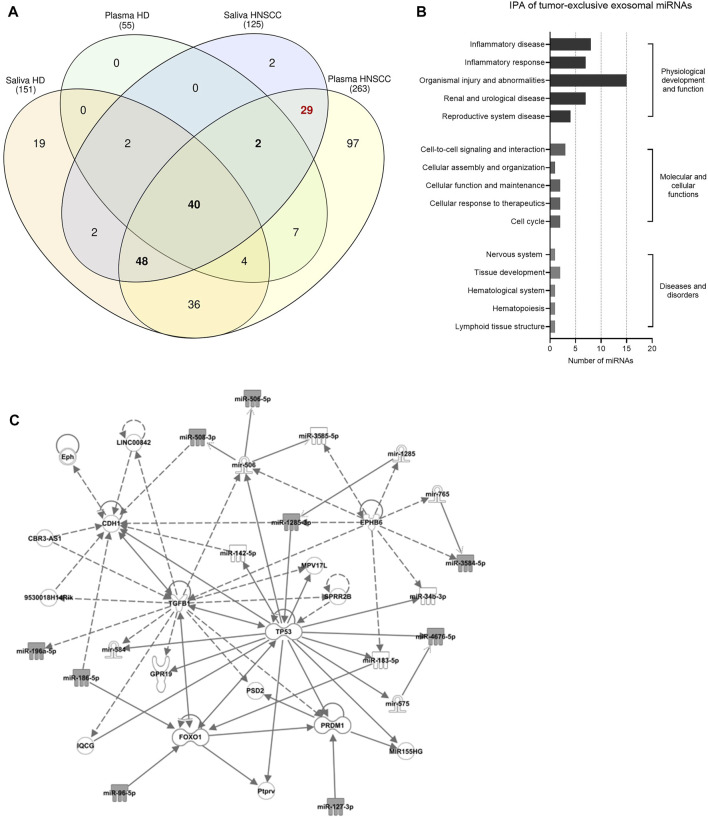
Identification of tumor-related and tumor-exclusive miRNAs from plasma and saliva of HNSCC patients and their involvement in tumorigenesis. **(A)** Venn diagram of exosomal miRNAs detected in plasma and saliva of healthy donors (HD, *n* = 5) and HNSCC patients (*n* = 11) to identify tumor-related (i.e., overlap between HNSCC plasma and HNSCC saliva and presence in HD plasma and/or HD saliva) and tumor-*exclusive* exosomal miRNAs (i.e., overlap between HNSCC plasma and HNSCC saliva while absent in both HD biofluids). 90 tumor-related exosomal miRNAs are shown in bold and 29 tumor-exclusive exosomal miRNAs are marked in red. **(B,C)** Ingenuity Pathway Analysis (IPA) of tumor-exclusive exosomal miRNAs from plasma and saliva of HNSCC patients (*n* = 11). **(B)** Shows diseases and biological functions most significantly associated with the identified tumor-exclusive miRNAs. Categories within a group are shown in increasing order of *p*-value. **(C)** Shows the involved cancer related network. Grey-colored symbols represent miRNAs identified in this study. Solid lines indicate direct interaction, dashed lines indicate indirect relation.

To evaluate the potential of the biofluids as starting material for exosome-based liquid biopsy in HNSCC, systematic comparison between plasma- and saliva-derived exosomes was performed. In plasma-derived exosomes of HNSCC patients, more than twice the number of miRNAs was detected compared to saliva-derived exosomes of HNSCC patients (263 vs. 125, Figure 3A). Further, 119 exosomal miRNAs overlapped between plasma- and saliva-derived exosomes of HNSCC patients (Supplementary Table S2), corresponding to 45% of total miRNAs detected in plasma and 95% of total miRNAs detected in saliva of HNSCC patients.

Upon consideration of exosomal miRNAs present in plasma- and saliva-derived exosomes of HNSCC patients and HD, tumor-related and tumor-exclusive exosomal miRNAs were filtered out by applying two strategies with different stringency. Exosomal miRNAs, which overlapped between HNSCC plasma and HNSCC saliva *and* were additionally present in HD plasma and/or HD saliva, were considered tumor-related (presented in bold in Figure 3A). Exosomal miRNAs, which overlapped between HNSCC plasma and HNSCC saliva and were not present in both HD plasma and HD saliva, were considered tumor-exclusive (marked in red in Figure 3A). Totally, 90 tumor-related and 29 tumor-exclusive miRNAs were identified and are listed in Supplementary Table S2.

Looking at the normalized counts of the tumor-exclusive exosomal miRNAs (Supplementary Figure S1), the majority (20 out of 29) did not show significant differences in the expression levels between plasma and saliva, indicating representation of tumor-exclusive miRNAs in plasma and saliva at comparable levels. Pathway and network analysis revealed a biological role of tumor-exclusive exosomal miRNAs in “inflammatory disease and response” and “organismal injury and abnormalities” (Figure 3B). Their molecular functions were related to cell-to-cell signaling/interaction and cellular assembly, organization, function and maintenance as well as cellular response to therapeutics and cell cycle. Core hubs of the associated network were *TP53*, *TGFB1*, *PRDM1*, *FOX O 1* and *CDH1* (Figure 3C), indicating their involvement in pathways promoting tumorigenesis.

### 3.4 Tumor-upregulated miRNAs are potent diagnostic biomarkers only in plasma-derived exosomes

**FIGURE 4:**
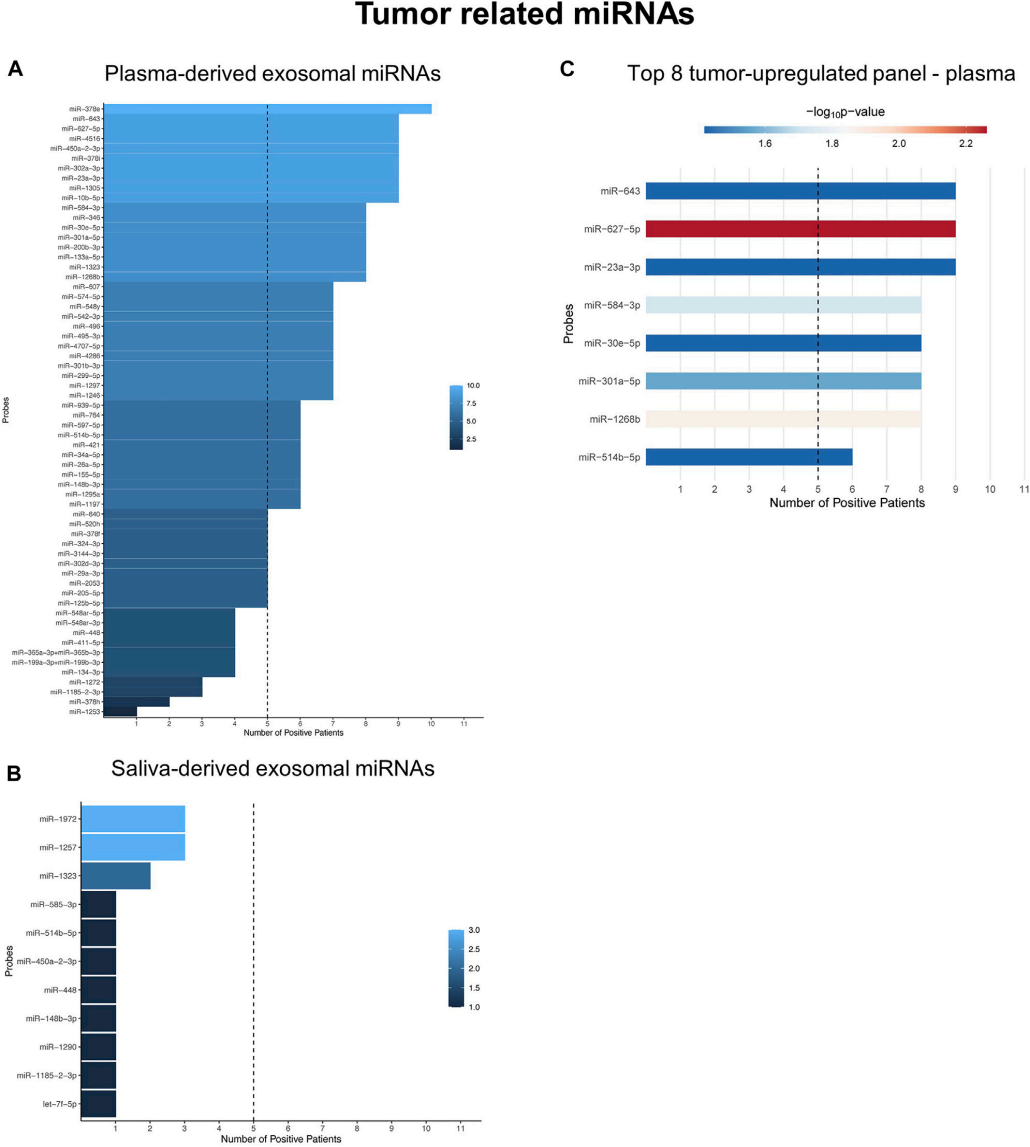
Tumor-related miRNAs are potent diagnostic biomarkers only in plasma-derived exosomes. **(A,B)** Waterfall plots of binarized tumor-related exosomal miRNAs from plasma **(A)** and saliva **(B)** showing the number of HNSCC patients with positive expression. miRNAs exceeding the threshold of 50% (dashed line) were considered tumor-*upregulated*. **(C)** Shows the Top eight tumor-upregulated exosomal miRNAs from plasma with discriminatory potential between HNSCC patients and HD, based on unpaired Wilcoxon test.

Binarization of tumor-related miRNAs in plasma- and saliva-derived exosomes identified tumor-upregulated miRNAs (Supplementary Figure S2; Supplementary Tables S3, S4). The abundance of tumor-upregulated miRNAs showed great differences between plasma and saliva: While 41 plasma-derived exosomal miRNAs were present in at least 50% of investigated HNSCC patients (Figure 4A), none of the saliva-derived exosomal miRNAs reached this cut-off (Figure 4B). Due to stable upregulation of plasma-derived exosomal miRNA candidates they appear more suitable as biomarkers compared to saliva-derived exosomal miRNAs distinguishing healthy from cancer patients. To propose a diagnostic panel consisting of the top tumor-related miRNA candidates, unpaired Wilcoxon test of plasma-derived exosomal miRNAs was performed. Eight miRNA candidates with significantly higher counts in HNSCC patients compared to HD (Figure 4C; Supplementary Figure S3) were identified as the top biomarkers associated with cancer vs. non-cancer: miR-627-5p (*p* = 0.005), miR-1268b (*p* = 0.013), miR-584-3p (*p* = 0.019), miR-301a-5p (*p* = 0.027), miR-643, miR-23a-3p, miR-30e-5p, and miR-514b-5p (all *p* = 0.038).

### 3.5 Tumor-exclusive exosomal miRNAs reveal diagnostic potential in both plasma and saliva

**FIGURE 5:**
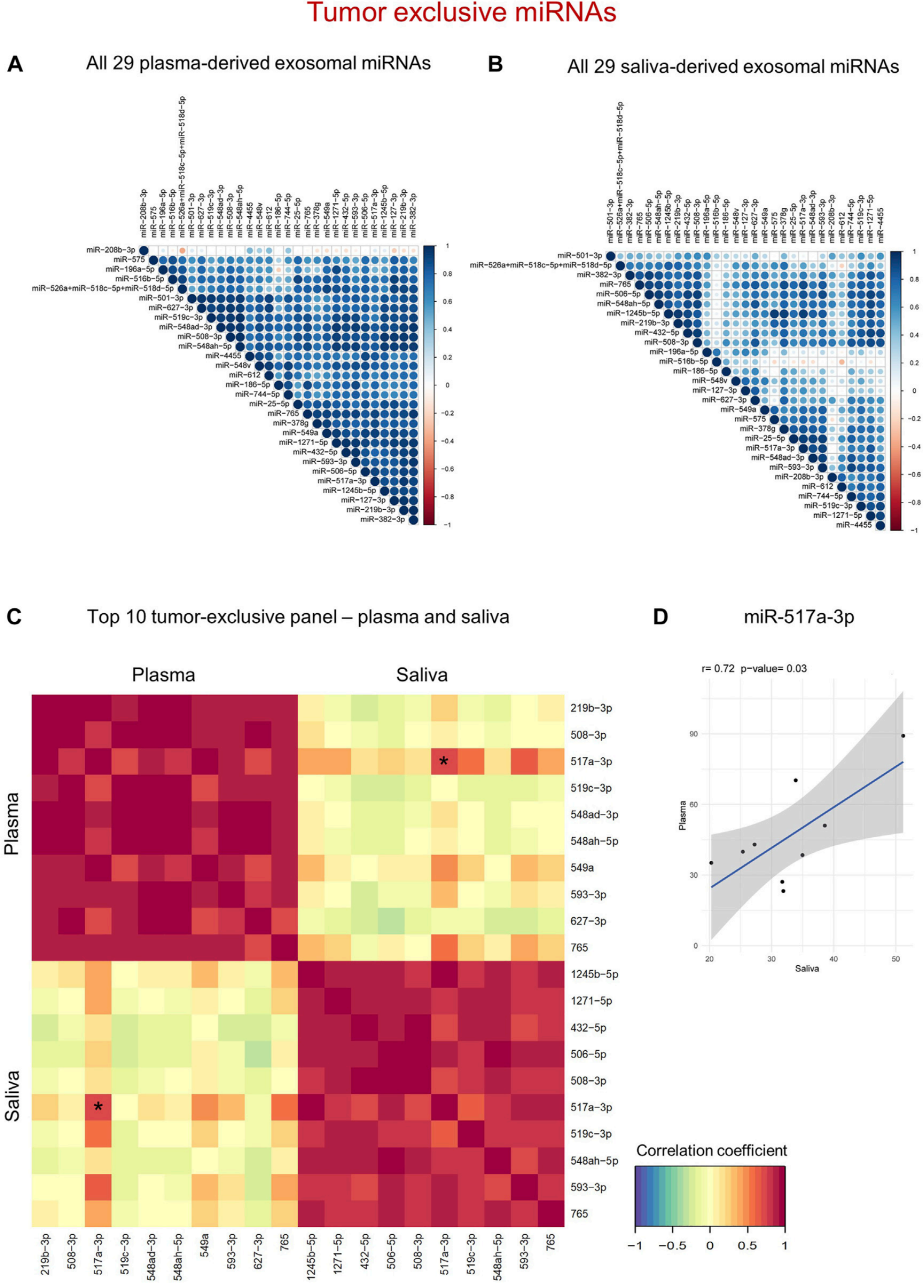
Tumor-exclusive exosomal miRNAs reveal diagnostic potential in both plasma and saliva. **(A,B)** Correlation matrix of tumor-exclusive exosomal miRNAs from plasma **(A)** and saliva **(B)**. The stronger the color (blue = positive, red = negative correlation) and the bigger the circle, the higher the correlation between individual miRNAs. **(C)** N-integration correlation matrix that reflects intra- and inter-correlation within and between the two diagnostic panels. Red = Positive correlation, yellow = no correlation, green = negative correlation. The asterisk marks the only miRNA with significant inter-correlation, which is further shown in **(D)**. **(D)** Scatter plot showing normalized counts of miR-517a-3p with significant positive correlation between plasma and saliva of individual HNSCC patients as determined by Pearson’s correlation analysis.

To identify clusters of co-expressed miRNAs in HNSCC patients, intra-correlation analysis of tumor-exclusive exosomal miRNAs was performed for plasma and saliva (Figures 5A,B; Supplementary Tables S5, S6). For both biofluids, clusters of co-expressed miRNAs were identified. The miRNAs detected in plasma formed one distinctively large and coherent cluster characterized by strong intercorrelations, except for miR-186-5p weakly correlating with miR-196a-5p and miR-575 (Figure 5A). Nevertheless, only one miRNA from plasma, miR-208b-3p, did not exhibit a strong correlation with the remaining miRNAs. To break down the clusters to a panel with top 10 diagnostic biomarkers from plasma, two miRNAs with the strongest correlation coefficient of the entire matrix were selected (miR-219b-3p and miR-506-3p), and their co-expressed miRNAs were intersected (Supplementary Figure S4). Of the overlapping miRNAs, eight candidates were picked based on the cut-off correlation coefficient ≥0.8, resulting in a diagnostic panel of 10 exosomal miRNAs showing the strongest co-expression (Figure 5C).

In saliva, five defined clusters with 9–19 miRNAs were detected (Figure 5B). To establish the strongest diagnostic panel of saliva-derived exosomal miRNAs from the detected clusters, candidate miRNAs were filtered by applying the cut-offs correlation coefficient ≥0.8 and *p* ≤ 0.01. Three leading miRNAs with greater ten co-expressed miRNAs were identified (miR-1245b-5p, miR-1271-5p, and miR-765). Upon intersection of their co-expressed miRNAs, seven miRNAs were overlapping between the three groups (Supplementary Figure S5), resulting in a diagnostic panel of 10 exosomal miRNAs from saliva showing the strongest co-expression (Figure 5C).

While the proposed diagnostic panels from plasma and saliva, consisting of the top 10 biomarker candidates, correlated very well within one biofluid, inter-correlation between the two panels was distorted (Figure 5C; Supplementary Table S7). Only one miRNA, miR-517a-3p, displayed a significantly positive correlation (*r* = 0.72) between plasma and saliva of HNSCC patients (Figure 5D). Therefore, compared to tumor-related miRNAs, tumor-exclusive miRNAs from both biofluids are powerful diagnostic tools distinguishing cancer from non-cancer, albeit by the usage of separate panels.

### 3.6 Prognostic potential of the proposed diagnostic exosome-based panels from plasma and saliva

**FIGURE 6:**
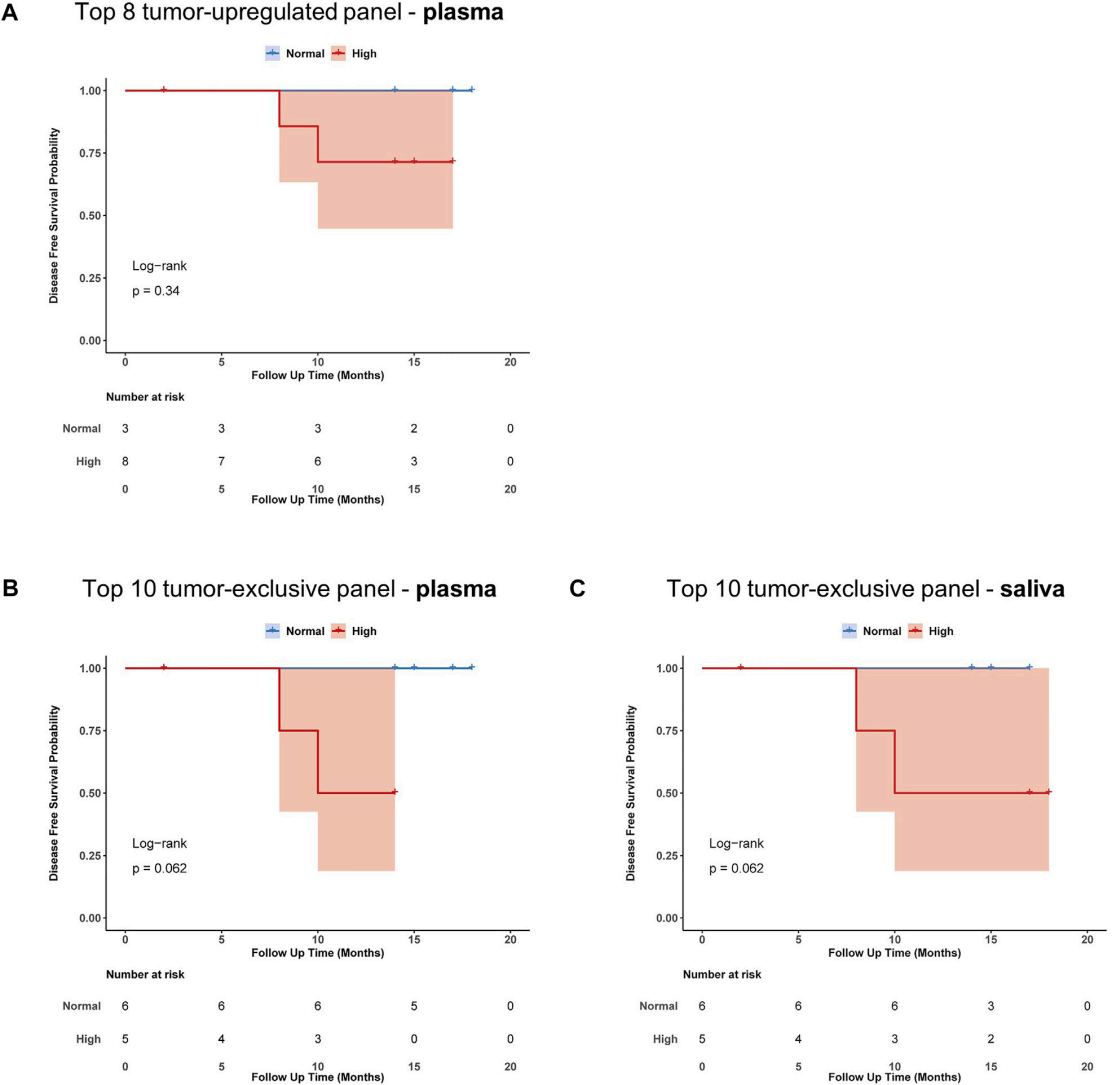
Prognostic impact of proposed exosome-based miRNA panels from plasma and saliva. Kaplan-Meier plots with log-rank test for disease-free survival (DFS) of HNSCC-patients binarized according to their expression of miRNAs present in **(A)** tumor-upregulated panel from plasma **(B)** tumor-exclusive panel from plasma and **(C)** tumor-exclusive panel from saliva. Blue indicates normal expression, red indicates high expression, shaded area indicates the confidence interval.

To evaluate the impact of exosomal miRNA profiles on clinical outcome of HNSCC patients, Kaplan-Meier survival with log-rank analysis was performed with the proposed tumor-related and tumor-exclusive miRNA panels (Figure 6). Although non-significant, all three panels showed a trend towards worse disease-free survival (DFS) in patients with high expression of the miRNAs included in the panel. When looking at single miRNAs, the strongest prognostic candidate showing significant difference in DFS was miR-519c-3p (*p* = 0.017, Supplementary Figure S6) from saliva-derived exosomes.

### 3.7 miRNA profiles of plasma- and saliva-derived exosomes of HPV^+^ and HPV^−^ HNSCC patients

**FIGURE 7:**
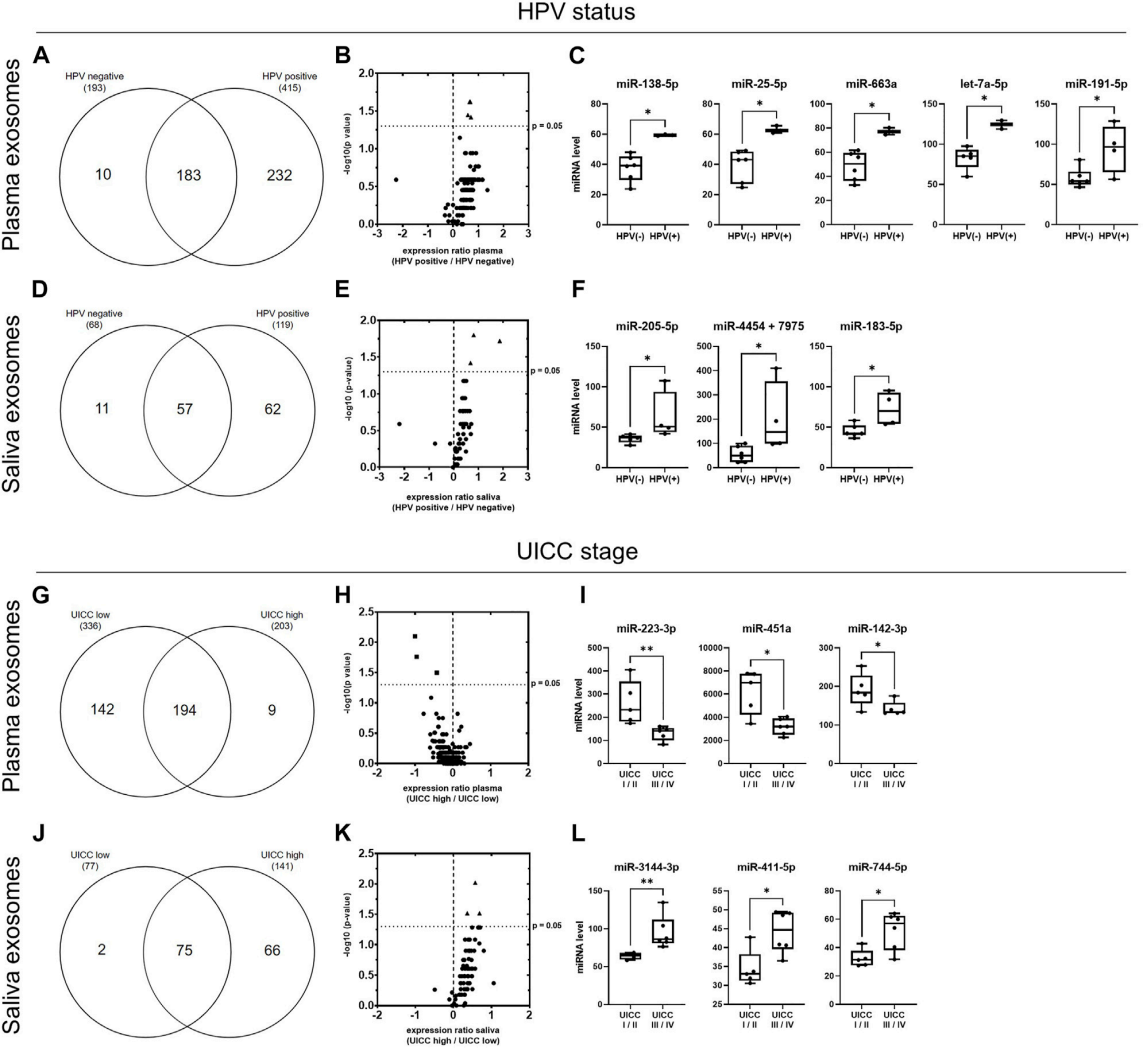
miRNA profiles of plasma- and saliva-derived exosomes dependent on HPV status and UICC stage. **(A,D)** Venn diagram of miRNAs detected in plasma- **(A)** and saliva- **(D)** derived exosomes of HPV^−^ (*n* = 6) and HPV^+^ (*n* = 4) HNSCC patients. **(B,E)** Volcano plots showing the log2 expression ratio of those miRNAs overlapping between patients with HPV^−^ and HPV^+^ disease. Each dot represents one miRNA. miRNAs at x > 0 are upregulated in exosomes from HPV^+^ patients, while miRNAs at x < 0 are downregulated in exosomes from HPV^+^ patients compared to patients with HPV^−^ disease. miRNAs shown as triangle are significantly upregulated (*p* ≤ 0.05, cut-off marked as y-axis intersection) in exosomes from HPV^+^ patients. These are shown in detail in **(C,F)**. Individual miRNA counts from *n* = 10 HNSCC patients are plotted as box-and-whisker blots representing the median value, the 25th and 75th quartiles and the range. * corresponds to *p* ≤ 0.05, as determined by Mann-Whitney test. **(G,J)** Venn diagram of miRNAs detected in plasma- **(G)** and saliva- **(J)** derived exosomes of HNSCC patient with low (n = 5) and high (n = 6) UICC stage. **(H,K)** Volcano plots showing the log2 expression ratio of those miRNAs overlapping between patients with low and high UICC stage. Each dot represents one miRNA. miRNAs at x > 0 are upregulated in exosomes from UICC high stage patients, while miRNAs at x < 0 are downregulated in exosomes from UICC high compared to low stage patients. miRNAs shown as square are significantly downregulated and miRNAs shown as triangle are significantly upregulated (*p* ≤ 0.05, cut-off marked as y-axis intersection) in exosomes from UICC high stage patients. These are shown in detail in **(I,L)**. Individual miRNA counts from *n* = 11 HNSCC patients are plotted as box-and-whisker blots representing the median value, the 25th and 75th quartiles and the range. * corresponds to *p* ≤ 0.05, as determined by Mann-Whitney test.

To highlight the HPV-dependent exosomal cargo in plasma or saliva, miRNA profiles were stratified and compared based on HPV status (Figures 7A–F). While 193 miRNAs were detected in plasma-derived exosomes of patients with HPV^−^ HNSCC, patients with HPV^+^ HNSCC carried more than twice as much miRNAs (415) (Figure 7A; Supplementary Table S8A). Ten exosomal miRNAs were exclusive for HPV^−^ disease, 232 for HPV^+^ disease and 183 overlapped between the two groups. The latter are further demonstrated in the Volcano plot (Figure 7B) with a majority (96%) showing higher levels in HPV^+^ cancers. Five exosomal miRNAs showed significantly higher counts in HPV^+^ compared to HPV^−^ patients (Figure 7C; Supplementary Table S10).

With 68 versus 119 miRNAs, saliva-derived exosomes of patients with HPV^−^ HNSCC carried less miRNAs compared to patients with HPV^+^ HNSCC (Figure 7D; Supplementary Table S8B). 11 exosomal miRNAs were exclusive for HPV^−^ disease, 62 for HPV^+^ disease and 57 overlapped between the two groups. The latter are further shown in the Volcano plot (Figure 7E), again with the majority (93%) showing higher levels in HPV^+^ cancers. For saliva-derived exosomes, three miRNAs showed significantly higher counts in patients with HPV^+^ compared to HPV^−^ disease (Figure 7F; Supplementary Table S10).

### 3.8 miRNA profiles of plasma- and saliva-derived exosomes of HNSCC patients with low and high UICC stage

Exosomes from plasma and saliva were analyzed regarding their ability to be contingent on UICC stage I/II (low) and stage III/IV (high) (Figure 7G–L). In plasma-derived exosomes, 336 miRNAs were detected in patients with low stage and 203 miRNAs in patients with high stage disease (Figure 7G; Supplementary Table S9A). 194 exosomal miRNAs were shared by patients with low and high UICC stage, while 142 were exclusive for low and nine for high UICC stage. Most of the overlapping miRNAs (77%) showed lower levels in patients with high UICC stage (Figure 7H). Three of these exosomal miRNAs had significantly lower counts in patients with high compared to low UICC stage (Figure 7I; Supplementary Table S10).

In saliva-derived exosomes, 77 miRNAs were detected in patients with low stage, while almost more than twice as many miRNAs (141) were present in patients with high stage disease (Figure 7J; Supplementary Table S9B). Only two miRNAs were exclusive for UICC low stage disease, 66 for UICC high stage disease and 75 were shared by the two groups. In contrast to plasma-derived exosomal miRNAs, the majority of the overlapping saliva-derived exosomal miRNAs (95%) showed higher levels in patients with high stage disease (Figure 7K). Three of these miRNAs had significantly elevated counts in patients with high compared to low UICC stage (Figure 7L; Supplementary Table S10).

### 3.9 Combinatorial potential of tumor-exclusive exosomal miRNAs from plasma and saliva

To evaluate if miRNAs from the proposed plasma or saliva panel can convey information regarding HPV status and UICC stage, an overview of the combinatorial potential of tumor-excusive exosomal miRNAs was created (Table 1). Only one miRNA from the plasma panel (miR-548ad-3p), and six miRNAs from the saliva panel provided additional information regarding HPV status. Simultaneous discrimination of UICC stage was only possible with 8 miRNAs from the saliva panel, but not with the plasma panel. The miRNAs included in the panels were further screened with the miRCancer database (Xie et al., 2013) to extract previously published associations with head and neck, oral, esophageal, laryngeal or nasopharyngeal cancer. Differential expression in tumor cell lines, tumor tissue, plasma/serum or saliva was previously described for 8 out of the 14 tumor-exclusive miRNAs but only miR-432-5p was formerly associated with exosomes.

**TABLE 1:** Combinatorial potential of tumor-exclusive exosomal miRNAs from plasma and saliva.

miRNA	Plasma			Saliva			Inter-correlation	Tumor entity
	Diagnostic panel	HPV status	UICC stage	Diagnostic panel	HPV status	UICC stage		
miR-1245b-5p				x	positive	high		
miR-1271-5p				x	positive	high		OSCC
miR-219b-3p	x							ESCC
miR-432-5p				x	positive	high		ESCC
miR-506-5p				x	positive	high		ESCC, OSCC, NPC
miR-508-3p	x			x	positive	high		
miR-517a-3p	x			x	positive	high	x	
miR-519c-3p	x			x		high		OSCC
miR-548ad-3p	x	positive						ESCC, OSCC
miR-548ah-5p	x			x				ESCC, OSCC
miR-549a	x							
miR-593-3p	x			x				
miR-627-3p	x							

ESCC/OSCC, Esophageal/oral squamous cell carcinoma: NPC, nasopharyngeal carcinoma: HPV, human papillomavirus: UICC, union for international cancer control.

## 4 Discussion

We evaluated if exosomal miRNAs from plasma and/or saliva can be used as diagnostic tools to distinguish HNSCC patients from HD. Overall, 119 miRNAs overlapped between exosomes derived from plasma and saliva of HNSCC patients, which corresponds to almost all miRNAs detected in saliva but only half of the miRNAs in plasma. One reason for this difference can be the broader origin of exosomal miRNAs in plasma compared to saliva. While plasma-derived exosomes present a mixture released by tumor, immune, endothelial and other (non-tumor) body cells, saliva-derived exosomes are mostly produced as filtrate of plasma and additionally released by surrounding/local epithelial cells.

Based on the study by Sun et al., comparing exosomal proteomics of saliva and serum for detection of lung cancer, the selection of tumor-related and tumor-exclusive miRNAs was performed. Binarization further narrowed tumor-related miRNAs to tumor-upregulated miRNAs, i.e., although they are found in healthy donors, they show higher levels in tumor patients thereby holding potential as biomarkers contributing to tumorigenesis and tumor progression. Becoming even more stringent, tumor-exclusive miRNAs are exclusively and simultaneously found in both biofluids of HNSCC patients, while not being present in healthy individuals at all. Compared to tumor-upregulated exosomal miRNAs, where plasma prevails over saliva with regard to biomarker potential, tumor-exclusive exosomal miRNAs hold great promise as diagnostic tools in both biofluids. Simultaneous analysis of co-expressed miRNAs from the detected clusters as panels (top 10) highly increases the power of a diagnostic tool, as the probability of detecting more than one of the correlated miRNAs is much higher compared to the examination of only a single miRNA. Thus, we propose separate tumor-exclusive, exosome-based miRNA panels from plasma and saliva, each consisting of the 10 miRNAs with the strongest intra-correlation for discrimination between HD and HNSCC patients. Both panels complement, yet not replace each other: Depending on the general condition of the patient (hemoglobin levels, veins, xerostomia), clinicians can choose which panel is more appropriate for individual patients, considering that plasma can be substituted by less-invasive saliva collection.

Tumor-exclusive exosomal miRNAs were found to be involved in pathways of tumorigenesis with *TP53*, *PRDM1*, *TGFB1* (encoding TGF-β1) and *CDH1* (encoding E-Cadherin) as core players. Together with *CDKN2A*, the tumor suppressor *TP53* belongs to the most frequently altered genes in HNSCC (Johnson et al., 2020). Somatic mutation of *TP53* takes place in a majority of HNSCC cancers (Lawrence et al., 2015; Pérez Sayáns et al., 2019) and chromosomal loss of *TP53* is associated with carcinoma *in situ* (Califano et al., 1996). Additionally, the transcriptional regulator *PRDM1* is dysregulated in a variety of malignancies (Casamassimi et al., 2020). The cytokine TGF-β1 promotes HNSCC tumorigenesis by pleiotropic mechanisms such as inhibition of T lymphocyte growth, recruitment of regulatory T cells and induction of epithelial to mesenchymal transition (Pang et al., 2018). *In vitro*, TGF-β1 induced down-regulation of the cell adhesion molecule E-Cadherin and promoted cell migration of HNSCC cells (Yu et al., 2011). Both the upregulation of TGF-β1 and the downregulation of E-Cadherin are hallmarks of epithelial to mesenchymal transition in HNSCC (Smith et al., 2013). Moreover, the *CDH1* gene has been reported to exhibit promotor methylation in 43% of HNSCC cancers (Steinmann et al., 2009). The involvement of exosomal miRNAs in these pathways identified by IPA highlights their functional role in HNSCC biogenesis, invasion and immune regulation.

Correlation of miRNA levels with clinical data (HPV status, UICC grade) was performed to identify miRNAs, which can be HPV-dependent or considered as biomarkers for disease stage. Although the gold standard for HPV differentiation is p16 immunohistochemistry and high-risk HPV testing of the tumor (HPV DNA), exosome-based HPV diagnostic offers faster results, the possibility of early information, even before taking a biopsy, and could also be applied to patients from whom biopsy cannot be obtained. Compared to HPV-negative patients, a higher number of exosomal miRNAs was detected in both biofluids of HPV-positive patients. This is in line with an *in vitro* study of Ludwig et al. (2020), showing a higher number of miRNA transcripts in exosomes isolated from a HPV-positive cell line in comparison to exosomes from a HPV-negative cell line. Even more, they found miR-205-5p to be exclusively present in HPV-positive exosomes and miR-1972 to be exclusively present in HPV-negative exosomes from *in vitro* conditions (Ludwig et al., 2020). In our patient cohort, these findings were partly confirmed: miR-205-5p was only found in plasma-derived exosomes of HPV-positive patients and miR-1972 only in plasma-derived exosomes of HPV-negative patients. In saliva-derived exosomes, miR-205-5p was found in both HPV-positive and HPV-negative patients but with significantly higher levels in HPV-positive patients, while miR-1972 was not detected in neither HPV-positive nor HPV-negative patients. Among the plasma-derived exosomal miRNAs exclusive for HPV-negative HNSCC patients, we further identified miR-496, which was previously found to be down-regulated by HPV type 16 in oropharyngeal cancer (Mason et al., 2018), and miR-517c-3p, which was upregulated in HPV-negative tonsillar tumors (Vojtechova et al., 2016). HPV-positive HNSCC are more immunogenic and have a more favorable outcome compared to HPV-negative disease (Ang et al., 2010). Based on *in silico* approach using miEAA (Kern et al., 2020), 88% and 86% of all HPV-specific plasma-derived exosomal miRNAs identified in our study were annotated to the KEGG terms “HPV infection” and “Viral carcinogenesis.” As such, HPV-specific exosomal miRNAs can contribute to HPV-induced tumorigenesis and changes of the immune phenotype by selective targeting of tumor and immune regulatory genes.

Finally, we addressed the combinatorial potential of the proposed diagnostic tumor-exclusive, exosome-based plasma and saliva panels regarding their ability to convey information on HPV status and UICC stage. Both panels provide information for discrimination between HNSCC and HD, albeit with different miRNA candidates. Six miRNAs can be found in both panels, providing redundant diagnostic information and being considered as putative universal HNSCC biomarkers. Regarding clinical parameters, the same miRNA from both biofluids did not provide redundant information. One possible explanation is that the presence and functionality of exosomal miRNAs depend on their origin and the environment in which they are expressed. Exosomal miRNAs from saliva provided better information regarding clinical parameters, which might be due to their proximity to the HNSCC site and the higher presence of tumor-exosomes compared to exosomes of other origin (e.g., immune cells) (Hofmann et al., 2022). This makes saliva an attractive source of eventually more specific tumor biomarkers, while changes in the immune system or the presence of lymph node metastasis might be better reflected in plasma- compared to saliva-derived exosomes. The exact role of many exosomal miRNAs in the context of HNSCC remains unclear, as most previously published data refer to tumor tissue, total plasma or saliva, while studies involving miRNAs in EVs are limited and often based on *in vitro* studies. Recently, miR-432-5p was evaluated as part of a serum exosomal miRNA model for pre-operative prediction of lymph node metastasis in ESCC patients (Liu et al., 2020). In our study, this tumor-exclusive miRNA was included into the diagnostic saliva panel and showed biomarker potential for early detection of cancer, disease stage and HPV status. Overall, the data and panels arising from our study advance the use of exosomal miRNAs as diagnostic liquid biopsy.

Evaluation of proposed diagnostic panels regarding clinical outcome revealed that all panels could, in addition, hold a prognostic potential. However, for a valid statement regarding prognostic impact, a bigger sample size and a longer follow-up period are essential, as the mean follow-up in our cohort was 14 months (range 2–18 months). Due to the lack of publicly available miRNA data from exosomes, prognostic evaluation in a bigger patient cohort is currently not feasible. Still, even from the small cohort, it became clear that exosomal miRNA profiles from the timepoint of diagnosis (treatment-naïve) hold prognostic potential independent of the therapy the patients received in course of tumor management.

Our study provides for the first time a comprehensive and exploratory comparison between miRNA profiles of plasma- and saliva-derived exosomes from HNSCC patients. We propose two panels of tumor-exclusive exosomal miRNAs, one plasma- and one saliva-derived, which hold great potential as liquid biopsy for discrimination between HNSCC patients and HD as well as HPV status and UICC stage. The provided panels present a promising tool for future liquid biomarker studies and—upon validation in a bigger cohort—can be diagnostically implemented in the clinical routine with the possibility to choose between saliva- or plasma-based diagnostic according to the patient’s need. The long-term aim is to routinely use saliva and plasma of patients in order to identify if a HNSCC is present and, if yes, determine the UICC stage (low or high) and the HPV status. This way, a faster diagnosis will be possible without the need to wait for the pathological statement of the tumor biopsy.

## Data Availability

The data presented in the study are deposited in the GEO repository, accession number GSE209670.
