# Supplementary material for: Comparison of plasma- and saliva-derived exosomal miRNA profiles reveals diagnostic potential in head and neck cancer
Source: Front Cell Dev Biol. 2022 Aug 22;10:971596. doi: 10.3389/fcell.2022.971596 (PMC9441766; doi:10.3389/fcell.2022.971596)
Supplement: Supplementary file 1 [file DataSheet1.pdf]

Supplementary Material

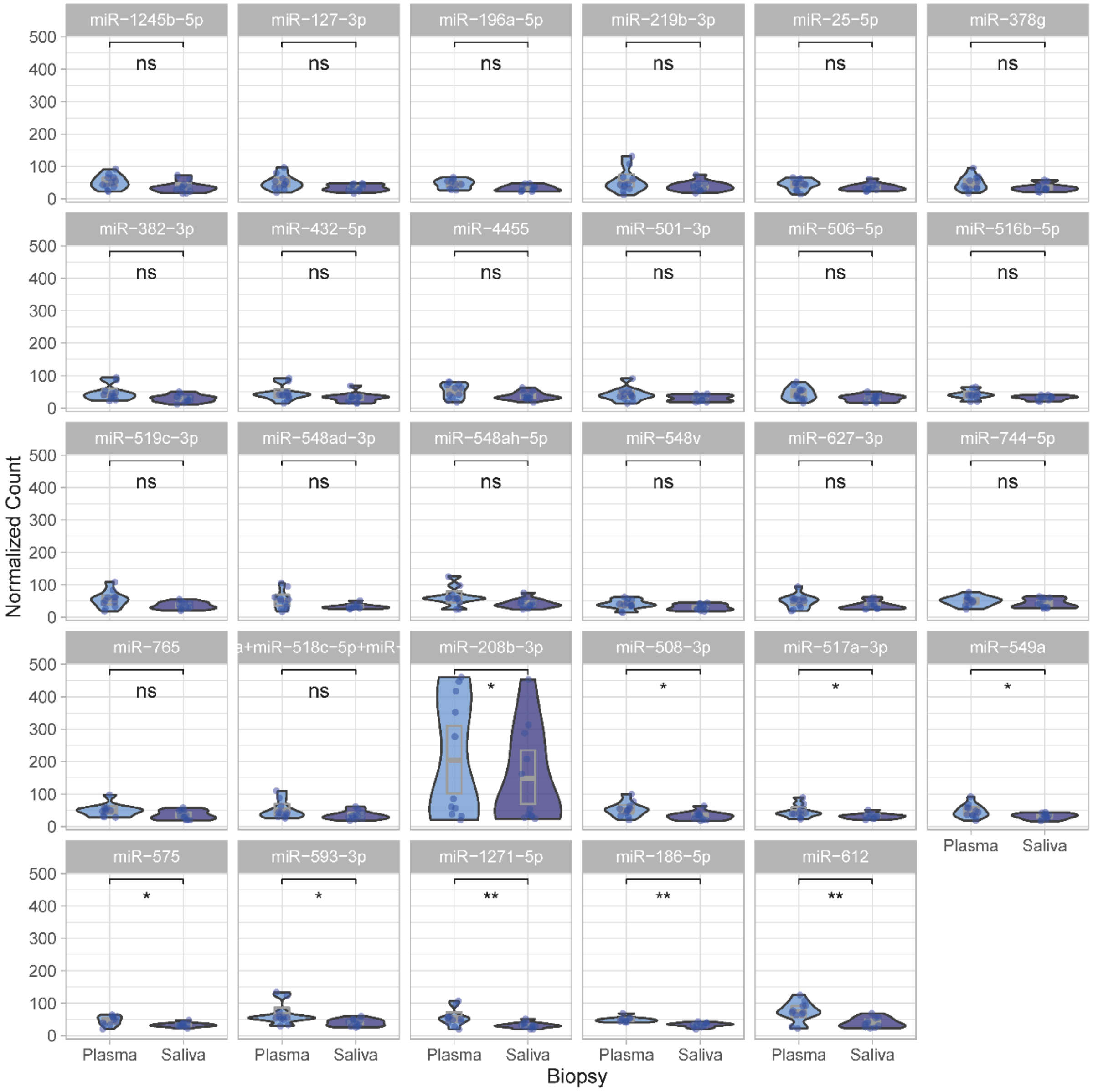

Supplementary Figure 1: Expression levels of tumor-exclusive exosomal miRNAs from plasma and saliva of HNSCC patients. Violin plots depicting the expression levels of tumor-exclusive exosomal miRNAs between plasma and saliva. Differential expression was calculated using paired Wilcoxon signed-rank test, with ns = not significant, * and ** corresponding to $p \leq 0.05$ and $p \leq 0.01$.

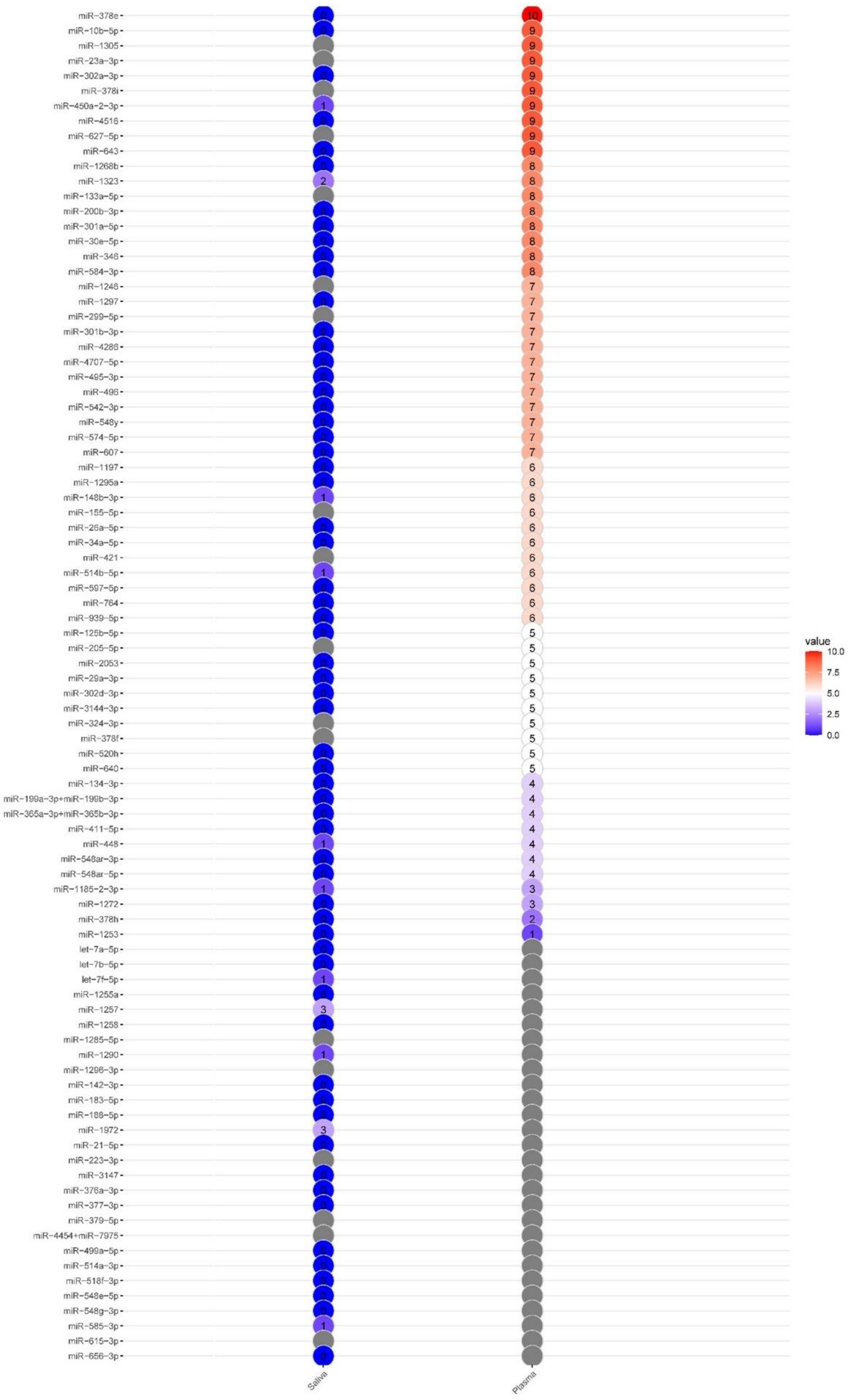

Supplementary Figure 2: Binarization of tumor-related exosomal miRNAs in plasma and saliva. Bubble plot of binarized tumor-related exosomal miRNAs. The number in the circles indicate the number of HNSCC patients (n = 11) positive for the individual miRNA. Grey bubbles have missing values in the binarization matrix and were not included into the sum.

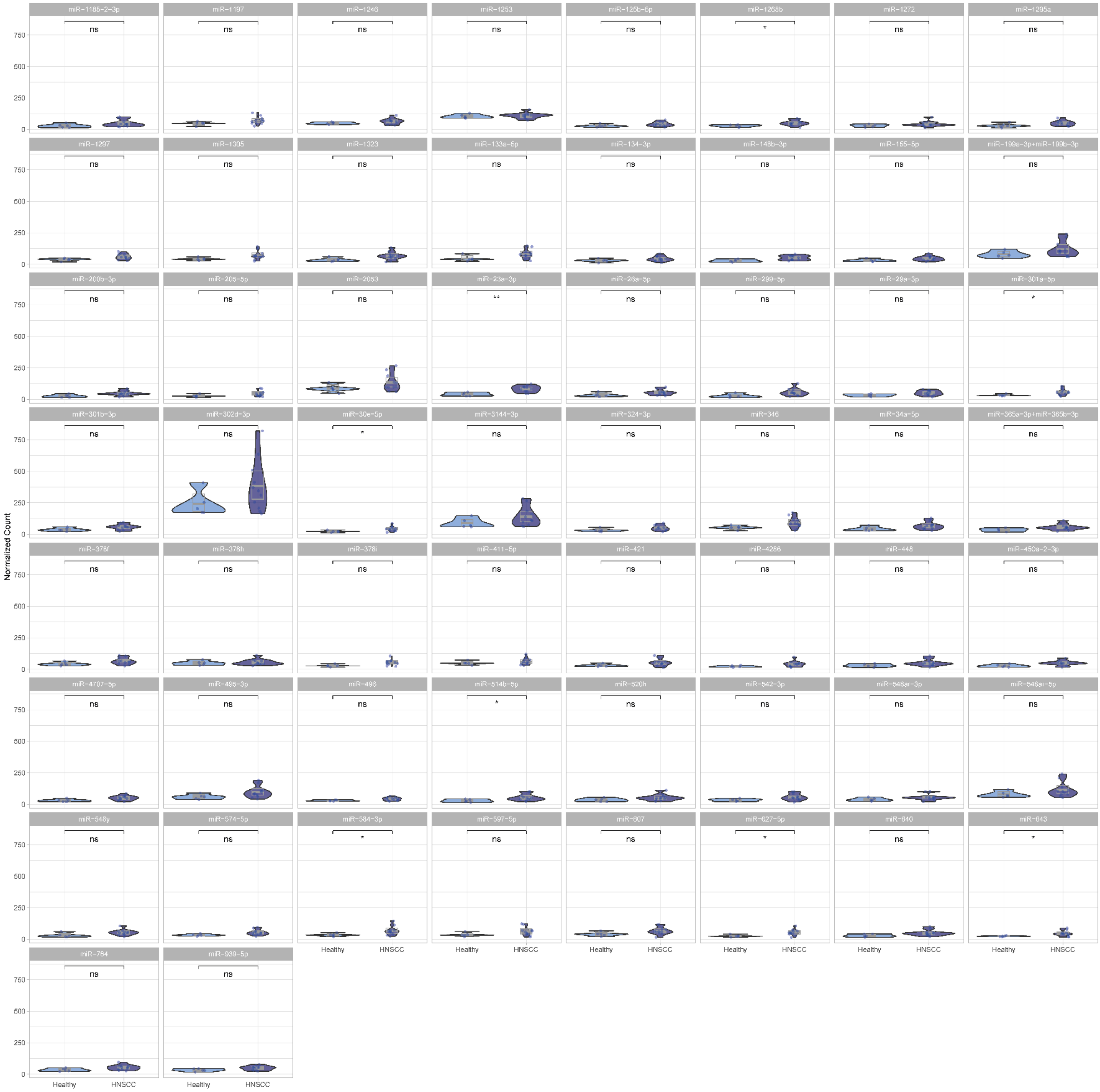

Supplementary Figure 3: Expression levels of tumor-upregulated exosomal miRNAs from plasma of HNSCC patients and healthy donors (HD). Violin plots depicting the expression levels of tumor-upregulated exosomal miRNAs from plasma between HNSCC patients and HD. Differential expression was calculated using unpaired Wilcoxon signed-rank test, with ns = not significant, * and ** corresponding to $p \leq 0.05$ and $p \leq 0.01$.

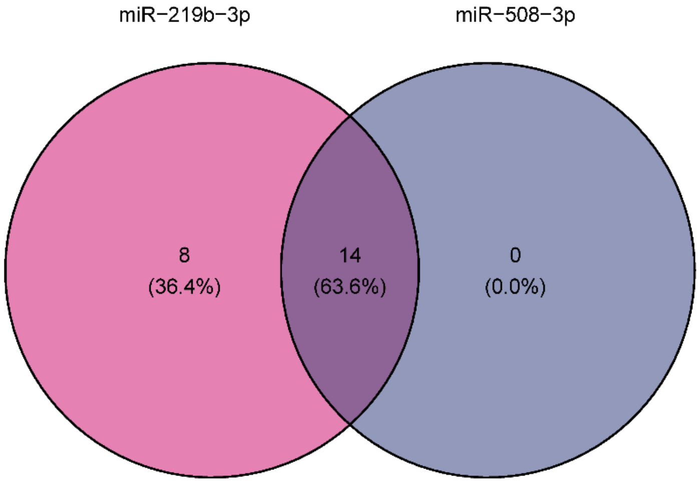

Supplementary Figure 4: Selection of diagnostic tumor-exclusive miRNA panel from plasma. Venn diagram depicting the two miRNAs with strongest correlation coefficient (miR-219b-3p, miR-508-3p) and the intersection of their co-expressed miRNAs. From the 14 overlapping miRNAs, 8 were selected for the panel based on strongest correlation (r ≥ 0.8).

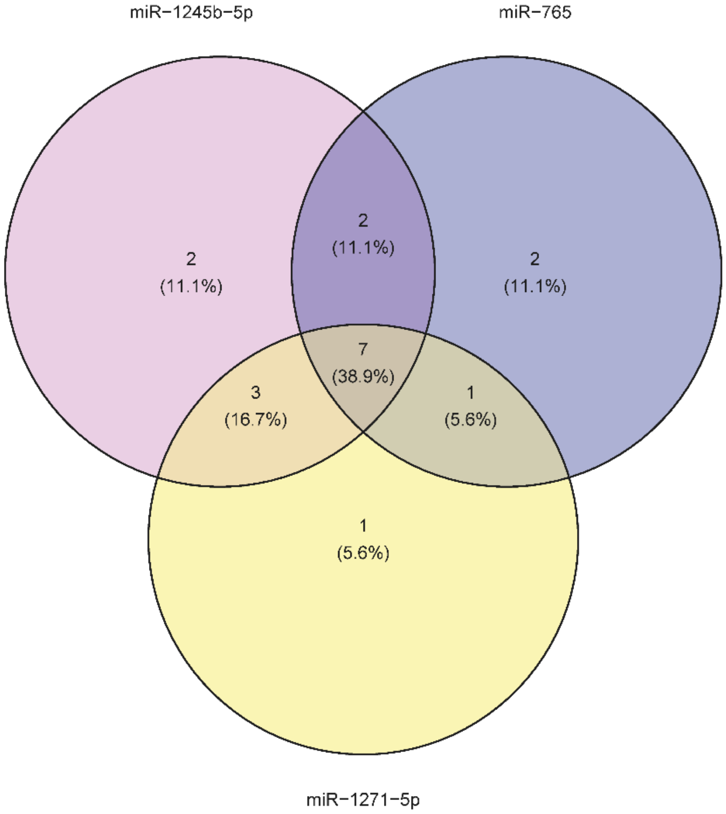

Supplementary Figure 5: Selection of diagnostic tumor-exclusive miRNA panel from saliva. Venn diagram depicting the three leading miRNAs with strongest correlation coefficient and lowest p-value (miR-1245b-5p, miR-765 and miR-1271-5p) and the intersection of their co-expressed miRNAs. The 7 overlapping miRNAs were included into the panel.

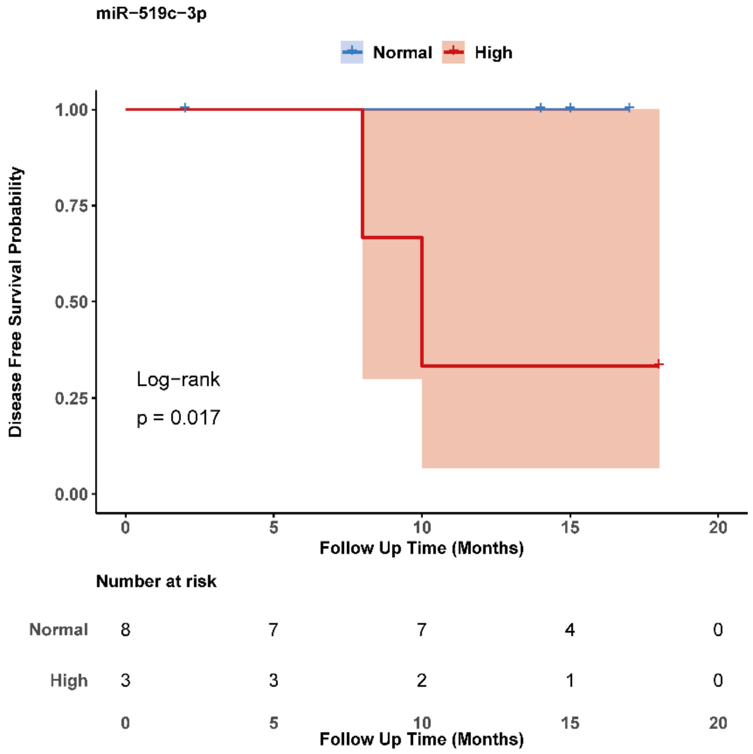

Supplementary Figure 6: Prognostic impact of exosomal miR-519c-3p from saliva of HNSCC patients. Kaplan-Meier plot with log-rank test for disease free-survival (DFS) of HNSCC patients binarized on their expression of saliva-derived exosomal miR-519c-3p. Blue indicates normal, red high expression and the shaded area represents the confidence interval.

Supplementary Table 1: Clinicopathological data of patients involved in this study

Characteristics	HNSCC patients (n = 11)	
	n	%
**Age (years)**		
Mean: 58		
Range: 49-64		
≤ 58	5	45
> 58	6	55
**Gender**		
Male	10	91
Female	1	9
**Primary tumor site**		
Oral cavity	1	9
Oropharynx	7	64
Hypopharynx	1	9
Larynx	2	18
**HPV status**		
Positive	4	36
Negative	6	55
Not determined	1	9
**Tumor stage**		
T1	2	18
T2	4	37
T3	3	27
T4	2	18
**Nodal status**		
N0	3	27
N+	8	73
**Distant metastasis**		
M0	11	100
**UICC grade**		
Low	5	45
High	6	55
**Recurrence**		
Yes	2	18
No	9	82
	Healthy donors (n = 5)	
**Age (years)**		
Mean: 53		
Range: 43-65		
**Gender**		
Male	4	80
Female	1	20

HPV: Human papillomavirus, UICC: Union for International Cancer Control

Supplementary Table 2: VENN diagram from Figure 3 in alphabetic order

Overlap Plasma and Saliva HNSCC			**Tumor-related miRNAs**		**Tumor-exclusive miRNAs**
let-7a-5p	miR-29a-3p	miR-526a+miR-518c-	let-7a-5p	miR-324-3p	miR-1245b-5p
let-7b-5p	miR-301a-5p	5p+miR-518d-5p	let-7b-5p	miR-346	miR-127-3p
let-7f-5p	miR-301b-3p	miR-542-3p	let-7f-5p	miR-34a-5p	miR-1271-5p
miR-10b-5p	miR-302a-3p	miR-548ad-3p	miR-10b-5p	miR-365a-3p+miR-	miR-186-5p
miR-1185-2-3p	miR-302d-3p	miR-548ah-5p	miR-1185-2-3p	365b-3p	miR-196a-5p
miR-1197	miR-30e-5p	miR-548ar-3p	miR-1197	miR-376a-3p	miR-208b-3p
miR-1245b-5p	miR-3144-3p	miR-548ar-5p	miR-1246	miR-377-3p	miR-219b-3p
miR-1246	miR-3147	miR-548e-5p	miR-1253	miR-378e	miR-25-5p
miR-1253	miR-324-3p	miR-548g-3p	miR-1255a	miR-378f	miR-378g
miR-1255a	miR-346	miR-548v	miR-1257	miR-378h	miR-382-3p
miR-1257	miR-34a-5p	miR-548y	miR-1258	miR-378i	miR-432-5p
miR-1258	miR-365a-3p+miR-	miR-549a	miR-125b-5p	miR-379-5p	miR-4455
miR-125b-5p	365b-3p	miR-574-5p	miR-1268b	miR-411-5p	miR-501-3p
miR-1268b	miR-376a-3p	miR-575	miR-1272	miR-421	miR-506-5p
miR-127-3p	miR-377-3p	miR-584-3p	miR-1285-5p	miR-4286	miR-508-3p
miR-1271-5p	miR-378e	miR-585-3p	miR-1290	miR-4454+miR-7975	miR-516b-5p
miR-1272	miR-378f	miR-593-3p	miR-1295a	miR-448	miR-517a-3p
miR-1285-5p	miR-378g	miR-597-5p	miR-1296-3p	miR-450a-2-3p	miR-519c-3p
miR-1290	miR-378h	miR-607	miR-1297	miR-4516	miR-526a+miR-518c-
miR-1295a	miR-378i	miR-612	miR-1305	miR-4707-5p	5p+miR-518d-5p
miR-1296-3p	miR-379-5p	miR-615-3p	miR-1323	miR-495-3p	miR-548ad-3p
miR-1297	miR-382-3p	miR-627-3p	miR-133a-5p	miR-496	miR-548ah-5p
miR-1305	miR-411-5p	miR-627-5p	miR-134-3p	miR-499a-5p	miR-548v
miR-1323	miR-421	miR-640	miR-142-3p	miR-514a-3p	miR-549a
miR-133a-5p	miR-4286	miR-643	miR-148b-3p	miR-514b-5p	miR-575
miR-134-3p	miR-432-5p	miR-656-3p	miR-155-5p	miR-518f-3p	miR-593-3p
miR-142-3p	miR-4454+miR-7975	miR-744-5p	miR-183-5p	miR-520h	miR-612
miR-148b-3p	miR-4455	miR-764	miR-188-5p	miR-542-3p	miR-627-3p
miR-155-5p	miR-448	miR-765	miR-1972	miR-548ar-3p	miR-744-5p

miR-183-5p<br>miR-186-5p<br>miR-188-5p<br>miR-196a-5p<br>miR-1972<br>miR-199a-3p+miR-199b-3p<br>miR-200b-3p<br>miR-205-5p<br>miR-2053<br>miR-208b-3p<br>miR-21-5p<br>miR-219b-3p<br>miR-223-3p<br>miR-23a-3p<br>miR-25-5p<br>miR-26a-5p<br>miR-299-5p<br>miR-450a-2-3p<br>miR-4516<br>miR-4707-5p<br>miR-495-3p<br>miR-496<br>miR-499a-5p<br>miR-501-3p<br>miR-506-5p<br>miR-508-3p<br>miR-514a-3p<br>miR-514b-5p<br>miR-516b-5p<br>miR-517a-3p<br>miR-518f-3p<br>miR-519c-3p<br>miR-520h<br>miR-939-5p	miR-199a-3p+miR-199b-3p<br>miR-200b-3p<br>miR-205-5p<br>miR-2053<br>miR-21-5p<br>miR-223-3p<br>miR-23a-3p<br>miR-26a-5p<br>miR-299-5p<br>miR-29a-3p<br>miR-301a-5p<br>miR-301b-3p<br>miR-302a-3p<br>miR-302d-3p<br>miR-30e-5p<br>miR-3144-3p<br>miR-3147<br>miR-548ar-5p<br>miR-548e-5p<br>miR-548g-3p<br>miR-548y<br>miR-574-5p<br>miR-584-3p<br>miR-585-3p<br>miR-597-5p<br>miR-607<br>miR-615-3p<br>miR-627-5p<br>miR-640<br>miR-643<br>miR-656-3p<br>miR-764<br>miR-939-5p	miR-765

Supplementary Table 3: Binarization of tumor-related exosomal miRNAs in plasma

miRNA	Cut off	1	2	3	4	5	6	7	8	9	10	11	Sum	Percentage
let-7a-5p	75.51	NA	0	1	1	1	1	1	1	1	1	NA	NA	NA
let-7b-5p	59.22	1	0	1	1	1	1	1	1	1	1	NA	NA	NA
let-7f-5p	58.88	0	1	0	1	1	0	1	0	1	1	NA	NA	NA
miR-10b-5p	24.79	1	1	1	1	1	0	1	0	1	1	1	9	69.23
miR-1185-2-3p	46.29	0	0	0	0	0	0	1	0	1	0	1	3	23.08
miR-1197	65.09	0	1	1	1	0	0	0	0	1	1	1	6	46.15
miR-1246	59.78	1	1	0	1	0	0	1	0	1	1	1	7	53.85
miR-1253	124.38	0	0	0	0	0	0	0	0	0	0	1	1	7.69
miR-1255a	71.06	0	0	0	0	0	0	1	0	NA	0	NA	NA	NA
miR-1257	45.01	0	1	0	1	1	0	1	0	NA	1	NA	NA	NA
miR-1258	53.83	0	1	0	1	0	0	1	0	NA	0	NA	NA	NA
miR-125b-5p	43.01	0	1	0	1	1	0	0	0	1	0	1	5	38.46
miR-1268b	41.27	0	1	1	1	0	1	1	0	1	1	1	8	61.54
miR-1272	46.40	0	0	0	1	0	0	0	0	1	0	1	3	23.08
miR-1285-5p	68.18	0	1	0	1	1	0	1	0	NA	1	1	NA	NA
miR-1290	65.32	0	1	0	1	0	0	0	0	1	0	NA	NA	NA
miR-1295a	49.04	0	0	0	1	1	0	1	0	1	1	1	6	46.15
miR-1296-3p	46.93	1	1	0	1	1	1	1	0	NA	1	NA	NA	NA
miR-1297	53.41	0	1	0	1	1	0	1	0	1	1	1	7	53.85
miR-1305	53.81	0	1	1	1	1	1	1	0	1	1	1	9	69.23
miR-1323	54.07	0	1	1	1	1	0	1	0	1	1	1	8	61.54
miR-133a-5p	67.76	0	1	1	1	1	0	1	0	1	1	1	8	61.54
miR-134-3p	46.15	0	0	1	0	0	0	1	0	1	0	1	4	30.77
miR-142-3p	98.31	NA	1	1	1	1	1	1	1	1	1	1	NA	NA
miR-148b-3p	45.94	0	1	1	1	0	0	1	0	1	0	1	6	46.15
miR-155-5p	44.33	0	1	0	1	1	0	1	0	1	0	1	6	46.15

miR-183-5p	68.62	0	1	1	1	0	0	1	0	NA	1	1	NA	NA
miR-188-5p	64.07	0	1	0	1	0	0	1	0	NA	0	NA	NA	NA
miR-1972	30.23	0	1	1	1	1	1	1	0	1	1	NA	NA	NA
miR-199a-3p+miR-199b-3p	113.92	1	0	0	0	0	0	1	0	1	0	1	4	30.77
miR-200b-3p	40.40	0	1	1	1	1	1	1	0	1	0	1	8	61.54
miR-205-5p	39.40	0	1	0	1	0	0	1	0	1	0	1	5	38.46
miR-2053	119.01	0	0	0	1	1	0	1	0	1	0	1	5	38.46
miR-21-5p	49.52	1	1	0	0	0	0	1	0	NA	0	NA	NA	NA
miR-223-3p	155.38	NA	0	1	1	1	0	1	1	0	0	1	NA	NA
miR-23a-3p	60.01	1	1	1	1	1	0	1	0	1	1	1	9	69.23
miR-26a-5p	52.92	1	1	0	0	0	0	1	0	1	1	1	6	46.15
miR-299-5p	47.07	0	1	1	0	1	0	1	0	1	1	1	7	53.85
miR-29a-3p	47.66	0	1	0	0	1	0	1	0	1	0	1	5	38.46
miR-301a-5p	40.52	0	1	1	1	1	0	1	0	1	1	1	8	61.54
miR-301b-3p	54.44	0	1	1	1	0	0	1	0	1	1	1	7	53.85
miR-302a-3p	28.11	0	1	1	1	1	1	1	0	1	1	1	9	69.23
miR-302d-3p	345.64	0	1	0	0	1	0	1	0	1	0	1	5	38.46
miR-30e-5p	30.20	0	1	1	1	1	0	1	0	1	1	1	8	61.54
miR-3144-3p	137.54	0	0	0	1	1	0	1	0	1	0	1	5	38.46
miR-3147	46.67	0	1	0	1	0	0	1	0	1	1	NA	NA	NA
miR-324-3p	46.06	0	1	0	1	0	0	1	0	1	0	1	5	38.46
miR-346	72.60	0	1	1	1	1	0	1	0	1	1	1	8	61.54
miR-34a-5p	64.01	0	1	1	1	0	0	1	0	1	0	1	6	46.15
miR-365a-3p+miR-365b-3p	55.28	0	0	0	1	0	0	0	0	1	1	1	4	30.77
miR-376a-3p	71.36	0	0	0	0	1	0	1	0	1	0	NA	NA	NA
miR-377-3p	54.92	0	1	0	0	0	0	0	0	NA	1	NA	NA	NA
miR-378e	90.54	1	1	1	1	1	1	1	0	1	1	1	10	76.92
miR-378f	59.45	0	0	0	1	1	0	1	0	1	0	1	5	38.46

miR-378h	69.63	0	1	0	0	0	0	0	0	0	0	1	2	15.38
miR-378i	38.45	0	1	1	1	1	1	1	0	1	1	1	9	69.23
miR-379-5p	41.44	0	1	1	1	1	0	1	0	NA	0	NA	NA	NA
miR-411-5p	65.05	0	1	0	0	0	0	1	0	1	0	1	4	30.77
miR-421	43.85	0	1	0	1	0	0	1	0	1	1	1	6	46.15
miR-4286	31.95	0	1	0	1	1	0	1	0	1	1	1	7	53.85
miR-4454+miR-7975	31.54	1	0	1	1	1	1	1	0	1	1	NA	NA	NA
miR-448	48.41	0	0	0	0	0	0	1	0	1	1	1	4	30.77
miR-450a-2-3p	41.66	0	1	1	1	1	1	1	0	1	1	1	9	69.23
miR-4516	21.49	0	1	1	1	1	1	1	0	1	1	1	9	69.23
miR-4707-5p	43.17	0	1	0	1	1	0	1	0	1	1	1	7	53.85
miR-495-3p	81.74	0	1	1	1	0	0	1	0	1	1	1	7	53.85
miR-496	36.76	0	1	1	1	1	0	0	0	1	1	1	7	53.85
miR-499a-5p	79.57	0	0	0	0	0	0	1	0	1	0	NA	NA	NA
miR-514a-3p	49.16	0	1	0	1	0	0	0	0	NA	0	1	NA	NA
miR-514b-5p	44.73	0	1	0	1	1	0	1	0	1	0	1	6	46.15
miR-518f-3p	50.85	0	1	0	1	0	0	1	0	1	1	NA	NA	NA
miR-520h	51.81	0	1	0	0	1	0	1	0	1	0	1	5	38.46
miR-542-3p	48.14	0	1	1	1	0	0	1	0	1	1	1	7	53.85
miR-548ar-3p	54.96	0	0	0	1	0	0	0	0	1	1	1	4	30.77
miR-548ar-5p	107.49	0	0	0	0	1	0	1	0	1	0	1	4	30.77
miR-548e-5p	62.96	0	1	1	0	0	0	1	0	1	1	NA	NA	NA
miR-548g-3p	53.08	0	0	0	0	0	0	1	0	1	0	NA	NA	NA
miR-548y	48.54	0	1	1	1	1	0	1	0	1	0	1	7	53.85
miR-574-5p	43.58	0	1	1	1	1	0	0	0	1	1	1	7	53.85
miR-584-3p	46.26	0	1	1	1	1	0	1	0	1	1	1	8	61.54
miR-585-3p	75.39	0	0	0	0	0	0	1	0	1	0	NA	NA	NA
miR-597-5p	51.42	0	1	1	0	0	0	1	0	1	1	1	6	46.15

miR-607	58.92	0	1	1	1	1	0	1	0	1	0	1	7	53.85
miR-615-3p	65.53	0	1	0	0	0	0	0	0	NA	0	NA	NA	NA
miR-627-5p	34.47	0	1	1	1	1	1	1	0	1	1	1	9	69.23
miR-640	47.97	0	1	0	0	1	0	1	0	1	0	1	5	38.46
miR-643	29.45	0	1	1	1	1	1	1	0	1	1	1	9	69.23
miR-656-3p	36.13	1	1	1	1	1	1	1	0	NA	1	NA	NA	NA
miR-764	51.15	0	1	0	1	0	0	1	0	1	1	1	6	46.15
miR-939-5p	49.61	0	1	0	1	1	0	1	0	1	0	1	6	46.15

Supplementary Table 4: Binarization of tumor-related exosomal miRNAs in saliva

miRNAs	Cut off	1	2	3	4	5	6	7	8	9	10	11	Sum	Percentage
let-7a-5p	64.88	0	0	0	0	0	0	0	0	0	0	0	0	0.00
let-7b-5p	67.02	0	0	0	0	0	0	0	0	0	0	0	0	0.00
let-7f-5p	69.41	0	0	0	0	0	0	0	0	0	1	0	1	7.69
miR-10b-5p	64.34	0	0	0	0	0	0	0	0	0	0	0	0	0.00
miR-1185-2-3p	47.62	0	0	0	0	0	0	0	0	1	0	0	1	7.69
miR-1197	84.88	0	0	0	0	0	0	0	0	0	0	0	0	0.00
miR-1246	313.03	0	0	0	0	NA	0	0	0	0	0	0	NA	NA
miR-1253	237.33	0	0	0	0	0	0	0	0	0	0	0	0	0.00
miR-1255a	99.85	0	0	0	0	0	0	0	0	0	0	0	0	0.00
miR-1257	41.68	0	0	0	0	0	0	1	0	1	1	0	3	23.08
miR-1258	86.93	0	0	0	0	0	0	0	0	0	0	0	0	0.00
miR-125b-5p	126.49	0	0	0	0	0	0	0	0	0	0	0	0	0.00
miR-1268b	86.97	0	0	0	0	0	0	0	0	0	0	0	0	0.00
miR-1272	49.62	0	0	0	0	0	0	0	0	0	0	0	0	0.00
miR-1285-5p	164.17	0	0	0	0	0	0	NA	0	NA	NA	0	NA	NA
miR-1290	81.55	0	0	0	0	0	0	0	0	0	1	0	1	7.69
miR-1295a	70.01	0	0	0	0	0	0	0	0	0	0	0	0	0.00
miR-1296-3p	99.91	0	0	0	0	0	0	0	0	NA	NA	0	NA	NA
miR-1297	92.38	0	0	0	0	0	0	0	0	0	0	0	0	0.00
miR-1305	87.61	0	0	0	0	0	0	0	0	NA	0	0	NA	NA
miR-1323	55.95	0	0	0	0	0	0	1	0	0	1	0	2	15.38
miR-133a-5p	89.73	0	0	0	0	0	0	0	0	NA	0	0	NA	NA
miR-134-3p	55.66	0	0	0	0	0	0	0	0	0	0	0	0	0.00
miR-142-3p	126.12	0	0	0	0	0	0	0	0	0	0	0	0	0.00
miR-148b-3p	44.65	0	0	0	0	0	0	0	0	1	0	0	1	7.69
miR-155-5p	210.62	NA	0	0	0	0	0	0	NA	0	0	NA	NA	NA

miR-183-5p	113.83	0	0	0	0	0	0	0	0	0	0	0	0	0.00
miR-188-5p	79.46	0	0	0	0	0	0	0	0	0	0	0	0	0.00
miR-1972	48.30	0	1	0	0	0	0	0	0	1	1	0	3	23.08
miR-199a-3p+miR-199b-3p	156.82	0	0	0	0	0	0	0	0	0	0	0	0	0.00
miR-200b-3p	80.41	0	0	0	0	0	0	0	0	0	0	0	0	0.00
miR-205-5p	231.46	0	0	0	0	0	0	0	0	0	0	NA	NA	NA
miR-2053	133.86	0	0	0	0	0	0	0	0	0	0	0	0	0.00
miR-21-5p	92.87	0	0	0	0	0	0	0	0	0	0	0	0	0.00
miR-223-3p	1075.59	0	0	0	0	NA	0	0	0	0	0	0	NA	NA
miR-23a-3p	238.89	0	0	0	0	NA	0	0	0	0	0	0	NA	NA
miR-26a-5p	67.23	0	0	0	0	0	0	0	0	0	0	0	0	0.00
miR-299-5p	67.31	0	0	0	0	0	0	0	0	NA	NA	0	NA	NA
miR-29a-3p	81.84	0	0	0	0	0	0	0	0	0	0	0	0	0.00
miR-301a-5p	85.78	0	0	0	0	0	0	0	0	0	0	0	0	0.00
miR-301b-3p	81.41	0	0	0	0	0	0	0	0	0	0	0	0	0.00
miR-302a-3p	60.30	0	0	0	0	0	0	0	0	0	0	0	0	0.00
miR-302d-3p	594.62	0	0	0	0	0	0	0	0	0	0	0	0	0.00
miR-30e-5p	50.91	0	0	0	0	0	0	0	0	0	0	0	0	0.00
miR-3144-3p	161.71	0	0	0	0	0	0	0	0	0	0	0	0	0.00
miR-3147	79.06	0	0	0	0	0	0	0	0	0	0	0	0	0.00
miR-324-3p	103.53	0	0	0	0	0	0	0	0	0	NA	0	NA	NA
miR-346	119.56	0	0	0	0	0	0	0	0	0	0	0	0	0.00
miR-34a-5p	121.04	0	0	0	0	0	0	0	0	0	0	0	0	0.00
miR-365a-3p+miR-365b-3p	101.74	0	0	0	0	0	0	0	0	0	0	0	0	0.00
miR-376a-3p	70.72	0	0	0	0	0	0	0	0	0	0	0	0	0.00
miR-377-3p	81.81	0	0	0	0	0	0	0	0	0	0	0	0	0.00
miR-378e	319.07	0	0	0	0	0	0	0	0	0	0	0	0	0.00
miR-378f	109.42	0	0	0	0	0	0	0	0	NA	0	0	NA	NA

miR-378h	99.67	0	0	0	0	0	0	0	0	0	0	0	0	0.00
miR-378i	72.14	0	0	0	0	0	0	0	0	0	NA	0	NA	NA
miR-379-5p	65.21	0	0	0	0	0	0	0	0	0	NA	0	NA	NA
miR-411-5p	91.98	0	0	0	0	0	0	0	0	0	0	0	0	0.00
miR-421	64.69	0	0	NA	0	0	0	0	0	NA	NA	0	NA	NA
miR-4286	50.72	0	0	0	0	0	0	0	0	0	0	0	0	0.00
miR-4454+miR-7975	110.37	0	0	0	0	0	0	NA	0	1	0	0	NA	NA
miR-448	47.26	0	0	0	0	0	0	0	0	1	0	0	1	7.69
miR-450a-2-3p	70.37	0	0	0	0	0	0	0	0	1	0	0	1	7.69
miR-4516	208.15	0	0	0	0	0	0	0	0	0	0	0	0	0.00
miR-4707-5p	83.48	0	0	0	0	0	0	0	0	0	0	0	0	0.00
miR-495-3p	163.50	0	0	0	0	0	0	0	0	0	0	0	0	0.00
miR-496	55.32	0	0	0	0	0	0	0	0	0	0	0	0	0.00
miR-499a-5p	88.25	0	0	0	0	0	0	0	0	0	0	0	0	0.00
miR-514a-3p	82.23	0	0	0	0	0	0	0	0	0	0	0	0	0.00
miR-514b-5p	59.03	0	0	0	0	0	0	0	0	0	1	0	1	7.69
miR-518f-3p	74.69	0	0	0	0	0	0	0	0	0	0	0	0	0.00
miR-520h	78.08	0	0	0	0	0	0	0	0	0	0	0	0	0.00
miR-542-3p	89.40	0	0	0	0	0	0	0	0	0	0	0	0	0.00
miR-548ar-3p	103.77	0	0	0	0	0	0	0	0	0	0	0	0	0.00
miR-548ar-5p	126.54	0	0	0	0	0	0	0	0	0	0	0	0	0.00
miR-548e-5p	104.62	0	0	0	0	0	0	0	0	0	0	0	0	0.00
miR-548g-3p	70.70	0	0	0	0	0	0	0	0	0	0	0	0	0.00
miR-548y	86.42	0	0	0	0	0	0	0	0	0	0	0	0	0.00
miR-574-5p	84.65	0	0	0	0	0	0	0	0	0	0	0	0	0.00
miR-584-3p	91.53	0	0	0	0	0	0	0	0	0	0	0	0	0.00
miR-585-3p	74.38	0	0	0	0	0	0	0	0	1	0	0	1	7.69
miR-597-5p	76.01	0	0	0	0	0	0	0	0	0	0	0	0	0.00

miR-607	70.52	0	0	0	0	0	0	0	0	0	0	0	0	0.00
miR-615-3p	116.53	0	0	0	0	0	0	0	0	NA	NA	0	NA	NA
miR-627-5p	47.02	0	0	0	0	0	0	0	0	1	NA	0	NA	NA
miR-640	53.05	0	0	0	0	0	0	0	0	0	0	0	0	0.00
miR-643	62.53	0	0	0	0	0	0	0	0	0	0	0	0	0.00
miR-656-3p	73.79	0	0	0	0	0	0	0	0	0	0	0	0	0.00
miR-764	85.18	0	0	0	0	0	0	0	0	0	0	0	0	0.00
miR-939-5p	59.34	0	0	0	0	0	0	0	0	0	0	0	0	0.00

Supplementary Table 5: Intra-correlation and clustering of tumor-exclusive exosomal miRNAs in plasma

Row	Column	Correlation coefficient	p-value
miR-1245b-5p	miR-127-3p	0.8929	2.15E-04
miR-1245b-5p	miR-1271-5p	0.8829	3.17E-04
miR-127-3p	miR-1271-5p	0.9212	5.64E-05
miR-1245b-5p	miR-186-5p	0.6585	5.38E-02
miR-127-3p	miR-186-5p	0.6526	5.67E-02
miR-1271-5p	miR-186-5p	0.7822	1.27E-02
miR-1245b-5p	miR-196a-5p	0.5550	1.21E-01
miR-127-3p	miR-196a-5p	0.7763	1.39E-02
miR-1271-5p	miR-196a-5p	0.8973	1.03E-03
miR-186-5p	miR-196a-5p	-0.2250	5.92E-01
miR-1245b-5p	miR-208b-3p	0.1201	7.25E-01
miR-127-3p	miR-208b-3p	-0.2416	4.74E-01
miR-1271-5p	miR-208b-3p	-0.1254	7.13E-01
miR-186-5p	miR-208b-3p	-0.0227	9.54E-01
miR-196a-5p	miR-208b-3p	0.1451	7.10E-01
miR-1245b-5p	miR-219b-3p	0.8939	2.07E-04
miR-127-3p	miR-219b-3p	0.9625	2.12E-06
miR-1271-5p	miR-219b-3p	0.9394	1.79E-05
miR-186-5p	miR-219b-3p	0.7106	3.19E-02
miR-196a-5p	miR-219b-3p	0.8172	7.15E-03
miR-208b-3p	miR-219b-3p	-0.1665	6.25E-01
miR-1245b-5p	miR-25-5p	0.8768	3.96E-04
miR-127-3p	miR-25-5p	0.8899	2.43E-04
miR-1271-5p	miR-25-5p	0.8201	1.99E-03
miR-186-5p	miR-25-5p	0.5405	1.33E-01
miR-196a-5p	miR-25-5p	0.6911	3.93E-02
miR-208b-3p	miR-25-5p	-0.0142	9.67E-01
miR-219b-3p	miR-25-5p	0.8324	1.48E-03
miR-1245b-5p	miR-378g	0.8093	2.55E-03
miR-127-3p	miR-378g	0.8352	1.37E-03
miR-1271-5p	miR-378g	0.8293	1.60E-03
miR-186-5p	miR-378g	0.7275	2.63E-02
miR-196a-5p	miR-378g	0.3815	3.11E-01
miR-208b-3p	miR-378g	-0.1490	6.62E-01
miR-219b-3p	miR-378g	0.8226	1.88E-03
miR-25-5p	miR-378g	0.8451	1.06E-03
miR-1245b-5p	miR-382-3p	0.8930	2.15E-04
miR-127-3p	miR-382-3p	0.9322	2.92E-05
miR-1271-5p	miR-382-3p	0.9457	1.10E-05
miR-186-5p	miR-382-3p	0.7820	1.28E-02
miR-196a-5p	miR-382-3p	0.7549	1.87E-02
miR-208b-3p	miR-382-3p	-0.1579	6.43E-01
miR-219b-3p	miR-382-3p	0.9801	1.25E-07

miR-25-5p	miR-382-3p	0.8430	1.12E-03
miR-378g	miR-382-3p	0.8411	1.18E-03
miR-1245b-5p	miR-432-5p	0.8201	1.99E-03
miR-127-3p	miR-432-5p	0.8880	2.61E-04
miR-1271-5p	miR-432-5p	0.9628	2.05E-06
miR-186-5p	miR-432-5p	0.7986	9.84E-03
miR-196a-5p	miR-432-5p	0.7557	1.85E-02
miR-208b-3p	miR-432-5p	-0.1671	6.23E-01
miR-219b-3p	miR-432-5p	0.9145	8.06E-05
miR-25-5p	miR-432-5p	0.8107	2.47E-03
miR-378g	miR-432-5p	0.8463	1.02E-03
miR-382-3p	miR-432-5p	0.9484	8.75E-06
miR-1245b-5p	miR-4455	0.8139	2.30E-03
miR-127-3p	miR-4455	0.6459	3.18E-02
miR-1271-5p	miR-4455	0.7243	1.17E-02
miR-186-5p	miR-4455	0.5292	1.43E-01
miR-196a-5p	miR-4455	0.5830	9.95E-02
miR-208b-3p	miR-4455	0.4408	1.75E-01
miR-219b-3p	miR-4455	0.7279	1.11E-02
miR-25-5p	miR-4455	0.7785	4.77E-03
miR-378g	miR-4455	0.6946	1.77E-02
miR-382-3p	miR-4455	0.7432	8.77E-03
miR-432-5p	miR-4455	0.6695	2.42E-02
miR-1245b-5p	miR-501-3p	0.8514	8.84E-04
miR-127-3p	miR-501-3p	0.8099	2.51E-03
miR-1271-5p	miR-501-3p	0.7968	3.32E-03
miR-186-5p	miR-501-3p	0.6725	4.72E-02
miR-196a-5p	miR-501-3p	0.5583	1.18E-01
miR-208b-3p	miR-501-3p	0.1489	6.62E-01
miR-219b-3p	miR-501-3p	0.8624	6.36E-04
miR-25-5p	miR-501-3p	0.7615	6.47E-03
miR-378g	miR-501-3p	0.5689	6.78E-02
miR-382-3p	miR-501-3p	0.8725	4.58E-04
miR-432-5p	miR-501-3p	0.7955	3.41E-03
miR-4455	miR-501-3p	0.7292	1.09E-02
miR-1245b-5p	miR-506-5p	0.8516	8.79E-04
miR-127-3p	miR-506-5p	0.8519	8.72E-04
miR-1271-5p	miR-506-5p	0.9001	1.59E-04
miR-186-5p	miR-506-5p	0.6340	6.67E-02
miR-196a-5p	miR-506-5p	0.7375	2.33E-02
miR-208b-3p	miR-506-5p	0.0200	9.53E-01
miR-219b-3p	miR-506-5p	0.9148	7.95E-05
miR-25-5p	miR-506-5p	0.8636	6.12E-04
miR-378g	miR-506-5p	0.8362	1.34E-03
miR-382-3p	miR-506-5p	0.9171	7.04E-05
miR-432-5p	miR-506-5p	0.8582	7.24E-04
miR-4455	miR-506-5p	0.8955	1.94E-04

miR-501-3p	miR-506-5p	0.7754	5.05E-03
miR-1245b-5p	miR-508-3p	0.9536	5.46E-06
miR-127-3p	miR-508-3p	0.9407	1.62E-05
miR-1271-5p	miR-508-3p	0.9290	3.57E-05
miR-186-5p	miR-508-3p	0.6618	5.22E-02
miR-196a-5p	miR-508-3p	0.7884	1.16E-02
miR-208b-3p	miR-508-3p	-0.0324	9.25E-01
miR-219b-3p	miR-508-3p	0.9434	1.32E-05
miR-25-5p	miR-508-3p	0.8811	3.39E-04
miR-378g	miR-508-3p	0.7438	8.69E-03
miR-382-3p	miR-508-3p	0.9362	2.24E-05
miR-432-5p	miR-508-3p	0.8745	4.28E-04
miR-4455	miR-508-3p	0.7495	7.92E-03
miR-501-3p	miR-508-3p	0.9127	8.83E-05
miR-506-5p	miR-508-3p	0.8746	4.27E-04
miR-1245b-5p	miR-516b-5p	0.7384	1.47E-02
miR-127-3p	miR-516b-5p	0.9250	1.27E-04
miR-1271-5p	miR-516b-5p	0.8052	4.94E-03
miR-186-5p	miR-516b-5p	0.4291	2.49E-01
miR-196a-5p	miR-516b-5p	0.8856	1.49E-03
miR-208b-3p	miR-516b-5p	-0.0772	8.32E-01
miR-219b-3p	miR-516b-5p	0.8496	1.86E-03
miR-25-5p	miR-516b-5p	0.9015	3.65E-04
miR-378g	miR-516b-5p	0.7659	9.79E-03
miR-382-3p	miR-516b-5p	0.8124	4.29E-03
miR-432-5p	miR-516b-5p	0.7856	7.07E-03
miR-4455	miR-516b-5p	0.6706	3.38E-02
miR-501-3p	miR-516b-5p	0.6405	4.60E-02
miR-506-5p	miR-516b-5p	0.7995	5.51E-03
miR-508-3p	miR-516b-5p	0.8311	2.89E-03
miR-1245b-5p	miR-517a-3p	0.8760	8.89E-04
miR-127-3p	miR-517a-3p	0.8511	1.79E-03
miR-1271-5p	miR-517a-3p	0.8638	1.27E-03
miR-186-5p	miR-517a-3p	0.7895	1.14E-02
miR-196a-5p	miR-517a-3p	0.4626	2.10E-01
miR-208b-3p	miR-517a-3p	0.1449	6.90E-01
miR-219b-3p	miR-517a-3p	0.9413	4.84E-05
miR-25-5p	miR-517a-3p	0.7617	1.05E-02
miR-378g	miR-517a-3p	0.8997	3.91E-04
miR-382-3p	miR-517a-3p	0.9093	2.65E-04
miR-432-5p	miR-517a-3p	0.7917	6.35E-03
miR-4455	miR-517a-3p	0.8420	2.25E-03
miR-501-3p	miR-517a-3p	0.6953	2.56E-02
miR-506-5p	miR-517a-3p	0.9178	1.80E-04
miR-508-3p	miR-517a-3p	0.8365	2.55E-03
miR-516b-5p	miR-517a-3p	0.7049	2.28E-02
miR-1245b-5p	miR-519c-3p	0.8951	1.97E-04

miR-127-3p	miR-519c-3p	0.8718	4.70E-04
miR-1271-5p	miR-519c-3p	0.8819	3.29E-04
miR-186-5p	miR-519c-3p	0.7827	1.26E-02
miR-196a-5p	miR-519c-3p	0.5526	1.23E-01
miR-208b-3p	miR-519c-3p	0.0103	9.76E-01
miR-219b-3p	miR-519c-3p	0.8781	3.77E-04
miR-25-5p	miR-519c-3p	0.8510	8.95E-04
miR-378g	miR-519c-3p	0.7155	1.33E-02
miR-382-3p	miR-519c-3p	0.9181	6.69E-05
miR-432-5p	miR-519c-3p	0.9048	1.29E-04
miR-4455	miR-519c-3p	0.6903	1.87E-02
miR-501-3p	miR-519c-3p	0.9432	1.34E-05
miR-506-5p	miR-519c-3p	0.7886	3.92E-03
miR-508-3p	miR-519c-3p	0.9416	1.51E-05
miR-516b-5p	miR-519c-3p	0.7412	1.42E-02
miR-517a-3p	miR-519c-3p	0.7073	2.22E-02
miR-1245b-5p	miR-526a+518c-5p+518d-5p	0.6547	3.99E-02
miR-127-3p	miR-526a+518c-5p+518d-5p	0.9191	1.70E-04
miR-1271-5p	miR-526a+518c-5p+518d-5p	0.8641	1.26E-03
miR-186-5p	miR-526a+518c-5p+518d-5p	0.5326	1.40E-01
miR-196a-5p	miR-526a+518c-5p+518d-5p	0.8442	4.20E-03
miR-208b-3p	miR-526a+518c-5p+518d-5p	-0.3702	2.92E-01
miR-219b-3p	miR-526a+518c-5p+518d-5p	0.9003	3.83E-04
miR-25-5p	miR-526a+518c-5p+518d-5p	0.7981	5.66E-03
miR-378g	miR-526a+518c-5p+518d-5p	0.8223	3.50E-03
miR-382-3p	miR-526a+518c-5p+518d-5p	0.8871	6.18E-04
miR-432-5p	miR-526a+518c-5p+518d-5p	0.8583	1.48E-03
miR-4455	miR-526a+518c-5p+518d-5p	0.5510	9.88E-02
miR-501-3p	miR-526a+518c-5p+518d-5p	0.5012	1.40E-01
miR-506-5p	miR-526a+518c-5p+518d-5p	0.8205	3.64E-03
miR-508-3p	miR-526a+518c-5p+518d-5p	0.7882	6.75E-03
miR-516b-5p	miR-526a+518c-5p+518d-5p	0.9084	2.76E-04
miR-517a-3p	miR-526a+518c-5p+518d-5p	0.7368	1.51E-02
miR-519c-3p	miR-526a+518c-5p+518d-5p	0.6813	3.01E-02
miR-1245b-5p	miR-548ad-3p	0.8878	2.63E-04
miR-127-3p	miR-548ad-3p	0.8826	3.21E-04
miR-1271-5p	miR-548ad-3p	0.9465	1.03E-05
miR-186-5p	miR-548ad-3p	0.7805	1.31E-02
miR-196a-5p	miR-548ad-3p	0.7172	2.96E-02
miR-208b-3p	miR-548ad-3p	-0.0318	9.26E-01
miR-219b-3p	miR-548ad-3p	0.9311	3.13E-05
miR-25-5p	miR-548ad-3p	0.8114	2.43E-03
miR-378g	miR-548ad-3p	0.7186	1.27E-02
miR-382-3p	miR-548ad-3p	0.9502	7.46E-06
miR-432-5p	miR-548ad-3p	0.9011	1.52E-04
miR-4455	miR-548ad-3p	0.7712	5.45E-03
miR-501-3p	miR-548ad-3p	0.9081	1.11E-04

miR-506-5p	miR-548ad-3p	0.9070	1.17E-04
miR-508-3p	miR-548ad-3p	0.9576	3.65E-06
miR-516b-5p	miR-548ad-3p	0.7212	1.86E-02
miR-517a-3p	miR-548ad-3p	0.8412	2.28E-03
miR-519c-3p	miR-548ad-3p	0.9237	4.90E-05
miR-526a+518c-5p+518d-5p	miR-548ad-3p	0.7605	1.07E-02
miR-1245b-5p	miR-548ah-5p	0.9371	2.10E-05
miR-127-3p	miR-548ah-5p	0.9261	4.27E-05
miR-1271-5p	miR-548ah-5p	0.9387	1.87E-05
miR-186-5p	miR-548ah-5p	0.8138	7.60E-03
miR-196a-5p	miR-548ah-5p	0.7289	2.59E-02
miR-208b-3p	miR-548ah-5p	-0.0115	9.73E-01
miR-219b-3p	miR-548ah-5p	0.9393	1.79E-05
miR-25-5p	miR-548ah-5p	0.8358	1.35E-03
miR-378g	miR-548ah-5p	0.7410	9.08E-03
miR-382-3p	miR-548ah-5p	0.9481	8.95E-06
miR-432-5p	miR-548ah-5p	0.9158	7.55E-05
miR-4455	miR-548ah-5p	0.7218	1.22E-02
miR-501-3p	miR-548ah-5p	0.9370	2.11E-05
miR-506-5p	miR-548ah-5p	0.8365	1.33E-03
miR-508-3p	miR-548ah-5p	0.9763	2.74E-07
miR-516b-5p	miR-548ah-5p	0.8068	4.79E-03
miR-517a-3p	miR-548ah-5p	0.8383	2.45E-03
miR-519c-3p	miR-548ah-5p	0.9723	5.49E-07
miR-526a+518c-5p+518d-5p	miR-548ah-5p	0.7613	1.05E-02
miR-548ad-3p	miR-548ah-5p	0.9561	4.26E-06
miR-1245b-5p	miR-548v	0.8197	2.01E-03
miR-127-3p	miR-548v	0.7379	9.53E-03
miR-1271-5p	miR-548v	0.8520	8.68E-04
miR-186-5p	miR-548v	0.7986	9.85E-03
miR-196a-5p	miR-548v	0.5754	1.05E-01
miR-208b-3p	miR-548v	0.3240	3.31E-01
miR-219b-3p	miR-548v	0.7983	3.22E-03
miR-25-5p	miR-548v	0.7833	4.34E-03
miR-378g	miR-548v	0.7068	1.50E-02
miR-382-3p	miR-548v	0.8315	1.51E-03
miR-432-5p	miR-548v	0.8438	1.09E-03
miR-4455	miR-548v	0.8952	1.96E-04
miR-501-3p	miR-548v	0.8492	9.41E-04
miR-506-5p	miR-548v	0.8706	4.89E-04
miR-508-3p	miR-548v	0.8186	2.06E-03
miR-516b-5p	miR-548v	0.6909	2.69E-02
miR-517a-3p	miR-548v	0.8105	4.45E-03
miR-519c-3p	miR-548v	0.8500	9.21E-04
miR-526a+518c-5p+518d-5p	miR-548v	0.5937	7.04E-02
miR-548ad-3p	miR-548v	0.8800	3.53E-04
miR-548ah-5p	miR-548v	0.8597	6.91E-04

miR-1245b-5p	miR-549a	0.8417	1.16E-03
miR-127-3p	miR-549a	0.9000	1.60E-04
miR-1271-5p	miR-549a	0.9331	2.75E-05
miR-186-5p	miR-549a	0.7244	2.73E-02
miR-196a-5p	miR-549a	0.7693	1.54E-02
miR-208b-3p	miR-549a	-0.1715	6.14E-01
miR-219b-3p	miR-549a	0.8857	2.86E-04
miR-25-5p	miR-549a	0.8602	6.81E-04
miR-378g	miR-549a	0.9276	3.89E-05
miR-382-3p	miR-549a	0.8774	3.87E-04
miR-432-5p	miR-549a	0.8778	3.82E-04
miR-4455	miR-549a	0.7403	9.17E-03
miR-501-3p	miR-549a	0.6282	3.85E-02
miR-506-5p	miR-549a	0.9172	7.01E-05
miR-508-3p	miR-549a	0.8410	1.18E-03
miR-516b-5p	miR-549a	0.8471	1.98E-03
miR-517a-3p	miR-549a	0.9269	1.14E-04
miR-519c-3p	miR-549a	0.7410	9.08E-03
miR-526a+518c-5p+518d-5p	miR-549a	0.9069	2.94E-04
miR-548ad-3p	miR-549a	0.8411	1.18E-03
miR-548ah-5p	miR-549a	0.8113	2.44E-03
miR-548v	miR-549a	0.7709	5.48E-03
miR-1245b-5p	miR-575	0.5573	9.42E-02
miR-127-3p	miR-575	0.8223	3.50E-03
miR-1271-5p	miR-575	0.6486	4.25E-02
miR-186-5p	miR-575	0.3062	4.23E-01
miR-196a-5p	miR-575	0.7726	1.47E-02
miR-208b-3p	miR-575	0.0111	9.76E-01
miR-219b-3p	miR-575	0.7683	9.44E-03
miR-25-5p	miR-575	0.7862	7.00E-03
miR-378g	miR-575	0.6237	5.40E-02
miR-382-3p	miR-575	0.6508	4.16E-02
miR-432-5p	miR-575	0.6027	6.52E-02
miR-4455	miR-575	0.6385	4.69E-02
miR-501-3p	miR-575	0.5918	7.15E-02
miR-506-5p	miR-575	0.7608	1.06E-02
miR-508-3p	miR-575	0.6823	2.97E-02
miR-516b-5p	miR-575	0.8644	1.25E-03
miR-517a-3p	miR-575	0.6584	3.84E-02
miR-519c-3p	miR-575	0.5327	1.13E-01
miR-526a+518c-5p+518d-5p	miR-575	0.7421	1.40E-02
miR-548ad-3p	miR-575	0.6154	5.82E-02
miR-548ah-5p	miR-575	0.6218	5.49E-02
miR-548v	miR-575	0.6455	4.38E-02
miR-549a	miR-575	0.7448	1.35E-02
miR-1245b-5p	miR-593-3p	0.8319	1.49E-03
miR-127-3p	miR-593-3p	0.8482	9.68E-04

miR-1271-5p	miR-593-3p	0.9475	9.47E-06
miR-186-5p	miR-593-3p	0.8929	1.19E-03
miR-196a-5p	miR-593-3p	0.5775	1.03E-01
miR-208b-3p	miR-593-3p	-0.0835	8.07E-01
miR-219b-3p	miR-593-3p	0.9152	7.80E-05
miR-25-5p	miR-593-3p	0.7417	8.98E-03
miR-378g	miR-593-3p	0.8088	2.58E-03
miR-382-3p	miR-593-3p	0.9582	3.45E-06
miR-432-5p	miR-593-3p	0.9679	1.07E-06
miR-4455	miR-593-3p	0.7113	1.41E-02
miR-501-3p	miR-593-3p	0.8333	1.44E-03
miR-506-5p	miR-593-3p	0.8610	6.64E-04
miR-508-3p	miR-593-3p	0.8649	5.89E-04
miR-516b-5p	miR-593-3p	0.6728	3.30E-02
miR-517a-3p	miR-593-3p	0.8491	1.88E-03
miR-519c-3p	miR-593-3p	0.8981	1.74E-04
miR-526a+518c-5p+518d-5p	miR-593-3p	0.7648	9.97E-03
miR-548ad-3p	miR-593-3p	0.9304	3.27E-05
miR-548ah-5p	miR-593-3p	0.9239	4.84E-05
miR-548v	miR-593-3p	0.8787	3.70E-04
miR-549a	miR-593-3p	0.8426	1.13E-03
miR-575	miR-593-3p	0.5073	1.35E-01
miR-1245b-5p	miR-612	0.7997	3.13E-03
miR-127-3p	miR-612	0.7108	1.42E-02
miR-1271-5p	miR-612	0.7488	8.01E-03
miR-186-5p	miR-612	0.5635	1.14E-01
miR-196a-5p	miR-612	0.5788	1.02E-01
miR-208b-3p	miR-612	0.4163	2.03E-01
miR-219b-3p	miR-612	0.7671	5.87E-03
miR-25-5p	miR-612	0.7282	1.11E-02
miR-378g	miR-612	0.5289	9.44E-02
miR-382-3p	miR-612	0.7612	6.50E-03
miR-432-5p	miR-612	0.7154	1.33E-02
miR-4455	miR-612	0.8503	9.13E-04
miR-501-3p	miR-612	0.9199	6.08E-05
miR-506-5p	miR-612	0.7847	4.24E-03
miR-508-3p	miR-612	0.8208	1.96E-03
miR-516b-5p	miR-612	0.5927	7.10E-02
miR-517a-3p	miR-612	0.6650	3.59E-02
miR-519c-3p	miR-612	0.8301	1.56E-03
miR-526a+518c-5p+518d-5p	miR-612	0.3867	2.70E-01
miR-548ad-3p	miR-612	0.8398	1.22E-03
miR-548ah-5p	miR-612	0.8483	9.65E-04
miR-548v	miR-612	0.9366	2.17E-05
miR-549a	miR-612	0.6232	4.05E-02
miR-575	miR-612	0.6455	4.38E-02
miR-593-3p	miR-612	0.7629	6.31E-03

miR-1245b-5p	miR-627-3p	0.8822	3.26E-04
miR-127-3p	miR-627-3p	0.8636	6.13E-04
miR-1271-5p	miR-627-3p	0.8239	1.82E-03
miR-186-5p	miR-627-3p	0.4951	1.75E-01
miR-196a-5p	miR-627-3p	0.7896	1.14E-02
miR-208b-3p	miR-627-3p	0.1109	7.45E-01
miR-219b-3p	miR-627-3p	0.8812	3.37E-04
miR-25-5p	miR-627-3p	0.8372	1.31E-03
miR-378g	miR-627-3p	0.5898	5.61E-02
miR-382-3p	miR-627-3p	0.8668	5.54E-04
miR-432-5p	miR-627-3p	0.7685	5.72E-03
miR-4455	miR-627-3p	0.7792	4.70E-03
miR-501-3p	miR-627-3p	0.9587	3.26E-06
miR-506-5p	miR-627-3p	0.8355	1.36E-03
miR-508-3p	miR-627-3p	0.9494	8.06E-06
miR-516b-5p	miR-627-3p	0.7860	7.02E-03
miR-517a-3p	miR-627-3p	0.7166	1.97E-02
miR-519c-3p	miR-627-3p	0.9042	1.32E-04
miR-526a+518c-5p+518d-5p	miR-627-3p	0.6269	5.24E-02
miR-548ad-3p	miR-627-3p	0.9220	5.39E-05
miR-548ah-5p	miR-627-3p	0.9292	3.52E-05
miR-548v	miR-627-3p	0.8368	1.32E-03
miR-549a	miR-627-3p	0.7118	1.40E-02
miR-575	miR-627-3p	0.7551	1.16E-02
miR-593-3p	miR-627-3p	0.7839	4.30E-03
miR-612	miR-627-3p	0.9138	8.39E-05
miR-1245b-5p	miR-744-5p	0.6581	3.86E-02
miR-127-3p	miR-744-5p	0.6317	5.01E-02
miR-1271-5p	miR-744-5p	0.7820	7.53E-03
miR-186-5p	miR-744-5p	0.8794	1.78E-03
miR-196a-5p	miR-744-5p	0.3617	3.39E-01
miR-208b-3p	miR-744-5p	0.3794	2.80E-01
miR-219b-3p	miR-744-5p	0.7260	1.75E-02
miR-25-5p	miR-744-5p	0.5756	8.17E-02
miR-378g	miR-744-5p	0.5795	7.91E-02
miR-382-3p	miR-744-5p	0.7226	1.82E-02
miR-432-5p	miR-744-5p	0.7529	1.19E-02
miR-4455	miR-744-5p	0.7238	1.80E-02
miR-501-3p	miR-744-5p	0.8518	1.76E-03
miR-506-5p	miR-744-5p	0.7315	1.62E-02
miR-508-3p	miR-744-5p	0.7224	1.83E-02
miR-516b-5p	miR-744-5p	0.5200	1.23E-01
miR-517a-3p	miR-744-5p	0.7567	1.13E-02
miR-519c-3p	miR-744-5p	0.7623	1.04E-02
miR-526a+518c-5p+518d-5p	miR-744-5p	0.4779	1.62E-01
miR-548ad-3p	miR-744-5p	0.8331	2.76E-03
miR-548ah-5p	miR-744-5p	0.8193	3.73E-03

miR-548v	miR-744-5p	0.9233	1.38E-04
miR-549a	miR-744-5p	0.6841	2.91E-02
miR-575	miR-744-5p	0.5931	7.08E-02
miR-593-3p	miR-744-5p	0.8233	3.43E-03
miR-612	miR-744-5p	0.8663	1.19E-03
miR-627-3p	miR-744-5p	0.7403	1.43E-02
miR-1245b-5p	miR-765	0.8367	2.54E-03
miR-127-3p	miR-765	0.8771	8.57E-04
miR-1271-5p	miR-765	0.8954	4.61E-04
miR-186-5p	miR-765	0.8997	9.53E-04
miR-196a-5p	miR-765	0.4507	2.23E-01
miR-208b-3p	miR-765	-0.0415	9.09E-01
miR-219b-3p	miR-765	0.8969	4.36E-04
miR-25-5p	miR-765	0.8290	3.02E-03
miR-378g	miR-765	0.9152	2.04E-04
miR-382-3p	miR-765	0.9469	3.27E-05
miR-432-5p	miR-765	0.8947	4.73E-04
miR-4455	miR-765	0.7085	2.18E-02
miR-501-3p	miR-765	0.7236	1.80E-02
miR-506-5p	miR-765	0.8490	1.89E-03
miR-508-3p	miR-765	0.8566	1.55E-03
miR-516b-5p	miR-765	0.7462	1.32E-02
miR-517a-3p	miR-765	0.9023	3.53E-04
miR-519c-3p	miR-765	0.8647	1.24E-03
miR-526a+518c-5p+518d-5p	miR-765	0.8151	4.06E-03
miR-548ad-3p	miR-765	0.8824	7.23E-04
miR-548ah-5p	miR-765	0.8965	4.42E-04
miR-548v	miR-765	0.8000	5.46E-03
miR-549a	miR-765	0.9157	1.99E-04
miR-575	miR-765	0.5938	7.03E-02
miR-593-3p	miR-765	0.9146	2.10E-04
miR-612	miR-765	0.5888	7.33E-02
miR-627-3p	miR-765	0.6973	2.50E-02
miR-744-5p	miR-765	0.7434	1.37E-02

Supplementary Table 6: Intra-correlation and clustering of tumor-exclusive exosomal miRNAs in saliva

Row	Column	Correlation Coefficient	p-value
miR-1245b-5p	miR-127-3p	0.5572	7.50E-02
miR-1245b-5p	miR-1271-5p	0.8766	3.98E-04
miR-127-3p	miR-1271-5p	0.3856	2.42E-01
miR-1245b-5p	miR-186-5p	0.4351	1.81E-01
miR-127-3p	miR-186-5p	0.5382	8.77E-02
miR-1271-5p	miR-186-5p	0.3468	2.96E-01
miR-1245b-5p	miR-196a-5p	0.3530	2.87E-01
miR-127-3p	miR-196a-5p	0.6715	2.37E-02
miR-1271-5p	miR-196a-5p	0.1896	5.77E-01
miR-186-5p	miR-196a-5p	0.3194	3.38E-01
miR-1245b-5p	miR-208b-3p	0.3999	2.23E-01
miR-127-3p	miR-208b-3p	0.0906	7.91E-01
miR-1271-5p	miR-208b-3p	0.7191	1.26E-02
miR-186-5p	miR-208b-3p	0.1702	6.17E-01
miR-196a-5p	miR-208b-3p	0.1329	6.97E-01
miR-1245b-5p	miR-219b-3p	0.9402	1.68E-05
miR-127-3p	miR-219b-3p	0.3949	2.29E-01
miR-1271-5p	miR-219b-3p	0.8364	1.33E-03
miR-186-5p	miR-219b-3p	0.5132	1.06E-01
miR-196a-5p	miR-219b-3p	0.3422	3.03E-01
miR-208b-3p	miR-219b-3p	0.4281	1.89E-01
miR-1245b-5p	miR-25-5p	0.7620	6.41E-03
miR-127-3p	miR-25-5p	0.5031	1.15E-01
miR-1271-5p	miR-25-5p	0.7050	1.54E-02
miR-186-5p	miR-25-5p	0.5011	1.16E-01
miR-196a-5p	miR-25-5p	0.1007	7.68E-01
miR-208b-3p	miR-25-5p	0.2717	4.19E-01
miR-219b-3p	miR-25-5p	0.6281	3.85E-02
miR-1245b-5p	miR-378g	0.9292	3.52E-05
miR-127-3p	miR-378g	0.6994	1.66E-02
miR-1271-5p	miR-378g	0.7564	7.06E-03
miR-186-5p	miR-378g	0.5517	7.85E-02
miR-196a-5p	miR-378g	0.4381	1.78E-01
miR-208b-3p	miR-378g	0.3531	2.87E-01
miR-219b-3p	miR-378g	0.8337	1.43E-03
miR-25-5p	miR-378g	0.8233	1.84E-03
miR-1245b-5p	miR-382-3p	0.7731	5.26E-03
miR-127-3p	miR-382-3p	0.5786	6.22E-02
miR-1271-5p	miR-382-3p	0.8589	7.09E-04
miR-186-5p	miR-382-3p	0.4039	2.18E-01
miR-196a-5p	miR-382-3p	0.4955	1.21E-01
miR-208b-3p	miR-382-3p	0.5640	7.07E-02
miR-219b-3p	miR-382-3p	0.7526	7.52E-03

miR-25-5p	miR-382-3p	0.5384	8.75E-02
miR-378g	miR-382-3p	0.6325	3.68E-02
miR-1245b-5p	miR-432-5p	0.8969	1.83E-04
miR-127-3p	miR-432-5p	0.4534	1.61E-01
miR-1271-5p	miR-432-5p	0.9078	1.12E-04
miR-186-5p	miR-432-5p	0.4887	1.27E-01
miR-196a-5p	miR-432-5p	0.4529	1.62E-01
miR-208b-3p	miR-432-5p	0.6390	3.43E-02
miR-219b-3p	miR-432-5p	0.9242	4.78E-05
miR-25-5p	miR-432-5p	0.5751	6.42E-02
miR-378g	miR-432-5p	0.8068	2.69E-03
miR-382-3p	miR-432-5p	0.8336	1.43E-03
miR-1245b-5p	miR-4455	0.7357	9.86E-03
miR-127-3p	miR-4455	0.2095	5.36E-01
miR-1271-5p	miR-4455	0.9094	1.04E-04
miR-186-5p	miR-4455	0.3138	3.47E-01
miR-196a-5p	miR-4455	0.2732	4.16E-01
miR-208b-3p	miR-4455	0.8286	1.62E-03
miR-219b-3p	miR-4455	0.7440	8.66E-03
miR-25-5p	miR-4455	0.6010	5.05E-02
miR-378g	miR-4455	0.6533	2.93E-02
miR-382-3p	miR-4455	0.7368	9.70E-03
miR-432-5p	miR-4455	0.8804	3.47E-04
miR-1245b-5p	miR-501-3p	0.3973	2.26E-01
miR-127-3p	miR-501-3p	0.2443	4.69E-01
miR-1271-5p	miR-501-3p	0.4295	1.87E-01
miR-186-5p	miR-501-3p	0.3336	3.16E-01
miR-196a-5p	miR-501-3p	0.6193	4.22E-02
miR-208b-3p	miR-501-3p	0.5354	8.96E-02
miR-219b-3p	miR-501-3p	0.5517	7.85E-02
miR-25-5p	miR-501-3p	0.2096	5.36E-01
miR-378g	miR-501-3p	0.4443	1.71E-01
miR-382-3p	miR-501-3p	0.4863	1.29E-01
miR-432-5p	miR-501-3p	0.6038	4.92E-02
miR-4455	miR-501-3p	0.5829	5.98E-02
miR-1245b-5p	miR-506-5p	0.8829	3.17E-04
miR-127-3p	miR-506-5p	0.6157	4.37E-02
miR-1271-5p	miR-506-5p	0.9194	6.25E-05
miR-186-5p	miR-506-5p	0.4982	1.19E-01
miR-196a-5p	miR-506-5p	0.4743	1.41E-01
miR-208b-3p	miR-506-5p	0.5586	7.41E-02
miR-219b-3p	miR-506-5p	0.8170	2.14E-03
miR-25-5p	miR-506-5p	0.7224	1.20E-02
miR-378g	miR-506-5p	0.8087	2.58E-03
miR-382-3p	miR-506-5p	0.9215	5.55E-05
miR-432-5p	miR-506-5p	0.9149	7.89E-05
miR-4455	miR-506-5p	0.8424	1.14E-03

miR-501-3p	miR-506-5p	0.4464	1.69E-01
miR-1245b-5p	miR-508-3p	0.8349	1.38E-03
miR-127-3p	miR-508-3p	0.5857	5.83E-02
miR-1271-5p	miR-508-3p	0.8874	2.68E-04
miR-186-5p	miR-508-3p	0.6207	4.16E-02
miR-196a-5p	miR-508-3p	0.4911	1.25E-01
miR-208b-3p	miR-508-3p	0.7002	1.64E-02
miR-219b-3p	miR-508-3p	0.8392	1.24E-03
miR-25-5p	miR-508-3p	0.6410	3.36E-02
miR-378g	miR-508-3p	0.8228	1.87E-03
miR-382-3p	miR-508-3p	0.8419	1.15E-03
miR-432-5p	miR-508-3p	0.9329	2.78E-05
miR-4455	miR-508-3p	0.8684	5.24E-04
miR-501-3p	miR-508-3p	0.6279	3.86E-02
miR-506-5p	miR-508-3p	0.9219	5.44E-05
miR-1245b-5p	miR-516b-5p	0.0836	8.07E-01
miR-127-3p	miR-516b-5p	0.3357	3.13E-01
miR-1271-5p	miR-516b-5p	-0.1404	6.81E-01
miR-186-5p	miR-516b-5p	0.4948	1.22E-01
miR-196a-5p	miR-516b-5p	0.7146	1.35E-02
miR-208b-3p	miR-516b-5p	-0.0775	8.21E-01
miR-219b-3p	miR-516b-5p	0.1802	5.96E-01
miR-25-5p	miR-516b-5p	-0.1289	7.06E-01
miR-378g	miR-516b-5p	0.1910	5.74E-01
miR-382-3p	miR-516b-5p	0.0758	8.25E-01
miR-432-5p	miR-516b-5p	0.2035	5.48E-01
miR-4455	miR-516b-5p	0.0368	9.15E-01
miR-501-3p	miR-516b-5p	0.3459	2.97E-01
miR-506-5p	miR-516b-5p	0.1341	6.94E-01
miR-508-3p	miR-516b-5p	0.2692	4.23E-01
miR-1245b-5p	miR-517a-3p	0.9200	1.63E-04
miR-127-3p	miR-517a-3p	0.6083	6.20E-02
miR-1271-5p	miR-517a-3p	0.8368	2.54E-03
miR-186-5p	miR-517a-3p	0.3675	2.96E-01
miR-196a-5p	miR-517a-3p	0.1873	6.04E-01
miR-208b-3p	miR-517a-3p	0.0157	9.66E-01
miR-219b-3p	miR-517a-3p	0.7269	1.72E-02
miR-25-5p	miR-517a-3p	0.9291	1.01E-04
miR-378g	miR-517a-3p	0.8599	1.42E-03
miR-382-3p	miR-517a-3p	0.6985	2.47E-02
miR-432-5p	miR-517a-3p	0.7251	1.76E-02
miR-4455	miR-517a-3p	0.5180	1.25E-01
miR-501-3p	miR-517a-3p	0.0738	8.39E-01
miR-506-5p	miR-517a-3p	0.8511	1.79E-03
miR-508-3p	miR-517a-3p	0.7137	2.05E-02
miR-516b-5p	miR-517a-3p	-0.1855	6.08E-01
miR-1245b-5p	miR-519c-3p	0.8147	2.26E-03

miR-127-3p	miR-519c-3p	0.3726	2.59E-01
miR-1271-5p	miR-519c-3p	0.8689	5.16E-04
miR-186-5p	miR-519c-3p	0.3519	2.88E-01
miR-196a-5p	miR-519c-3p	0.3912	2.34E-01
miR-208b-3p	miR-519c-3p	0.7122	1.39E-02
miR-219b-3p	miR-519c-3p	0.7862	4.11E-03
miR-25-5p	miR-519c-3p	0.7175	1.29E-02
miR-378g	miR-519c-3p	0.7628	6.32E-03
miR-382-3p	miR-519c-3p	0.7785	4.77E-03
miR-432-5p	miR-519c-3p	0.8423	1.14E-03
miR-4455	miR-519c-3p	0.9051	1.27E-04
miR-501-3p	miR-519c-3p	0.6017	5.02E-02
miR-506-5p	miR-519c-3p	0.8371	1.31E-03
miR-508-3p	miR-519c-3p	0.8334	1.44E-03
miR-516b-5p	miR-519c-3p	0.0743	8.28E-01
miR-517a-3p	miR-519c-3p	0.6740	3.26E-02
miR-1245b-5p	miR-526a+518c-5p+518d-5p	0.8270	1.69E-03
miR-127-3p	miR-526a+518c-5p+518d-5p	0.6510	3.01E-02
miR-1271-5p	miR-526a+518c-5p+518d-5p	0.6123	4.52E-02
miR-186-5p	miR-526a+518c-5p+518d-5p	0.2846	3.96E-01
miR-196a-5p	miR-526a+518c-5p+518d-5p	0.5492	8.02E-02
miR-208b-3p	miR-526a+518c-5p+518d-5p	0.1633	6.31E-01
miR-219b-3p	miR-526a+518c-5p+518d-5p	0.7965	3.35E-03
miR-25-5p	miR-526a+518c-5p+518d-5p	0.3793	2.50E-01
miR-378g	miR-526a+518c-5p+518d-5p	0.7497	7.89E-03
miR-382-3p	miR-526a+518c-5p+518d-5p	0.6944	1.77E-02
miR-432-5p	miR-526a+518c-5p+518d-5p	0.7256	1.15E-02
miR-4455	miR-526a+518c-5p+518d-5p	0.4089	2.12E-01
miR-501-3p	miR-526a+518c-5p+518d-5p	0.4015	2.21E-01
miR-506-5p	miR-526a+518c-5p+518d-5p	0.6826	2.06E-02
miR-508-3p	miR-526a+518c-5p+518d-5p	0.6672	2.49E-02
miR-516b-5p	miR-526a+518c-5p+518d-5p	0.2478	4.63E-01
miR-517a-3p	miR-526a+518c-5p+518d-5p	0.5373	1.09E-01
miR-519c-3p	miR-526a+518c-5p+518d-5p	0.5024	1.15E-01
miR-1245b-5p	miR-548ad-3p	0.8193	3.73E-03
miR-127-3p	miR-548ad-3p	0.4773	1.63E-01
miR-1271-5p	miR-548ad-3p	0.8146	4.10E-03
miR-186-5p	miR-548ad-3p	0.2170	5.47E-01
miR-196a-5p	miR-548ad-3p	0.1021	7.79E-01
miR-208b-3p	miR-548ad-3p	0.2324	5.18E-01
miR-219b-3p	miR-548ad-3p	0.5537	9.68E-02
miR-25-5p	miR-548ad-3p	0.9136	2.20E-04
miR-378g	miR-548ad-3p	0.8249	3.31E-03
miR-382-3p	miR-548ad-3p	0.5170	1.26E-01
miR-432-5p	miR-548ad-3p	0.6000	6.67E-02
miR-4455	miR-548ad-3p	0.6382	4.71E-02
miR-501-3p	miR-548ad-3p	0.0174	9.62E-01

miR-506-5p	miR-548ad-3p	0.7608	1.06E-02
miR-508-3p	miR-548ad-3p	0.7096	2.15E-02
miR-516b-5p	miR-548ad-3p	-0.1544	6.70E-01
miR-517a-3p	miR-548ad-3p	0.8790	8.07E-04
miR-519c-3p	miR-548ad-3p	0.7155	2.00E-02
miR-526a+518c-5p+518d-5p	miR-548ad-3p	0.3548	3.14E-01
miR-1245b-5p	miR-548ah-5p	0.8410	1.18E-03
miR-127-3p	miR-548ah-5p	0.6745	2.28E-02
miR-1271-5p	miR-548ah-5p	0.8949	1.99E-04
miR-186-5p	miR-548ah-5p	0.3758	2.55E-01
miR-196a-5p	miR-548ah-5p	0.4778	1.37E-01
miR-208b-3p	miR-548ah-5p	0.6679	2.47E-02
miR-219b-3p	miR-548ah-5p	0.7301	1.07E-02
miR-25-5p	miR-548ah-5p	0.6004	5.08E-02
miR-378g	miR-548ah-5p	0.7987	3.20E-03
miR-382-3p	miR-548ah-5p	0.8657	5.74E-04
miR-432-5p	miR-548ah-5p	0.8819	3.29E-04
miR-4455	miR-548ah-5p	0.7999	3.12E-03
miR-501-3p	miR-548ah-5p	0.4072	2.14E-01
miR-506-5p	miR-548ah-5p	0.9302	3.32E-05
miR-508-3p	miR-548ah-5p	0.9006	1.56E-04
miR-516b-5p	miR-548ah-5p	0.0908	7.91E-01
miR-517a-3p	miR-548ah-5p	0.7768	8.21E-03
miR-519c-3p	miR-548ah-5p	0.7960	3.38E-03
miR-526a+518c-5p+518d-5p	miR-548ah-5p	0.7112	1.41E-02
miR-548ad-3p	miR-548ah-5p	0.7650	9.94E-03
miR-1245b-5p	miR-548v	0.5467	8.18E-02
miR-127-3p	miR-548v	0.7746	5.12E-03
miR-1271-5p	miR-548v	0.3411	3.05E-01
miR-186-5p	miR-548v	0.4090	2.12E-01
miR-196a-5p	miR-548v	0.5703	6.69E-02
miR-208b-3p	miR-548v	0.1291	7.05E-01
miR-219b-3p	miR-548v	0.4209	1.97E-01
miR-25-5p	miR-548v	0.5210	1.00E-01
miR-378g	miR-548v	0.6464	3.16E-02
miR-382-3p	miR-548v	0.5086	1.10E-01
miR-432-5p	miR-548v	0.3452	2.99E-01
miR-4455	miR-548v	0.2208	5.14E-01
miR-501-3p	miR-548v	0.2674	4.27E-01
miR-506-5p	miR-548v	0.4825	1.33E-01
miR-508-3p	miR-548v	0.5020	1.16E-01
miR-516b-5p	miR-548v	0.3654	2.69E-01
miR-517a-3p	miR-548v	0.5375	1.09E-01
miR-519c-3p	miR-548v	0.5230	9.88E-02
miR-526a+518c-5p+518d-5p	miR-548v	0.5869	5.77E-02
miR-548ad-3p	miR-548v	0.5543	9.64E-02
miR-548ah-5p	miR-548v	0.5213	1.00E-01

miR-1245b-5p	miR-549a	0.6824	2.07E-02
miR-127-3p	miR-549a	0.5003	1.17E-01
miR-1271-5p	miR-549a	0.5871	5.76E-02
miR-186-5p	miR-549a	0.1819	5.92E-01
miR-196a-5p	miR-549a	0.5047	1.13E-01
miR-208b-3p	miR-549a	0.2161	5.23E-01
miR-219b-3p	miR-549a	0.5468	8.17E-02
miR-25-5p	miR-549a	0.6787	2.17E-02
miR-378g	miR-549a	0.6211	4.14E-02
miR-382-3p	miR-549a	0.6947	1.77E-02
miR-432-5p	miR-549a	0.5402	8.63E-02
miR-4455	miR-549a	0.5523	7.81E-02
miR-501-3p	miR-549a	0.2643	4.32E-01
miR-506-5p	miR-549a	0.7220	1.21E-02
miR-508-3p	miR-549a	0.5335	9.10E-02
miR-516b-5p	miR-549a	0.1455	6.70E-01
miR-517a-3p	miR-549a	0.7626	1.03E-02
miR-519c-3p	miR-549a	0.7694	5.63E-03
miR-526a+518c-5p+518d-5p	miR-549a	0.4924	1.24E-01
miR-548ad-3p	miR-549a	0.7409	1.42E-02
miR-548ah-5p	miR-549a	0.6107	4.59E-02
miR-548v	miR-549a	0.6858	1.98E-02
miR-1245b-5p	miR-575	0.9338	7.76E-05
miR-127-3p	miR-575	0.2526	4.81E-01
miR-1271-5p	miR-575	0.7025	2.35E-02
miR-186-5p	miR-575	-0.0292	9.36E-01
miR-196a-5p	miR-575	0.1201	7.41E-01
miR-208b-3p	miR-575	-0.1580	6.63E-01
miR-219b-3p	miR-575	0.8075	4.73E-03
miR-25-5p	miR-575	0.7256	1.75E-02
miR-378g	miR-575	0.7229	1.82E-02
miR-382-3p	miR-575	0.5143	1.28E-01
miR-432-5p	miR-575	0.6566	3.92E-02
miR-4455	miR-575	0.4646	1.76E-01
miR-501-3p	miR-575	0.0275	9.40E-01
miR-506-5p	miR-575	0.6466	4.33E-02
miR-508-3p	miR-575	0.4076	2.42E-01
miR-516b-5p	miR-575	-0.1817	6.15E-01
miR-517a-3p	miR-575	0.8024	5.22E-03
miR-519c-3p	miR-575	0.6598	3.79E-02
miR-526a+518c-5p+518d-5p	miR-575	0.6199	5.59E-02
miR-548ad-3p	miR-575	0.7360	1.52E-02
miR-548ah-5p	miR-575	0.5273	1.17E-01
miR-548v	miR-575	0.4112	2.38E-01
miR-549a	miR-575	0.8092	4.57E-03
miR-1245b-5p	miR-593-3p	0.8310	1.53E-03
miR-127-3p	miR-593-3p	0.4830	1.32E-01

miR-1271-5p	miR-593-3p	0.8306	1.55E-03
miR-186-5p	miR-593-3p	0.4224	1.96E-01
miR-196a-5p	miR-593-3p	0.2727	4.17E-01
miR-208b-3p	miR-593-3p	0.4092	2.11E-01
miR-219b-3p	miR-593-3p	0.7130	1.38E-02
miR-25-5p	miR-593-3p	0.9221	5.36E-05
miR-378g	miR-593-3p	0.8084	2.60E-03
miR-382-3p	miR-593-3p	0.7224	1.20E-02
miR-432-5p	miR-593-3p	0.7361	9.80E-03
miR-4455	miR-593-3p	0.7849	4.21E-03
miR-501-3p	miR-593-3p	0.3057	3.61E-01
miR-506-5p	miR-593-3p	0.8852	2.91E-04
miR-508-3p	miR-593-3p	0.7642	6.17E-03
miR-516b-5p	miR-593-3p	-0.0113	9.74E-01
miR-517a-3p	miR-593-3p	0.9104	2.52E-04
miR-519c-3p	miR-593-3p	0.8349	1.38E-03
miR-526a+518c-5p+518d-5p	miR-593-3p	0.4748	1.40E-01
miR-548ad-3p	miR-593-3p	0.9186	1.74E-04
miR-548ah-5p	miR-593-3p	0.7479	8.12E-03
miR-548v	miR-593-3p	0.4856	1.30E-01
miR-549a	miR-593-3p	0.8142	2.28E-03
miR-575	miR-593-3p	0.7820	7.52E-03
miR-1245b-5p	miR-612	0.3546	3.15E-01
miR-127-3p	miR-612	-0.0343	9.25E-01
miR-1271-5p	miR-612	0.7147	2.02E-02
miR-186-5p	miR-612	0.0188	9.59E-01
miR-196a-5p	miR-612	-0.0601	8.69E-01
miR-208b-3p	miR-612	0.7982	5.65E-03
miR-219b-3p	miR-612	0.2911	4.14E-01
miR-25-5p	miR-612	0.6129	5.96E-02
miR-378g	miR-612	0.3534	3.16E-01
miR-382-3p	miR-612	0.3951	2.58E-01
miR-432-5p	miR-612	0.4228	2.24E-01
miR-4455	miR-612	0.8049	4.97E-03
miR-501-3p	miR-612	0.3824	2.75E-01
miR-506-5p	miR-612	0.4536	1.88E-01
miR-508-3p	miR-612	0.5512	9.87E-02
miR-516b-5p	miR-612	-0.3131	3.78E-01
miR-517a-3p	miR-612	0.4567	1.85E-01
miR-519c-3p	miR-612	0.8264	3.20E-03
miR-526a+518c-5p+518d-5p	miR-612	-0.2130	5.55E-01
miR-548ad-3p	miR-612	0.6412	4.57E-02
miR-548ah-5p	miR-612	0.4413	2.02E-01
miR-548v	miR-612	0.2585	4.71E-01
miR-549a	miR-612	0.5173	1.26E-01
miR-575	miR-612	0.3672	2.97E-01
miR-593-3p	miR-612	0.6356	4.83E-02

miR-1245b-5p	miR-627-3p	0.6781	2.18E-02
miR-127-3p	miR-627-3p	0.8262	1.72E-03
miR-1271-5p	miR-627-3p	0.6369	3.51E-02
miR-186-5p	miR-627-3p	0.6345	3.60E-02
miR-196a-5p	miR-627-3p	0.6170	4.32E-02
miR-208b-3p	miR-627-3p	0.5620	7.19E-02
miR-219b-3p	miR-627-3p	0.5856	5.84E-02
miR-25-5p	miR-627-3p	0.5561	7.56E-02
miR-378g	miR-627-3p	0.8069	2.68E-03
miR-382-3p	miR-627-3p	0.6551	2.87E-02
miR-432-5p	miR-627-3p	0.7222	1.21E-02
miR-4455	miR-627-3p	0.5977	5.21E-02
miR-501-3p	miR-627-3p	0.4818	1.33E-01
miR-506-5p	miR-627-3p	0.7481	8.10E-03
miR-508-3p	miR-627-3p	0.8426	1.13E-03
miR-516b-5p	miR-627-3p	0.3799	2.49E-01
miR-517a-3p	miR-627-3p	0.5296	1.15E-01
miR-519c-3p	miR-627-3p	0.6749	2.27E-02
miR-526a+518c-5p+518d-5p	miR-627-3p	0.5880	5.71E-02
miR-548ad-3p	miR-627-3p	0.5397	1.07E-01
miR-548ah-5p	miR-627-3p	0.8511	8.91E-04
miR-548v	miR-627-3p	0.6874	1.94E-02
miR-549a	miR-627-3p	0.4697	1.45E-01
miR-575	miR-627-3p	0.1309	7.18E-01
miR-593-3p	miR-627-3p	0.5840	5.92E-02
miR-612	miR-627-3p	0.2827	4.29E-01
miR-1245b-5p	miR-744-5p	0.7181	1.28E-02
miR-127-3p	miR-744-5p	0.4467	1.68E-01
miR-1271-5p	miR-744-5p	0.7851	4.20E-03
miR-186-5p	miR-744-5p	0.4995	1.18E-01
miR-196a-5p	miR-744-5p	0.3340	3.15E-01
miR-208b-3p	miR-744-5p	0.6071	4.76E-02
miR-219b-3p	miR-744-5p	0.6315	3.72E-02
miR-25-5p	miR-744-5p	0.8116	2.42E-03
miR-378g	miR-744-5p	0.7774	4.86E-03
miR-382-3p	miR-744-5p	0.6144	4.43E-02
miR-432-5p	miR-744-5p	0.7460	8.39E-03
miR-4455	miR-744-5p	0.8656	5.74E-04
miR-501-3p	miR-744-5p	0.4353	1.81E-01
miR-506-5p	miR-744-5p	0.8325	1.47E-03
miR-508-3p	miR-744-5p	0.8405	1.20E-03
miR-516b-5p	miR-744-5p	0.1692	6.19E-01
miR-517a-3p	miR-744-5p	0.6910	2.69E-02
miR-519c-3p	miR-744-5p	0.8232	1.85E-03
miR-526a+518c-5p+518d-5p	miR-744-5p	0.3489	2.93E-01
miR-548ad-3p	miR-744-5p	0.8413	2.28E-03
miR-548ah-5p	miR-744-5p	0.7576	6.92E-03

miR-548v	miR-744-5p	0.4194	1.99E-01
miR-549a	miR-744-5p	0.6339	3.62E-02
miR-575	miR-744-5p	0.4823	1.58E-01
miR-593-3p	miR-744-5p	0.9049	1.29E-04
miR-612	miR-744-5p	0.6904	2.71E-02
miR-627-3p	miR-744-5p	0.7074	1.49E-02
miR-1245b-5p	miR-765	0.8566	7.58E-04
miR-127-3p	miR-765	0.7792	4.70E-03
miR-1271-5p	miR-765	0.8042	2.85E-03
miR-186-5p	miR-765	0.5168	1.04E-01
miR-196a-5p	miR-765	0.5665	6.92E-02
miR-208b-3p	miR-765	0.4537	1.61E-01
miR-219b-3p	miR-765	0.7472	8.22E-03
miR-25-5p	miR-765	0.7799	4.64E-03
miR-378g	miR-765	0.8784	3.73E-04
miR-382-3p	miR-765	0.8463	1.02E-03
miR-432-5p	miR-765	0.8165	2.17E-03
miR-4455	miR-765	0.6925	1.82E-02
miR-501-3p	miR-765	0.4937	1.23E-01
miR-506-5p	miR-765	0.9088	1.07E-04
miR-508-3p	miR-765	0.8363	1.33E-03
miR-516b-5p	miR-765	0.1101	7.47E-01
miR-517a-3p	miR-765	0.8837	6.94E-04
miR-519c-3p	miR-765	0.8276	1.66E-03
miR-526a+518c-5p+518d-5p	miR-765	0.6824	2.07E-02
miR-548ad-3p	miR-765	0.7285	1.69E-02
miR-548ah-5p	miR-765	0.8836	3.09E-04
miR-548v	miR-765	0.6513	2.99E-02
miR-549a	miR-765	0.7561	7.09E-03
miR-575	miR-765	0.6343	4.88E-02
miR-593-3p	miR-765	0.8348	1.39E-03
miR-612	miR-765	0.4438	1.99E-01
miR-627-3p	miR-765	0.8356	1.36E-03
miR-744-5p	miR-765	0.7518	7.62E-03

Supplementary Table 7: Inter-correlation of tumor-exclusive exosomal miRNAs between plasma and saliva

Saliva	Plasma	Correlation Coefficient	p-value
miR-1245b-5p	miR-1245b-5p	0.2278	5.01E-01
miR-127-3p	miR-1245b-5p	0.1315	7.00E-01
miR-1271-5p	miR-1245b-5p	0.1910	5.74E-01
miR-186-5p	miR-1245b-5p	0.4253	1.92E-01
miR-196a-5p	miR-1245b-5p	-0.3335	3.16E-01
miR-208b-3p	miR-1245b-5p	0.1197	7.26E-01
miR-219b-3p	miR-1245b-5p	0.2650	4.31E-01
miR-25-5p	miR-1245b-5p	0.4001	2.23E-01
miR-378g	miR-1245b-5p	0.2819	4.01E-01
miR-382-3p	miR-1245b-5p	0.0838	8.06E-01
miR-432-5p	miR-1245b-5p	0.0382	9.11E-01
miR-4455	miR-1245b-5p	0.0096	9.78E-01
miR-501-3p	miR-1245b-5p	0.0234	9.46E-01
miR-506-5p	miR-1245b-5p	0.0271	9.37E-01
miR-508-3p	miR-1245b-5p	0.1872	5.82E-01
miR-516b-5p	miR-1245b-5p	-0.1918	5.72E-01
miR-517a-3p	miR-1245b-5p	0.2200	5.41E-01
miR-519c-3p	miR-1245b-5p	0.1880	5.80E-01
miR-526a+518c-5p+518d-5p	miR-1245b-5p	0.1088	7.50E-01
miR-548ad-3p	miR-1245b-5p	0.2137	5.53E-01
miR-548ah-5p	miR-1245b-5p	0.0300	9.30E-01
miR-548v	miR-1245b-5p	0.4306	1.86E-01
miR-549a	miR-1245b-5p	0.0015	9.97E-01
miR-575	miR-1245b-5p	0.0537	8.83E-01
miR-593-3p	miR-1245b-5p	0.1343	6.94E-01
miR-612	miR-1245b-5p	0.2774	4.38E-01
miR-627-3p	miR-1245b-5p	0.1956	5.64E-01
miR-744-5p	miR-1245b-5p	0.0790	8.18E-01
miR-765	miR-1245b-5p	0.1391	6.83E-01
miR-1245b-5p	miR-127-3p	0.0250	9.42E-01
miR-127-3p	miR-127-3p	0.1721	6.13E-01
miR-1271-5p	miR-127-3p	-0.1679	6.22E-01
miR-186-5p	miR-127-3p	0.3425	3.03E-01
miR-196a-5p	miR-127-3p	-0.2198	5.16E-01
miR-208b-3p	miR-127-3p	-0.2657	4.30E-01
miR-219b-3p	miR-127-3p	0.0336	9.22E-01
miR-25-5p	miR-127-3p	0.2597	4.41E-01
miR-378g	miR-127-3p	0.1570	6.45E-01
miR-382-3p	miR-127-3p	-0.1756	6.06E-01
miR-432-5p	miR-127-3p	-0.2503	4.58E-01
miR-4455	miR-127-3p	-0.3344	3.15E-01
miR-501-3p	miR-127-3p	-0.0975	7.76E-01
miR-506-5p	miR-127-3p	-0.2235	5.09E-01

miR-508-3p	miR-127-3p	-0.0899	7.93E-01
miR-516b-5p	miR-127-3p	-0.0171	9.60E-01
miR-517a-3p	miR-127-3p	0.1751	6.28E-01
miR-519c-3p	miR-127-3p	-0.0726	8.32E-01
miR-526a+518c-5p+518d-5p	miR-127-3p	0.0360	9.16E-01
miR-548ad-3p	miR-127-3p	0.1575	6.64E-01
miR-548ah-5p	miR-127-3p	-0.2350	4.87E-01
miR-548v	miR-127-3p	0.4937	1.23E-01
miR-549a	miR-127-3p	-0.0317	9.26E-01
miR-575	miR-127-3p	0.0628	8.63E-01
miR-593-3p	miR-127-3p	-0.0509	8.82E-01
miR-612	miR-127-3p	0.0774	8.32E-01
miR-627-3p	miR-127-3p	0.0521	8.79E-01
miR-744-5p	miR-127-3p	-0.1325	6.98E-01
miR-765	miR-127-3p	-0.0235	9.45E-01
miR-1245b-5p	miR-1271-5p	0.2447	4.68E-01
miR-127-3p	miR-1271-5p	0.2647	4.31E-01
miR-1271-5p	miR-1271-5p	0.0473	8.90E-01
miR-186-5p	miR-1271-5p	0.3863	2.41E-01
miR-196a-5p	miR-1271-5p	-0.2593	4.41E-01
miR-208b-3p	miR-1271-5p	-0.1350	6.92E-01
miR-219b-3p	miR-1271-5p	0.1742	6.08E-01
miR-25-5p	miR-1271-5p	0.4946	1.22E-01
miR-378g	miR-1271-5p	0.3804	2.48E-01
miR-382-3p	miR-1271-5p	-0.0811	8.13E-01
miR-432-5p	miR-1271-5p	-0.0675	8.44E-01
miR-4455	miR-1271-5p	-0.1372	6.88E-01
miR-501-3p	miR-1271-5p	-0.1727	6.12E-01
miR-506-5p	miR-1271-5p	-0.0294	9.32E-01
miR-508-3p	miR-1271-5p	0.0546	8.73E-01
miR-516b-5p	miR-1271-5p	-0.1025	7.64E-01
miR-517a-3p	miR-1271-5p	0.3486	3.23E-01
miR-519c-3p	miR-1271-5p	0.1268	7.10E-01
miR-526a+518c-5p+518d-5p	miR-1271-5p	0.1008	7.68E-01
miR-548ad-3p	miR-1271-5p	0.3650	3.00E-01
miR-548ah-5p	miR-1271-5p	-0.0117	9.73E-01
miR-548v	miR-1271-5p	0.5422	8.49E-02
miR-549a	miR-1271-5p	0.1226	7.19E-01
miR-575	miR-1271-5p	0.2322	5.18E-01
miR-593-3p	miR-1271-5p	0.1825	5.91E-01
miR-612	miR-1271-5p	0.1846	6.10E-01
miR-627-3p	miR-1271-5p	0.2270	5.02E-01
miR-744-5p	miR-1271-5p	0.0947	7.82E-01
miR-765	miR-1271-5p	0.1753	6.06E-01
miR-1245b-5p	miR-186-5p	0.1853	6.33E-01
miR-127-3p	miR-186-5p	-0.1342	7.31E-01
miR-1271-5p	miR-186-5p	0.1826	6.38E-01

miR-186-5p	miR-186-5p	-0.1709	6.60E-01
miR-196a-5p	miR-186-5p	-0.6572	5.45E-02
miR-208b-3p	miR-186-5p	-0.0958	8.06E-01
miR-219b-3p	miR-186-5p	-0.0031	9.94E-01
miR-25-5p	miR-186-5p	0.5445	1.30E-01
miR-378g	miR-186-5p	0.1788	6.45E-01
miR-382-3p	miR-186-5p	-0.1559	6.89E-01
miR-432-5p	miR-186-5p	-0.1677	6.66E-01
miR-4455	miR-186-5p	0.0424	9.14E-01
miR-501-3p	miR-186-5p	-0.6073	8.28E-02
miR-506-5p	miR-186-5p	0.0036	9.93E-01
miR-508-3p	miR-186-5p	-0.1033	7.91E-01
miR-516b-5p	miR-186-5p	-0.5644	1.13E-01
miR-517a-3p	miR-186-5p	0.4833	2.25E-01
miR-519c-3p	miR-186-5p	0.1130	7.72E-01
miR-526a+518c-5p+518d-5p	miR-186-5p	-0.1406	7.18E-01
miR-548ad-3p	miR-186-5p	0.7057	5.05E-02
miR-548ah-5p	miR-186-5p	-0.0347	9.29E-01
miR-548v	miR-186-5p	0.1250	7.49E-01
miR-549a	miR-186-5p	0.2489	5.18E-01
miR-575	miR-186-5p	0.6095	1.09E-01
miR-593-3p	miR-186-5p	0.4092	2.74E-01
miR-612	miR-186-5p	0.5073	1.99E-01
miR-627-3p	miR-186-5p	-0.1683	6.65E-01
miR-744-5p	miR-186-5p	0.2443	5.26E-01
miR-765	miR-186-5p	-0.0207	9.58E-01
miR-1245b-5p	miR-196a-5p	0.3377	3.74E-01
miR-127-3p	miR-196a-5p	0.2568	5.05E-01
miR-1271-5p	miR-196a-5p	0.0915	8.15E-01
miR-186-5p	miR-196a-5p	0.0964	8.05E-01
miR-196a-5p	miR-196a-5p	0.4090	2.74E-01
miR-208b-3p	miR-196a-5p	0.1023	7.93E-01
miR-219b-3p	miR-196a-5p	0.3267	3.91E-01
miR-25-5p	miR-196a-5p	0.1144	7.70E-01
miR-378g	miR-196a-5p	0.3293	3.87E-01
miR-382-3p	miR-196a-5p	0.2537	5.10E-01
miR-432-5p	miR-196a-5p	0.1964	6.13E-01
miR-4455	miR-196a-5p	0.0432	9.12E-01
miR-501-3p	miR-196a-5p	0.3636	3.36E-01
miR-506-5p	miR-196a-5p	0.0860	8.26E-01
miR-508-3p	miR-196a-5p	0.1307	7.37E-01
miR-516b-5p	miR-196a-5p	0.2528	5.12E-01
miR-517a-3p	miR-196a-5p	0.0233	9.56E-01
miR-519c-3p	miR-196a-5p	0.4680	2.04E-01
miR-526a+518c-5p+518d-5p	miR-196a-5p	0.3727	3.23E-01
miR-548ad-3p	miR-196a-5p	-0.1497	7.24E-01
miR-548ah-5p	miR-196a-5p	0.1707	6.61E-01

miR-548v	miR-196a-5p	0.6812	4.33E-02
miR-549a	miR-196a-5p	0.5706	1.09E-01
miR-575	miR-196a-5p	0.4567	2.55E-01
miR-593-3p	miR-196a-5p	0.0387	9.21E-01
miR-612	miR-196a-5p	0.1285	7.62E-01
miR-627-3p	miR-196a-5p	0.2997	4.33E-01
miR-744-5p	miR-196a-5p	-0.0847	8.28E-01
miR-765	miR-196a-5p	0.3604	3.41E-01
miR-1245b-5p	miR-208b-3p	0.0793	8.17E-01
miR-127-3p	miR-208b-3p	-0.2244	5.07E-01
miR-1271-5p	miR-208b-3p	0.4652	1.49E-01
miR-186-5p	miR-208b-3p	-0.0381	9.11E-01
miR-196a-5p	miR-208b-3p	-0.1651	6.28E-01
miR-208b-3p	miR-208b-3p	0.8575	7.39E-04
miR-219b-3p	miR-208b-3p	0.1212	7.23E-01
miR-25-5p	miR-208b-3p	0.1005	7.69E-01
miR-378g	miR-208b-3p	-0.0036	9.92E-01
miR-382-3p	miR-208b-3p	0.3205	3.37E-01
miR-432-5p	miR-208b-3p	0.3164	3.43E-01
miR-4455	miR-208b-3p	0.5871	5.76E-02
miR-501-3p	miR-208b-3p	0.2986	3.72E-01
miR-506-5p	miR-208b-3p	0.2462	4.65E-01
miR-508-3p	miR-208b-3p	0.3214	3.35E-01
miR-516b-5p	miR-208b-3p	-0.3381	3.09E-01
miR-517a-3p	miR-208b-3p	-0.1167	7.48E-01
miR-519c-3p	miR-208b-3p	0.5174	1.03E-01
miR-526a+518c-5p+518d-5p	miR-208b-3p	-0.2294	4.97E-01
miR-548ad-3p	miR-208b-3p	-0.0050	9.89E-01
miR-548ah-5p	miR-208b-3p	0.3368	3.11E-01
miR-548v	miR-208b-3p	-0.1224	7.20E-01
miR-549a	miR-208b-3p	0.0776	8.21E-01
miR-575	miR-208b-3p	-0.2042	5.71E-01
miR-593-3p	miR-208b-3p	0.1794	5.98E-01
miR-612	miR-208b-3p	0.6752	3.22E-02
miR-627-3p	miR-208b-3p	0.2215	5.13E-01
miR-744-5p	miR-208b-3p	0.3095	3.54E-01
miR-765	miR-208b-3p	0.1968	5.62E-01
miR-1245b-5p	miR-219b-3p	0.0464	8.92E-01
miR-127-3p	miR-219b-3p	0.1905	5.75E-01
miR-1271-5p	miR-219b-3p	-0.1144	7.38E-01
miR-186-5p	miR-219b-3p	0.4601	1.54E-01
miR-196a-5p	miR-219b-3p	-0.2837	3.98E-01
miR-208b-3p	miR-219b-3p	-0.2488	4.61E-01
miR-219b-3p	miR-219b-3p	0.0428	9.01E-01
miR-25-5p	miR-219b-3p	0.3558	2.83E-01
miR-378g	miR-219b-3p	0.1755	6.06E-01
miR-382-3p	miR-219b-3p	-0.1350	6.92E-01

miR-432-5p	miR-219b-3p	-0.2119	5.32E-01
miR-4455	miR-219b-3p	-0.2918	3.84E-01
miR-501-3p	miR-219b-3p	-0.1752	6.06E-01
miR-506-5p	miR-219b-3p	-0.1545	6.50E-01
miR-508-3p	miR-219b-3p	-0.0627	8.55E-01
miR-516b-5p	miR-219b-3p	-0.0662	8.47E-01
miR-517a-3p	miR-219b-3p	0.2692	4.52E-01
miR-519c-3p	miR-219b-3p	-0.0404	9.06E-01
miR-526a+518c-5p+518d-5p	miR-219b-3p	-0.0457	8.94E-01
miR-548ad-3p	miR-219b-3p	0.2019	5.76E-01
miR-548ah-5p	miR-219b-3p	-0.1976	5.60E-01
miR-548v	miR-219b-3p	0.4365	1.80E-01
miR-549a	miR-219b-3p	-0.0089	9.79E-01
miR-575	miR-219b-3p	0.0765	8.34E-01
miR-593-3p	miR-219b-3p	0.0314	9.27E-01
miR-612	miR-219b-3p	0.1056	7.72E-01
miR-627-3p	miR-219b-3p	0.0871	7.99E-01
miR-744-5p	miR-219b-3p	-0.0659	8.47E-01
miR-765	miR-219b-3p	0.0436	8.99E-01
miR-1245b-5p	miR-25-5p	0.1002	7.69E-01
miR-127-3p	miR-25-5p	0.0994	7.71E-01
miR-1271-5p	miR-25-5p	-0.0136	9.68E-01
miR-186-5p	miR-25-5p	0.0998	7.70E-01
miR-196a-5p	miR-25-5p	-0.2713	4.20E-01
miR-208b-3p	miR-25-5p	-0.1050	7.59E-01
miR-219b-3p	miR-25-5p	0.1096	7.48E-01
miR-25-5p	miR-25-5p	0.2303	4.96E-01
miR-378g	miR-25-5p	0.1434	6.74E-01
miR-382-3p	miR-25-5p	-0.0283	9.34E-01
miR-432-5p	miR-25-5p	-0.1606	6.37E-01
miR-4455	miR-25-5p	-0.2352	4.86E-01
miR-501-3p	miR-25-5p	0.0080	9.81E-01
miR-506-5p	miR-25-5p	-0.1730	6.11E-01
miR-508-3p	miR-25-5p	-0.0796	8.16E-01
miR-516b-5p	miR-25-5p	-0.3184	3.40E-01
miR-517a-3p	miR-25-5p	0.1787	6.21E-01
miR-519c-3p	miR-25-5p	0.0483	8.88E-01
miR-526a+518c-5p+518d-5p	miR-25-5p	0.1307	7.02E-01
miR-548ad-3p	miR-25-5p	0.0986	7.86E-01
miR-548ah-5p	miR-25-5p	-0.1361	6.90E-01
miR-548v	miR-25-5p	0.4306	1.86E-01
miR-549a	miR-25-5p	0.0149	9.65E-01
miR-575	miR-25-5p	0.1485	6.82E-01
miR-593-3p	miR-25-5p	-0.0524	8.78E-01
miR-612	miR-25-5p	0.1812	6.16E-01
miR-627-3p	miR-25-5p	0.0023	9.95E-01
miR-744-5p	miR-25-5p	-0.2130	5.29E-01

miR-765	miR-25-5p	0.0564	8.69E-01
miR-1245b-5p	miR-378g	0.2344	4.88E-01
miR-127-3p	miR-378g	0.2941	3.80E-01
miR-1271-5p	miR-378g	0.1349	6.93E-01
miR-186-5p	miR-378g	0.2205	5.15E-01
miR-196a-5p	miR-378g	-0.2411	4.75E-01
miR-208b-3p	miR-378g	-0.1484	6.63E-01
miR-219b-3p	miR-378g	0.1413	6.79E-01
miR-25-5p	miR-378g	0.5770	6.31E-02
miR-378g	miR-378g	0.3278	3.25E-01
miR-382-3p	miR-378g	0.0876	7.98E-01
miR-432-5p	miR-378g	-0.0969	7.77E-01
miR-4455	miR-378g	-0.1010	7.68E-01
miR-501-3p	miR-378g	-0.0706	8.37E-01
miR-506-5p	miR-378g	0.0613	8.58E-01
miR-508-3p	miR-378g	0.0628	8.55E-01
miR-516b-5p	miR-378g	-0.3609	2.76E-01
miR-517a-3p	miR-378g	0.5860	7.50E-02
miR-519c-3p	miR-378g	0.1376	6.87E-01
miR-526a+518c-5p+518d-5p	miR-378g	0.1408	6.80E-01
miR-548ad-3p	miR-378g	0.5442	1.04E-01
miR-548ah-5p	miR-378g	0.0080	9.81E-01
miR-548v	miR-378g	0.5162	1.04E-01
miR-549a	miR-378g	0.2368	4.83E-01
miR-575	miR-378g	0.4015	2.50E-01
miR-593-3p	miR-378g	0.3025	3.66E-01
miR-612	miR-378g	0.3743	2.87E-01
miR-627-3p	miR-378g	0.1012	7.67E-01
miR-744-5p	miR-378g	0.1159	7.34E-01
miR-765	miR-378g	0.2473	4.63E-01
miR-1245b-5p	miR-382-3p	0.0791	8.17E-01
miR-127-3p	miR-382-3p	0.1785	6.00E-01
miR-1271-5p	miR-382-3p	-0.0678	8.43E-01
miR-186-5p	miR-382-3p	0.3777	2.52E-01
miR-196a-5p	miR-382-3p	-0.3896	2.36E-01
miR-208b-3p	miR-382-3p	-0.2570	4.46E-01
miR-219b-3p	miR-382-3p	0.0421	9.02E-01
miR-25-5p	miR-382-3p	0.3677	2.66E-01
miR-378g	miR-382-3p	0.1733	6.10E-01
miR-382-3p	miR-382-3p	-0.1216	7.22E-01
miR-432-5p	miR-382-3p	-0.2121	5.31E-01
miR-4455	miR-382-3p	-0.3017	3.67E-01
miR-501-3p	miR-382-3p	-0.2988	3.72E-01
miR-506-5p	miR-382-3p	-0.1410	6.79E-01
miR-508-3p	miR-382-3p	-0.0895	7.94E-01
miR-516b-5p	miR-382-3p	-0.2066	5.42E-01
miR-517a-3p	miR-382-3p	0.2999	4.00E-01

miR-519c-3p	miR-382-3p	-0.0646	8.50E-01
miR-526a+518c-5p+518d-5p	miR-382-3p	-0.0176	9.59E-01
miR-548ad-3p	miR-382-3p	0.2251	5.32E-01
miR-548ah-5p	miR-382-3p	-0.1609	6.37E-01
miR-548v	miR-382-3p	0.3927	2.32E-01
miR-549a	miR-382-3p	-0.0206	9.52E-01
miR-575	miR-382-3p	0.1232	7.34E-01
miR-593-3p	miR-382-3p	0.0409	9.05E-01
miR-612	miR-382-3p	0.0715	8.44E-01
miR-627-3p	miR-382-3p	0.0532	8.77E-01
miR-744-5p	miR-382-3p	-0.0972	7.76E-01
miR-765	miR-382-3p	0.0419	9.03E-01
miR-1245b-5p	miR-432-5p	0.1293	7.05E-01
miR-127-3p	miR-432-5p	0.3176	3.41E-01
miR-1271-5p	miR-432-5p	-0.0439	8.98E-01
miR-186-5p	miR-432-5p	0.2795	4.05E-01
miR-196a-5p	miR-432-5p	-0.3023	3.66E-01
miR-208b-3p	miR-432-5p	-0.2155	5.25E-01
miR-219b-3p	miR-432-5p	0.0037	9.91E-01
miR-25-5p	miR-432-5p	0.4223	1.96E-01
miR-378g	miR-432-5p	0.2801	4.04E-01
miR-382-3p	miR-432-5p	-0.1346	6.93E-01
miR-432-5p	miR-432-5p	-0.2031	5.49E-01
miR-4455	miR-432-5p	-0.2791	4.06E-01
miR-501-3p	miR-432-5p	-0.3181	3.40E-01
miR-506-5p	miR-432-5p	-0.1066	7.55E-01
miR-508-3p	miR-432-5p	-0.0649	8.50E-01
miR-516b-5p	miR-432-5p	-0.2229	5.10E-01
miR-517a-3p	miR-432-5p	0.3518	3.19E-01
miR-519c-3p	miR-432-5p	-0.0193	9.55E-01
miR-526a+518c-5p+518d-5p	miR-432-5p	0.0420	9.02E-01
miR-548ad-3p	miR-432-5p	0.3467	3.26E-01
miR-548ah-5p	miR-432-5p	-0.0466	8.92E-01
miR-548v	miR-432-5p	0.5032	1.15E-01
miR-549a	miR-432-5p	0.0502	8.83E-01
miR-575	miR-432-5p	0.1699	6.39E-01
miR-593-3p	miR-432-5p	0.0907	7.91E-01
miR-612	miR-432-5p	0.1107	7.61E-01
miR-627-3p	miR-432-5p	0.1844	5.87E-01
miR-744-5p	miR-432-5p	-0.0190	9.56E-01
miR-765	miR-432-5p	0.1301	7.03E-01
miR-1245b-5p	miR-4455	0.2572	4.45E-01
miR-127-3p	miR-4455	0.0266	9.38E-01
miR-1271-5p	miR-4455	0.3085	3.56E-01
miR-186-5p	miR-4455	0.2670	4.27E-01
miR-196a-5p	miR-4455	-0.3340	3.15E-01
miR-208b-3p	miR-4455	0.2425	4.72E-01

miR-219b-3p	miR-4455	0.2639	4.33E-01
miR-25-5p	miR-4455	0.4884	1.27E-01
miR-378g	miR-4455	0.2350	4.87E-01
miR-382-3p	miR-4455	0.2248	5.06E-01
miR-432-5p	miR-4455	0.1077	7.53E-01
miR-4455	miR-4455	0.1670	6.24E-01
miR-501-3p	miR-4455	0.0442	8.97E-01
miR-506-5p	miR-4455	0.1307	7.02E-01
miR-508-3p	miR-4455	0.1495	6.61E-01
miR-516b-5p	miR-4455	-0.4053	2.16E-01
miR-517a-3p	miR-4455	0.3641	3.01E-01
miR-519c-3p	miR-4455	0.4100	2.10E-01
miR-526a+518c-5p+518d-5p	miR-4455	-0.0316	9.27E-01
miR-548ad-3p	miR-4455	0.2776	4.37E-01
miR-548ah-5p	miR-4455	0.0963	7.78E-01
miR-548v	miR-4455	0.3405	3.06E-01
miR-549a	miR-4455	0.2590	4.42E-01
miR-575	miR-4455	0.2631	4.63E-01
miR-593-3p	miR-4455	0.2825	4.00E-01
miR-612	miR-4455	0.5324	1.13E-01
miR-627-3p	miR-4455	0.1399	6.82E-01
miR-744-5p	miR-4455	0.1325	6.98E-01
miR-765	miR-4455	0.2887	3.89E-01
miR-1245b-5p	miR-501-3p	-0.1666	6.24E-01
miR-127-3p	miR-501-3p	-0.0751	8.26E-01
miR-1271-5p	miR-501-3p	-0.1782	6.00E-01
miR-186-5p	miR-501-3p	0.2923	3.83E-01
miR-196a-5p	miR-501-3p	-0.5103	1.09E-01
miR-208b-3p	miR-501-3p	-0.0446	8.96E-01
miR-219b-3p	miR-501-3p	-0.1249	7.15E-01
miR-25-5p	miR-501-3p	0.0490	8.86E-01
miR-378g	miR-501-3p	-0.0865	8.00E-01
miR-382-3p	miR-501-3p	-0.2680	4.26E-01
miR-432-5p	miR-501-3p	-0.2942	3.80E-01
miR-4455	miR-501-3p	-0.3189	3.39E-01
miR-501-3p	miR-501-3p	-0.2691	4.24E-01
miR-506-5p	miR-501-3p	-0.3397	3.07E-01
miR-508-3p	miR-501-3p	-0.1842	5.88E-01
miR-516b-5p	miR-501-3p	-0.2129	5.30E-01
miR-517a-3p	miR-501-3p	-0.1190	7.43E-01
miR-519c-3p	miR-501-3p	-0.1634	6.31E-01
miR-526a+518c-5p+518d-5p	miR-501-3p	-0.2560	4.47E-01
miR-548ad-3p	miR-501-3p	-0.1392	7.01E-01
miR-548ah-5p	miR-501-3p	-0.2703	4.21E-01
miR-548v	miR-501-3p	0.1455	6.70E-01
miR-549a	miR-501-3p	-0.3286	3.24E-01
miR-575	miR-501-3p	-0.2828	4.29E-01

miR-593-3p	miR-501-3p	-0.2576	4.44E-01
miR-612	miR-501-3p	0.0240	9.48E-01
miR-627-3p	miR-501-3p	-0.0147	9.66E-01
miR-744-5p	miR-501-3p	-0.2616	4.37E-01
miR-765	miR-501-3p	-0.1880	5.80E-01
miR-1245b-5p	miR-506-5p	0.2151	5.25E-01
miR-127-3p	miR-506-5p	0.1406	6.80E-01
miR-1271-5p	miR-506-5p	0.0784	8.19E-01
miR-186-5p	miR-506-5p	0.3479	2.94E-01
miR-196a-5p	miR-506-5p	-0.2959	3.77E-01
miR-208b-3p	miR-506-5p	-0.1361	6.90E-01
miR-219b-3p	miR-506-5p	0.1973	5.61E-01
miR-25-5p	miR-506-5p	0.5054	1.13E-01
miR-378g	miR-506-5p	0.2787	4.07E-01
miR-382-3p	miR-506-5p	0.0178	9.58E-01
miR-432-5p	miR-506-5p	-0.0563	8.69E-01
miR-4455	miR-506-5p	-0.1087	7.50E-01
miR-501-3p	miR-506-5p	-0.0796	8.16E-01
miR-506-5p	miR-506-5p	-0.0141	9.67E-01
miR-508-3p	miR-506-5p	-0.0015	9.96E-01
miR-516b-5p	miR-506-5p	-0.2654	4.30E-01
miR-517a-3p	miR-506-5p	0.4273	2.18E-01
miR-519c-3p	miR-506-5p	0.1924	5.71E-01
miR-526a+518c-5p+518d-5p	miR-506-5p	0.0146	9.66E-01
miR-548ad-3p	miR-506-5p	0.3030	3.95E-01
miR-548ah-5p	miR-506-5p	-0.0800	8.15E-01
miR-548v	miR-506-5p	0.4190	2.00E-01
miR-549a	miR-506-5p	0.1973	5.61E-01
miR-575	miR-506-5p	0.3275	3.56E-01
miR-593-3p	miR-506-5p	0.2073	5.41E-01
miR-612	miR-506-5p	0.2979	4.03E-01
miR-627-3p	miR-506-5p	0.0820	8.11E-01
miR-744-5p	miR-506-5p	0.0201	9.53E-01
miR-765	miR-506-5p	0.2155	5.24E-01
miR-1245b-5p	miR-508-3p	0.1257	7.13E-01
miR-127-3p	miR-508-3p	0.1171	7.32E-01
miR-1271-5p	miR-508-3p	-0.0133	9.69E-01
miR-186-5p	miR-508-3p	0.4139	2.06E-01
miR-196a-5p	miR-508-3p	-0.3214	3.35E-01
miR-208b-3p	miR-508-3p	-0.0751	8.26E-01
miR-219b-3p	miR-508-3p	0.1652	6.27E-01
miR-25-5p	miR-508-3p	0.2760	4.11E-01
miR-378g	miR-508-3p	0.2193	5.17E-01
miR-382-3p	miR-508-3p	-0.1061	7.56E-01
miR-432-5p	miR-508-3p	-0.0885	7.96E-01
miR-4455	miR-508-3p	-0.1910	5.74E-01
miR-501-3p	miR-508-3p	-0.0717	8.34E-01

miR-506-5p	miR-508-3p	-0.1432	6.74E-01
miR-508-3p	miR-508-3p	0.0220	9.49E-01
miR-516b-5p	miR-508-3p	-0.0916	7.89E-01
miR-517a-3p	miR-508-3p	0.0990	7.85E-01
miR-519c-3p	miR-508-3p	0.0276	9.36E-01
miR-526a+518c-5p+518d-5p	miR-508-3p	0.0620	8.56E-01
miR-548ad-3p	miR-508-3p	0.0618	8.65E-01
miR-548ah-5p	miR-508-3p	-0.1170	7.32E-01
miR-548v	miR-508-3p	0.3949	2.29E-01
miR-549a	miR-508-3p	-0.1090	7.50E-01
miR-575	miR-508-3p	-0.0154	9.66E-01
miR-593-3p	miR-508-3p	-0.0345	9.20E-01
miR-612	miR-508-3p	0.0668	8.54E-01
miR-627-3p	miR-508-3p	0.1364	6.89E-01
miR-744-5p	miR-508-3p	-0.0873	7.98E-01
miR-765	miR-508-3p	0.0302	9.30E-01
miR-1245b-5p	miR-516b-5p	0.3223	3.64E-01
miR-127-3p	miR-516b-5p	0.2928	4.12E-01
miR-1271-5p	miR-516b-5p	0.1054	7.72E-01
miR-186-5p	miR-516b-5p	-0.0567	8.76E-01
miR-196a-5p	miR-516b-5p	0.0916	8.01E-01
miR-208b-3p	miR-516b-5p	-0.1161	7.49E-01
miR-219b-3p	miR-516b-5p	0.2141	5.53E-01
miR-25-5p	miR-516b-5p	0.3597	3.07E-01
miR-378g	miR-516b-5p	0.3232	3.62E-01
miR-382-3p	miR-516b-5p	0.1899	5.99E-01
miR-432-5p	miR-516b-5p	-0.0229	9.50E-01
miR-4455	miR-516b-5p	-0.0994	7.85E-01
miR-501-3p	miR-516b-5p	0.0544	8.81E-01
miR-506-5p	miR-516b-5p	0.0558	8.78E-01
miR-508-3p	miR-516b-5p	0.0129	9.72E-01
miR-516b-5p	miR-516b-5p	-0.1471	6.85E-01
miR-517a-3p	miR-516b-5p	0.4191	2.62E-01
miR-519c-3p	miR-516b-5p	0.3462	3.27E-01
miR-526a+518c-5p+518d-5p	miR-516b-5p	0.3415	3.34E-01
miR-548ad-3p	miR-516b-5p	0.3855	3.06E-01
miR-548ah-5p	miR-516b-5p	0.0886	8.08E-01
miR-548v	miR-516b-5p	0.7144	2.03E-02
miR-549a	miR-516b-5p	0.6033	6.48E-02
miR-575	miR-516b-5p	0.6231	7.30E-02
miR-593-3p	miR-516b-5p	0.2254	5.31E-01
miR-612	miR-516b-5p	0.3220	3.98E-01
miR-627-3p	miR-516b-5p	0.1391	7.02E-01
miR-744-5p	miR-516b-5p	-0.0309	9.33E-01
miR-765	miR-516b-5p	0.3233	3.62E-01
miR-1245b-5p	miR-517a-3p	0.4287	2.16E-01
miR-127-3p	miR-517a-3p	0.1114	7.59E-01

miR-1271-5p	miR-517a-3p	0.3967	2.56E-01
miR-186-5p	miR-517a-3p	0.2019	5.76E-01
miR-196a-5p	miR-517a-3p	-0.2782	4.36E-01
miR-208b-3p	miR-517a-3p	0.0793	8.28E-01
miR-219b-3p	miR-517a-3p	0.3262	3.58E-01
miR-25-5p	miR-517a-3p	0.7748	8.49E-03
miR-378g	miR-517a-3p	0.4385	2.05E-01
miR-382-3p	miR-517a-3p	0.2208	5.40E-01
miR-432-5p	miR-517a-3p	0.1235	7.34E-01
miR-4455	miR-517a-3p	0.2729	4.46E-01
miR-501-3p	miR-517a-3p	0.0349	9.24E-01
miR-506-5p	miR-517a-3p	0.2658	4.58E-01
miR-508-3p	miR-517a-3p	0.1916	5.96E-01
miR-516b-5p	miR-517a-3p	-0.4119	2.37E-01
miR-517a-3p	miR-517a-3p	0.7189	2.91E-02
miR-519c-3p	miR-517a-3p	0.5352	1.11E-01
miR-526a+518c-5p+518d-5p	miR-517a-3p	0.0717	8.44E-01
miR-548ad-3p	miR-517a-3p	0.7449	2.13E-02
miR-548ah-5p	miR-517a-3p	0.1376	7.05E-01
miR-548v	miR-517a-3p	0.4457	1.97E-01
miR-549a	miR-517a-3p	0.5970	6.84E-02
miR-575	miR-517a-3p	0.7675	1.58E-02
miR-593-3p	miR-517a-3p	0.6340	4.90E-02
miR-612	miR-517a-3p	0.7317	2.50E-02
miR-627-3p	miR-517a-3p	0.1055	7.72E-01
miR-744-5p	miR-517a-3p	0.4003	2.52E-01
miR-765	miR-517a-3p	0.4014	2.50E-01
miR-1245b-5p	miR-519c-3p	-0.0165	9.62E-01
miR-127-3p	miR-519c-3p	0.0779	8.20E-01
miR-1271-5p	miR-519c-3p	-0.0889	7.95E-01
miR-186-5p	miR-519c-3p	0.2214	5.13E-01
miR-196a-5p	miR-519c-3p	-0.4765	1.38E-01
miR-208b-3p	miR-519c-3p	-0.0815	8.12E-01
miR-219b-3p	miR-519c-3p	-0.0405	9.06E-01
miR-25-5p	miR-519c-3p	0.1571	6.45E-01
miR-378g	miR-519c-3p	0.0742	8.28E-01
miR-382-3p	miR-519c-3p	-0.2083	5.39E-01
miR-432-5p	miR-519c-3p	-0.2418	4.74E-01
miR-4455	miR-519c-3p	-0.3095	3.54E-01
miR-501-3p	miR-519c-3p	-0.2912	3.85E-01
miR-506-5p	miR-519c-3p	-0.2585	4.43E-01
miR-508-3p	miR-519c-3p	-0.1208	7.23E-01
miR-516b-5p	miR-519c-3p	-0.2839	3.98E-01
miR-517a-3p	miR-519c-3p	0.0153	9.66E-01
miR-519c-3p	miR-519c-3p	-0.1330	6.97E-01
miR-526a+518c-5p+518d-5p	miR-519c-3p	-0.0452	8.95E-01
miR-548ad-3p	miR-519c-3p	0.0190	9.58E-01

miR-548ah-5p	miR-519c-3p	-0.1503	6.59E-01
miR-548v	miR-519c-3p	0.2877	3.91E-01
miR-549a	miR-519c-3p	-0.2481	4.62E-01
miR-575	miR-519c-3p	-0.1311	7.18E-01
miR-593-3p	miR-519c-3p	-0.1652	6.27E-01
miR-612	miR-519c-3p	0.0066	9.86E-01
miR-627-3p	miR-519c-3p	0.0615	8.57E-01
miR-744-5p	miR-519c-3p	-0.2112	5.33E-01
miR-765	miR-519c-3p	-0.0882	7.97E-01
miR-1245b-5p	miR-526a+518c-5p+518d-5p	0.4244	2.22E-01
miR-127-3p	miR-526a+518c-5p+518d-5p	0.3804	2.78E-01
miR-1271-5p	miR-526a+518c-5p+518d-5p	0.0932	7.98E-01
miR-186-5p	miR-526a+518c-5p+518d-5p	0.0573	8.75E-01
miR-196a-5p	miR-526a+518c-5p+518d-5p	0.0512	8.88E-01
miR-208b-3p	miR-526a+518c-5p+518d-5p	-0.3686	2.95E-01
miR-219b-3p	miR-526a+518c-5p+518d-5p	0.2667	4.56E-01
miR-25-5p	miR-526a+518c-5p+518d-5p	0.5245	1.20E-01
miR-378g	miR-526a+518c-5p+518d-5p	0.4511	1.91E-01
miR-382-3p	miR-526a+518c-5p+518d-5p	0.1264	7.28E-01
miR-432-5p	miR-526a+518c-5p+518d-5p	-0.0114	9.75E-01
miR-4455	miR-526a+518c-5p+518d-5p	-0.1707	6.37E-01
miR-501-3p	miR-526a+518c-5p+518d-5p	-0.1014	7.81E-01
miR-506-5p	miR-526a+518c-5p+518d-5p	0.1149	7.52E-01
miR-508-3p	miR-526a+518c-5p+518d-5p	-0.0055	9.88E-01
miR-516b-5p	miR-526a+518c-5p+518d-5p	-0.1403	6.99E-01
miR-517a-3p	miR-526a+518c-5p+518d-5p	0.6314	6.82E-02
miR-519c-3p	miR-526a+518c-5p+518d-5p	0.2494	4.87E-01
miR-526a+518c-5p+518d-5p	miR-526a+518c-5p+518d-5p	0.4016	2.50E-01
miR-548ad-3p	miR-526a+518c-5p+518d-5p	0.5461	1.28E-01
miR-548ah-5p	miR-526a+518c-5p+518d-5p	0.0732	8.41E-01
miR-548v	miR-526a+518c-5p+518d-5p	0.6659	3.56E-02
miR-549a	miR-526a+518c-5p+518d-5p	0.6267	5.25E-02
miR-575	miR-526a+518c-5p+518d-5p	0.8355	5.05E-03
miR-593-3p	miR-526a+518c-5p+518d-5p	0.3637	3.01E-01
miR-612	miR-526a+518c-5p+518d-5p	0.1739	6.54E-01
miR-627-3p	miR-526a+518c-5p+518d-5p	0.1140	7.54E-01
miR-744-5p	miR-526a+518c-5p+518d-5p	0.0411	9.10E-01
miR-765	miR-526a+518c-5p+518d-5p	0.3727	2.89E-01
miR-1245b-5p	miR-548ad-3p	0.1116	7.44E-01
miR-127-3p	miR-548ad-3p	0.0244	9.43E-01
miR-1271-5p	miR-548ad-3p	-0.0368	9.15E-01
miR-186-5p	miR-548ad-3p	0.3411	3.05E-01
miR-196a-5p	miR-548ad-3p	-0.4629	1.52E-01
miR-208b-3p	miR-548ad-3p	-0.1525	6.54E-01
miR-219b-3p	miR-548ad-3p	0.1081	7.52E-01
miR-25-5p	miR-548ad-3p	0.3348	3.14E-01
miR-378g	miR-548ad-3p	0.1938	5.68E-01

miR-382-3p	miR-548ad-3p	-0.1992	5.57E-01
miR-432-5p	miR-548ad-3p	-0.1444	6.72E-01
miR-4455	miR-548ad-3p	-0.2115	5.32E-01
miR-501-3p	miR-548ad-3p	-0.2570	4.46E-01
miR-506-5p	miR-548ad-3p	-0.1766	6.03E-01
miR-508-3p	miR-548ad-3p	-0.0812	8.12E-01
miR-516b-5p	miR-548ad-3p	-0.1971	5.61E-01
miR-517a-3p	miR-548ad-3p	0.1616	6.56E-01
miR-519c-3p	miR-548ad-3p	0.0054	9.88E-01
miR-526a+518c-5p+518d-5p	miR-548ad-3p	-0.0539	8.75E-01
miR-548ad-3p	miR-548ad-3p	0.1373	7.05E-01
miR-548ah-5p	miR-548ad-3p	-0.1682	6.21E-01
miR-548v	miR-548ad-3p	0.2939	3.80E-01
miR-549a	miR-548ad-3p	-0.0800	8.15E-01
miR-575	miR-548ad-3p	0.0928	7.99E-01
miR-593-3p	miR-548ad-3p	0.0117	9.73E-01
miR-612	miR-548ad-3p	0.0894	8.06E-01
miR-627-3p	miR-548ad-3p	0.0428	9.01E-01
miR-744-5p	miR-548ad-3p	-0.0767	8.23E-01
miR-765	miR-548ad-3p	-0.0078	9.82E-01
miR-1245b-5p	miR-548ah-5p	0.0540	8.75E-01
miR-127-3p	miR-548ah-5p	0.1186	7.28E-01
miR-1271-5p	miR-548ah-5p	-0.0487	8.87E-01
miR-186-5p	miR-548ah-5p	0.3632	2.72E-01
miR-196a-5p	miR-548ah-5p	-0.3904	2.35E-01
miR-208b-3p	miR-548ah-5p	-0.0673	8.44E-01
miR-219b-3p	miR-548ah-5p	0.0456	8.94E-01
miR-25-5p	miR-548ah-5p	0.2607	4.39E-01
miR-378g	miR-548ah-5p	0.1604	6.38E-01
miR-382-3p	miR-548ah-5p	-0.1572	6.44E-01
miR-432-5p	miR-548ah-5p	-0.1738	6.09E-01
miR-4455	miR-548ah-5p	-0.2278	5.00E-01
miR-501-3p	miR-548ah-5p	-0.2196	5.17E-01
miR-506-5p	miR-548ah-5p	-0.1779	6.01E-01
miR-508-3p	miR-548ah-5p	-0.0275	9.36E-01
miR-516b-5p	miR-548ah-5p	-0.1313	7.00E-01
miR-517a-3p	miR-548ah-5p	0.0897	8.05E-01
miR-519c-3p	miR-548ah-5p	-0.0290	9.33E-01
miR-526a+518c-5p+518d-5p	miR-548ah-5p	-0.0278	9.35E-01
miR-548ad-3p	miR-548ah-5p	0.1149	7.52E-01
miR-548ah-5p	miR-548ah-5p	-0.1209	7.23E-01
miR-548v	miR-548ah-5p	0.3933	2.31E-01
miR-549a	miR-548ah-5p	-0.1280	7.08E-01
miR-575	miR-548ah-5p	-0.0618	8.65E-01
miR-593-3p	miR-548ah-5p	-0.0496	8.85E-01
miR-612	miR-548ah-5p	0.0860	8.13E-01
miR-627-3p	miR-548ah-5p	0.1320	6.99E-01

miR-744-5p	miR-548ah-5p	-0.0804	8.14E-01
miR-765	miR-548ah-5p	-0.0220	9.49E-01
miR-1245b-5p	miR-548v	0.0733	8.30E-01
miR-127-3p	miR-548v	-0.0013	9.97E-01
miR-1271-5p	miR-548v	0.0805	8.14E-01
miR-186-5p	miR-548v	0.1358	6.91E-01
miR-196a-5p	miR-548v	-0.4701	1.45E-01
miR-208b-3p	miR-548v	0.1183	7.29E-01
miR-219b-3p	miR-548v	0.0007	9.98E-01
miR-25-5p	miR-548v	0.3809	2.48E-01
miR-378g	miR-548v	0.1397	6.82E-01
miR-382-3p	miR-548v	-0.0879	7.97E-01
miR-432-5p	miR-548v	-0.1458	6.69E-01
miR-4455	miR-548v	-0.0508	8.82E-01
miR-501-3p	miR-548v	-0.2278	5.01E-01
miR-506-5p	miR-548v	-0.1095	7.49E-01
miR-508-3p	miR-548v	-0.0582	8.65E-01
miR-516b-5p	miR-548v	-0.4072	2.14E-01
miR-517a-3p	miR-548v	0.2192	5.43E-01
miR-519c-3p	miR-548v	0.1864	5.83E-01
miR-526a+518c-5p+518d-5p	miR-548v	-0.1892	5.77E-01
miR-548ad-3p	miR-548v	0.2716	4.48E-01
miR-548ah-5p	miR-548v	-0.0446	8.96E-01
miR-548v	miR-548v	0.3296	3.22E-01
miR-549a	miR-548v	0.0827	8.09E-01
miR-575	miR-548v	0.1237	7.34E-01
miR-593-3p	miR-548v	0.1057	7.57E-01
miR-612	miR-548v	0.4261	2.19E-01
miR-627-3p	miR-548v	0.0939	7.84E-01
miR-744-5p	miR-548v	0.0293	9.32E-01
miR-765	miR-548v	0.0828	8.09E-01
miR-1245b-5p	miR-549a	0.3138	3.47E-01
miR-127-3p	miR-549a	0.2473	4.64E-01
miR-1271-5p	miR-549a	0.1085	7.51E-01
miR-186-5p	miR-549a	0.3026	3.66E-01
miR-196a-5p	miR-549a	-0.1802	5.96E-01
miR-208b-3p	miR-549a	-0.1715	6.14E-01
miR-219b-3p	miR-549a	0.2551	4.49E-01
miR-25-5p	miR-549a	0.5903	5.59E-02
miR-378g	miR-549a	0.4196	1.99E-01
miR-382-3p	miR-549a	0.0182	9.58E-01
miR-432-5p	miR-549a	-0.0287	9.33E-01
miR-4455	miR-549a	-0.0694	8.39E-01
miR-501-3p	miR-549a	-0.0175	9.59E-01
miR-506-5p	miR-549a	0.0406	9.06E-01
miR-508-3p	miR-549a	0.0782	8.19E-01
miR-516b-5p	miR-549a	-0.1649	6.28E-01

miR-517a-3p	miR-549a	0.5098	1.32E-01
miR-519c-3p	miR-549a	0.2264	5.03E-01
miR-526a+518c-5p+518d-5p	miR-549a	0.1735	6.10E-01
miR-548ad-3p	miR-549a	0.4908	1.50E-01
miR-548ah-5p	miR-549a	-0.0183	9.57E-01
miR-548v	miR-549a	0.5791	6.19E-02
miR-549a	miR-549a	0.2916	3.84E-01
miR-575	miR-549a	0.4510	1.91E-01
miR-593-3p	miR-549a	0.3077	3.57E-01
miR-612	miR-549a	0.3351	3.44E-01
miR-627-3p	miR-549a	0.1446	6.71E-01
miR-744-5p	miR-549a	0.1448	6.71E-01
miR-765	miR-549a	0.2505	4.58E-01
miR-1245b-5p	miR-575	-0.0073	9.84E-01
miR-127-3p	miR-575	0.0310	9.32E-01
miR-1271-5p	miR-575	-0.1860	6.07E-01
miR-186-5p	miR-575	-0.1314	7.18E-01
miR-196a-5p	miR-575	0.0671	8.54E-01
miR-208b-3p	miR-575	-0.1794	6.20E-01
miR-219b-3p	miR-575	-0.0363	9.21E-01
miR-25-5p	miR-575	0.2141	5.53E-01
miR-378g	miR-575	0.0725	8.42E-01
miR-382-3p	miR-575	-0.0968	7.90E-01
miR-432-5p	miR-575	-0.2554	4.76E-01
miR-4455	miR-575	-0.2133	5.54E-01
miR-501-3p	miR-575	0.1640	6.51E-01
miR-506-5p	miR-575	-0.2235	5.35E-01
miR-508-3p	miR-575	-0.2149	5.51E-01
miR-516b-5p	miR-575	-0.1031	7.77E-01
miR-517a-3p	miR-575	0.2521	5.13E-01
miR-519c-3p	miR-575	0.2248	5.32E-01
miR-526a+518c-5p+518d-5p	miR-575	-0.0512	8.88E-01
miR-548ad-3p	miR-575	0.2253	5.60E-01
miR-548ah-5p	miR-575	-0.2512	4.84E-01
miR-548v	miR-575	0.4805	1.60E-01
miR-549a	miR-575	0.4409	2.02E-01
miR-575	miR-575	0.4491	2.25E-01
miR-593-3p	miR-575	0.0420	9.08E-01
miR-612	miR-575	0.4564	2.17E-01
miR-627-3p	miR-575	-0.0688	8.50E-01
miR-744-5p	miR-575	-0.1291	7.22E-01
miR-765	miR-575	0.1034	7.76E-01
miR-1245b-5p	miR-593-3p	0.0995	7.71E-01
miR-127-3p	miR-593-3p	0.1789	5.99E-01
miR-1271-5p	miR-593-3p	-0.0137	9.68E-01
miR-186-5p	miR-593-3p	0.3063	3.60E-01
miR-196a-5p	miR-593-3p	-0.4583	1.56E-01

miR-208b-3p	miR-593-3p	-0.1789	5.99E-01
miR-219b-3p	miR-593-3p	-0.0093	9.78E-01
miR-25-5p	miR-593-3p	0.4465	1.69E-01
miR-378g	miR-593-3p	0.2142	5.27E-01
miR-382-3p	miR-593-3p	-0.1560	6.47E-01
miR-432-5p	miR-593-3p	-0.2100	5.35E-01
miR-4455	miR-593-3p	-0.2265	5.03E-01
miR-501-3p	miR-593-3p	-0.4166	2.03E-01
miR-506-5p	miR-593-3p	-0.1040	7.61E-01
miR-508-3p	miR-593-3p	-0.0773	8.21E-01
miR-516b-5p	miR-593-3p	-0.2779	4.08E-01
miR-517a-3p	miR-593-3p	0.3469	3.26E-01
miR-519c-3p	miR-593-3p	-0.0192	9.55E-01
miR-526a+518c-5p+518d-5p	miR-593-3p	-0.0847	8.04E-01
miR-548ad-3p	miR-593-3p	0.3665	2.98E-01
miR-548ah-5p	miR-593-3p	-0.0851	8.03E-01
miR-548v	miR-593-3p	0.3808	2.48E-01
miR-549a	miR-593-3p	0.0224	9.48E-01
miR-575	miR-593-3p	0.1626	6.54E-01
miR-593-3p	miR-593-3p	0.1277	7.08E-01
miR-612	miR-593-3p	0.1681	6.42E-01
miR-627-3p	miR-593-3p	0.1023	7.65E-01
miR-744-5p	miR-593-3p	0.0239	9.44E-01
miR-765	miR-593-3p	0.0619	8.56E-01
miR-1245b-5p	miR-612	-0.1102	7.47E-01
miR-127-3p	miR-612	-0.1346	6.93E-01
miR-1271-5p	miR-612	-0.0554	8.71E-01
miR-186-5p	miR-612	0.1934	5.69E-01
miR-196a-5p	miR-612	-0.4461	1.69E-01
miR-208b-3p	miR-612	0.1719	6.13E-01
miR-219b-3p	miR-612	-0.0885	7.96E-01
miR-25-5p	miR-612	0.1454	6.70E-01
miR-378g	miR-612	-0.0445	8.97E-01
miR-382-3p	miR-612	-0.1721	6.13E-01
miR-432-5p	miR-612	-0.2088	5.38E-01
miR-4455	miR-612	-0.1089	7.50E-01
miR-501-3p	miR-612	-0.1381	6.85E-01
miR-506-5p	miR-612	-0.2463	4.65E-01
miR-508-3p	miR-612	-0.1110	7.45E-01
miR-516b-5p	miR-612	-0.2526	4.54E-01
miR-517a-3p	miR-612	-0.0592	8.71E-01
miR-519c-3p	miR-612	0.0906	7.91E-01
miR-526a+518c-5p+518d-5p	miR-612	-0.3236	3.32E-01
miR-548ad-3p	miR-612	-0.0095	9.79E-01
miR-548ah-5p	miR-612	-0.1742	6.08E-01
miR-548v	miR-612	0.2138	5.28E-01
miR-549a	miR-612	-0.1066	7.55E-01

miR-575	miR-612	-0.1608	6.57E-01
miR-593-3p	miR-612	-0.1095	7.49E-01
miR-612	miR-612	0.3356	3.43E-01
miR-627-3p	miR-612	0.0424	9.02E-01
miR-744-5p	miR-612	-0.0994	7.71E-01
miR-765	miR-612	-0.0784	8.19E-01
miR-1245b-5p	miR-627-3p	-0.0602	8.60E-01
miR-127-3p	miR-627-3p	-0.0771	8.22E-01
miR-1271-5p	miR-627-3p	-0.1412	6.79E-01
miR-186-5p	miR-627-3p	0.3053	3.61E-01
miR-196a-5p	miR-627-3p	-0.3870	2.40E-01
miR-208b-3p	miR-627-3p	-0.0589	8.63E-01
miR-219b-3p	miR-627-3p	0.0218	9.49E-01
miR-25-5p	miR-627-3p	0.0752	8.26E-01
miR-378g	miR-627-3p	-0.0081	9.81E-01
miR-382-3p	miR-627-3p	-0.1951	5.65E-01
miR-432-5p	miR-627-3p	-0.2065	5.42E-01
miR-4455	miR-627-3p	-0.2652	4.31E-01
miR-501-3p	miR-627-3p	-0.1186	7.28E-01
miR-506-5p	miR-627-3p	-0.2937	3.81E-01
miR-508-3p	miR-627-3p	-0.1313	7.00E-01
miR-516b-5p	miR-627-3p	-0.1029	7.63E-01
miR-517a-3p	miR-627-3p	-0.0969	7.90E-01
miR-519c-3p	miR-627-3p	-0.0447	8.96E-01
miR-526a+518c-5p+518d-5p	miR-627-3p	-0.1267	7.10E-01
miR-548ad-3p	miR-627-3p	-0.1399	7.00E-01
miR-548ah-5p	miR-627-3p	-0.2593	4.41E-01
miR-548v	miR-627-3p	0.2577	4.44E-01
miR-549a	miR-627-3p	-0.1918	5.72E-01
miR-575	miR-627-3p	-0.1511	6.77E-01
miR-593-3p	miR-627-3p	-0.2077	5.40E-01
miR-612	miR-627-3p	0.0527	8.85E-01
miR-627-3p	miR-627-3p	-0.0084	9.80E-01
miR-744-5p	miR-627-3p	-0.2487	4.61E-01
miR-765	miR-627-3p	-0.1280	7.08E-01
miR-1245b-5p	miR-744-5p	0.1015	7.80E-01
miR-127-3p	miR-744-5p	-0.1219	7.37E-01
miR-1271-5p	miR-744-5p	0.1258	7.29E-01
miR-186-5p	miR-744-5p	-0.0982	7.87E-01
miR-196a-5p	miR-744-5p	-0.4571	1.84E-01
miR-208b-3p	miR-744-5p	0.1909	5.97E-01
miR-219b-3p	miR-744-5p	-0.0378	9.18E-01
miR-25-5p	miR-744-5p	0.4883	1.52E-01
miR-378g	miR-744-5p	0.2081	5.64E-01
miR-382-3p	miR-744-5p	-0.2105	5.59E-01
miR-432-5p	miR-744-5p	-0.1263	7.28E-01
miR-4455	miR-744-5p	0.1371	7.06E-01

miR-501-3p	miR-744-5p	-0.1621	6.55E-01
miR-506-5p	miR-744-5p	-0.0929	7.98E-01
miR-508-3p	miR-744-5p	-0.0731	8.41E-01
miR-516b-5p	miR-744-5p	-0.4592	1.82E-01
miR-517a-3p	miR-744-5p	0.2945	4.42E-01
miR-519c-3p	miR-744-5p	0.3441	3.30E-01
miR-526a+518c-5p+518d-5p	miR-744-5p	-0.3024	3.96E-01
miR-548ad-3p	miR-744-5p	0.4994	1.71E-01
miR-548ah-5p	miR-744-5p	-0.0287	9.37E-01
miR-548v	miR-744-5p	0.2053	5.69E-01
miR-549a	miR-744-5p	0.2358	5.12E-01
miR-575	miR-744-5p	0.3563	3.47E-01
miR-593-3p	miR-744-5p	0.2866	4.22E-01
miR-612	miR-744-5p	0.6874	4.07E-02
miR-627-3p	miR-744-5p	0.0512	8.88E-01
miR-744-5p	miR-744-5p	0.2522	4.82E-01
miR-765	miR-744-5p	0.1030	7.77E-01
miR-1245b-5p	miR-765	0.3579	3.10E-01
miR-127-3p	miR-765	0.0694	8.49E-01
miR-1271-5p	miR-765	0.2563	4.75E-01
miR-186-5p	miR-765	-0.0920	8.00E-01
miR-196a-5p	miR-765	-0.4372	2.06E-01
miR-208b-3p	miR-765	-0.1002	7.83E-01
miR-219b-3p	miR-765	0.2010	5.78E-01
miR-25-5p	miR-765	0.6146	5.86E-02
miR-378g	miR-765	0.3563	3.12E-01
miR-382-3p	miR-765	0.0283	9.38E-01
miR-432-5p	miR-765	-0.0469	8.98E-01
miR-4455	miR-765	0.0208	9.55E-01
miR-501-3p	miR-765	-0.2155	5.50E-01
miR-506-5p	miR-765	0.0697	8.48E-01
miR-508-3p	miR-765	-0.0101	9.78E-01
miR-516b-5p	miR-765	-0.5662	8.80E-02
miR-517a-3p	miR-765	0.5970	8.97E-02
miR-519c-3p	miR-765	0.2715	4.48E-01
miR-526a+518c-5p+518d-5p	miR-765	0.1384	7.03E-01
miR-548ad-3p	miR-765	0.6517	5.72E-02
miR-548ah-5p	miR-765	0.0440	9.04E-01
miR-548v	miR-765	0.3720	2.90E-01
miR-549a	miR-765	0.3906	2.64E-01
miR-575	miR-765	0.7452	2.12E-02
miR-593-3p	miR-765	0.4203	2.27E-01
miR-612	miR-765	0.4789	1.92E-01
miR-627-3p	miR-765	-0.0322	9.30E-01
miR-744-5p	miR-765	0.1562	6.67E-01
miR-765	miR-765	0.2195	5.42E-01

Supplementary Table 8a: VENN diagram from Figure 7A in alphabetic order

Plasma HPV negative	Overlap plasma HPV negative and positive		Plasma HPV positive		
miR-125b-5p	let-7a-5p	miR-3614-5p	let-7c-5p	miR-340-5p	miR-577
miR-1272	let-7b-5p	miR-365a-3p+miR-365b-3p	miR-1-5p	miR-342-5p	miR-579-5p
miR-1972	let-7d-5p	miR-370-3p	miR-105-5p	miR-3605-3p	miR-582-3p
miR-2110	let-7f-5p	miR-371a-5p	miR-106b-5p	miR-3613-3p	miR-582-5p
miR-328-5p	let-7g-5p	miR-374a-5p	miR-1180-3p	miR-3614-3p	miR-584-5p
miR-4516	miR-100-5p	miR-376a-3p	miR-1183	miR-363-3p	miR-590-5p
miR-496	miR-103a-3p	miR-377-3p	miR-1185-2-3p	miR-365b-5p	miR-595
miR-502-5p	miR-106a-5p+miR-17-5p	miR-378e	miR-1193	miR-367-3p	miR-601
miR-517c-3p+miR-519a-3p	miR-107	miR-378f	miR-1200	miR-369-3p	miR-605-5p
miR-598-3p	miR-10b-5p	miR-378h	miR-1202	miR-371b-5p	miR-610
	miR-1178-3p	miR-378i	miR-1206	miR-374a-3p	miR-616-3p
	miR-1185-1-3p	miR-379-5p	miR-1224-3p	miR-374b-5p	miR-620
	miR-1197	miR-380-3p	miR-1224-5p	miR-376c-5p	miR-626
	miR-122-5p	miR-382-3p	miR-1236-3p	miR-378d	miR-628-3p
	miR-1245b-5p	miR-411-5p	miR-1245a	miR-378g	miR-631
	miR-1246	miR-421	miR-1249-3p	miR-381-5p	miR-641
	miR-1253	miR-432-5p	miR-125a-3p	miR-3934-5p	miR-642a-5p
	miR-1255a	miR-433-3p	miR-1261	miR-409-3p	miR-644a
	miR-1257	miR-4454+miR-7975	miR-1266-5p	miR-423-3p	miR-650
	miR-1258	miR-4455	miR-1269a	miR-423-5p	miR-6503-3p
	miR-126-3p	miR-448	miR-1285-3p	miR-4286	miR-6503-5p
	miR-1268b	miR-450a-2-3p	miR-1287-5p	miR-433-5p	miR-654-3p
	miR-127-3p	miR-451a	miR-129-2-3p	miR-4421	miR-655-3p
	miR-1270	miR-4707-5p	miR-1298-5p	miR-4451	miR-660-3p
	miR-1271-5p	miR-487a-3p	miR-1299	miR-449b-5p	miR-661
	miR-128-1-5p	miR-487b-3p	miR-1304-3p	miR-450a-1-3p	miR-664a-3p
	miR-1285-5p	miR-495-3p	miR-1306-3p	miR-450a-5p	miR-665
	miR-1290	miR-498	miR-1307-3p	miR-450b-3p	miR-6721-5p
	miR-1295a	miR-499a-5p	miR-133a-3p	miR-4536-3p	miR-675-5p
			miR-140-3p	miR-4536-5p	

	miR-1296-3p<br>miR-1297<br>miR-1305<br>miR-1307-5p<br>miR-130a-3p<br>miR-132-3p<br>miR-1323<br>miR-133a-5p<br>miR-134-3p<br>miR-138-5p<br>miR-142-3p<br>miR-143-3p<br>miR-144-3p<br>miR-147a<br>miR-148b-3p<br>miR-149-5p<br>miR-150-5p<br>miR-155-5p<br>miR-15a-5p<br>miR-15b-5p<br>miR-16-5p<br>miR-1827<br>miR-183-5p<br>miR-186-5p<br>miR-188-5p<br>miR-191-5p<br>miR-196a-5p<br>miR-197-3p<br>miR-199a-3p+miR-199b-3p<br>miR-199a-5p<br>miR-200b-3p<br>miR-501-3p<br>miR-503-5p<br>miR-508-3p<br>miR-513b-5p<br>miR-514a-3p<br>miR-514b-5p<br>miR-516b-5p<br>miR-517a-3p<br>miR-518f-3p<br>miR-519c-3p<br>miR-520h<br>miR-525-5p<br>miR-526a+518c-5p+518d-5p<br>miR-542-3p<br>miR-543<br>miR-548ah-5p<br>miR-548ar-3p<br>miR-548ar-5p<br>miR-548e-5p<br>miR-548g-3p<br>miR-548v<br>miR-548y<br>miR-548z+miR-548h-3p<br>miR-549a<br>miR-551b-3p<br>miR-574-5p<br>miR-575<br>miR-584-3p<br>miR-585-3p<br>miR-587	miR-140-5p<br>miR-146b-5p<br>miR-181a-3p<br>miR-181a-5p<br>miR-181b-2-3p<br>miR-182-3p<br>miR-185-5p<br>miR-18a-5p<br>miR-1909-3p<br>miR-190a-3p<br>miR-190a-5p<br>miR-1910-5p<br>miR-192-5p<br>miR-193b-3p<br>miR-194-5p<br>miR-197-5p<br>miR-1973<br>miR-200c-3p<br>miR-203a-5p<br>miR-205-5p<br>miR-206<br>miR-211-3p<br>miR-211-5p<br>miR-212-3p<br>miR-214-3p<br>miR-216a-5p<br>miR-216b-5p<br>miR-219a-2-3p<br>miR-219a-5p<br>miR-22-3p<br>miR-221-3p<br>miR-224-5p<br>miR-454-3p<br>miR-4647<br>miR-4741<br>miR-4755-5p<br>miR-4787-5p<br>miR-4792<br>miR-483-5p<br>miR-485-5p<br>miR-486-3p<br>miR-489-3p<br>miR-492<br>miR-497-5p<br>miR-502-3p<br>miR-504-5p<br>miR-505-3p<br>miR-506-3p<br>miR-506-5p<br>miR-507<br>miR-509-3-5p<br>miR-509-3p<br>miR-509-5p<br>miR-510-5p<br>miR-512-5p<br>miR-513a-5p<br>miR-513c-3p<br>miR-514b-3p<br>miR-515-3p<br>miR-515-5p<br>miR-518b<br>miR-518c-3p<br>miR-5196-3p+miR-6732-3p<br>miR-758-3p+miR-411-3p<br>miR-769-3p<br>miR-770-5p<br>miR-873-3p<br>miR-873-5p<br>miR-874-3p<br>miR-876-5p<br>miR-877-5p<br>miR-885-3p<br>miR-890<br>miR-892a<br>miR-9-5p<br>miR-922<br>miR-92b-3p<br>miR-93-5p<br>miR-96-5p<br>miR-98-3p<br>miR-98-5p<br>miR-99b-5p

	miR-2053<br>miR-208b-3p<br>miR-20a-5p+miR-20b-5p<br>miR-21-5p<br>miR-215-5p<br>miR-219b-3p<br>miR-223-3p<br>miR-23a-3p<br>miR-25-3p<br>miR-25-5p<br>miR-26a-5p<br>miR-26b-5p<br>miR-27a-3p<br>miR-28-5p<br>miR-299-5p<br>miR-29a-3p<br>miR-300<br>miR-301a-5p<br>miR-301b-3p<br>miR-302b-3p<br>miR-302d-3p<br>miR-30a-3p<br>miR-30e-5p<br>miR-3127-5p<br>miR-3131<br>miR-3144-3p<br>miR-3147<br>miR-3151-5p<br>miR-324-3p<br>miR-331-3p<br>miR-346	miR-593-3p<br>miR-597-5p<br>miR-607<br>miR-608<br>miR-612<br>miR-615-3p<br>miR-627-3p<br>miR-627-5p<br>miR-630<br>miR-639<br>miR-640<br>miR-643<br>miR-648<br>miR-651-5p<br>miR-654-5p<br>miR-656-3p<br>miR-663a<br>miR-664b-3p<br>miR-7-5p<br>miR-744-5p<br>miR-761<br>miR-764<br>miR-765<br>miR-766-3p<br>miR-767-5p<br>miR-802<br>miR-887-5p<br>miR-888-5p<br>miR-889-3p<br>miR-936<br>miR-939-5p<br>miR-95-3p	miR-23b-3p<br>miR-28-3p<br>miR-297<br>miR-29c-3p<br>miR-301b-5p<br>miR-302a-3p<br>miR-302c-3p<br>miR-3065-3p<br>miR-3065-5p<br>miR-30b-5p<br>miR-30c-5p<br>miR-30d-5p<br>miR-3130-3p<br>miR-3136-5p<br>miR-3144-5p<br>miR-3158-3p<br>miR-3182<br>miR-3195<br>miR-32-5p<br>miR-3202<br>miR-320a<br>miR-320e<br>miR-323b-3p<br>miR-323b-5p<br>miR-325<br>miR-329-3p<br>miR-329-5p<br>miR-330-5p<br>miR-335-5p<br>miR-337-5p<br>miR-339-5p<br>miR-33a-5p	miR-519b-5p+miR-519c-5p+miR-523-5p+miR-518e-5p+miR-522-5p+miR-519a-5p<br>miR-520d-5p+miR-527+miR-518a-5p<br>miR-521<br>miR-522-3p<br>miR-523-3p<br>miR-526b-5p<br>miR-532-3p<br>miR-548a-3p<br>miR-548a-5p<br>miR-548ad-3p<br>miR-548h-5p<br>miR-548j-3p<br>miR-548n<br>miR-548q<br>miR-551a<br>miR-552-3p<br>miR-553<br>miR-555<br>miR-556-3p<br>miR-556-5p<br>miR-561-3p<br>miR-562<br>miR-564<br>miR-566<br>miR-567<br>miR-568<br>miR-570-3p

	miR-34a-5p		miR-33b-5p	miR-576-5p	

Supplementary Table 8b: VENN diagram from Figure 7D in alphabetic order

Plasma UICC low		Overlap plasma UICC low and high			Plasma UICC high
let-7c-5p	miR-382-3p	let-7a-5p	miR-23a-3p	miR-525-5p	miR-1234-3p
miR-106a-5p+miR-	miR-3934-5p	let-7b-5p	miR-25-3p	miR-526a+518c-	miR-125b-5p
17-5p	miR-423-3p	let-7d-5p	miR-25-5p	5p+518d-5p	miR-1272
miR-106b-5p	miR-423-5p	let-7f-5p	miR-26a-5p	miR-532-3p	miR-221-3p
miR-1183	miR-432-5p	let-7g-5p	miR-26b-5p	miR-542-3p	miR-328-5p
miR-1185-2-3p	miR-433-5p	miR-1-5p	miR-27a-3p	miR-543	miR-4516
miR-1193	miR-4421	miR-100-5p	miR-28-5p	miR-548ad-3p	miR-520c-3p
miR-1200	miR-449b-5p	miR-103a-3p	miR-299-5p	miR-548ah-5p	miR-548n
miR-1206	miR-450a-1-3p	miR-107	miR-29a-3p	miR-548ar-3p	miR-579-5p
miR-1249-3p	miR-450a-5p	miR-10b-5p	miR-300	miR-548ar-5p	
miR-1255b-5p	miR-450b-3p	miR-1178-3p	miR-301a-5p	miR-548e-5p	
miR-125a-3p	miR-4536-3p	miR-1185-1-3p	miR-301b-3p	miR-548g-3p	
miR-1261	miR-454-3p	miR-1197	miR-302b-3p	miR-548v	
miR-1266-5p	miR-4755-5p	miR-122-5p	miR-302d-3p	miR-548y	
miR-1279	miR-4787-5p	miR-1245b-5p	miR-30a-3p	miR-548z+miR-548h-	
miR-128-1-5p	miR-4792	miR-1246	miR-30d-5p	3p	
miR-1286	miR-486-3p	miR-1253	miR-30e-5p	miR-549a	
miR-1298-5p	miR-487b-3p	miR-1255a	miR-3127-5p	miR-551b-3p	
miR-1299	miR-492	miR-1257	miR-3130-3p	miR-574-5p	
miR-1307-3p	miR-497-5p	miR-1258	miR-3131	miR-575	
miR-133a-3p	miR-502-3p	miR-126-3p	miR-3144-3p	miR-584-3p	
miR-140-3p	miR-502-5p	miR-1268b	miR-3147	miR-585-3p	
miR-140-5p	miR-503-5p	miR-1269a	miR-3151-5p	miR-587	
miR-146a-5p	miR-504-5p	miR-127-3p	miR-320e	miR-593-3p	
miR-146b-5p	miR-505-3p	miR-1270	miR-324-3p	miR-597-5p	
miR-148a-3p	miR-506-3p	miR-1271-5p	miR-331-3p	miR-598-3p	
miR-149-5p	miR-507	miR-1285-5p	miR-337-5p	miR-605-5p	

miR-151b<br>miR-181a-3p<br>miR-181a-5p<br>miR-181b-2-3p<br>miR-184<br>miR-185-5p<br>miR-18a-5p<br>miR-190a-3p<br>miR-192-5p<br>miR-197-5p<br>miR-1973<br>miR-200c-3p<br>miR-203a-5p<br>miR-205-5p<br>miR-2113<br>miR-212-3p<br>miR-216a-5p<br>miR-216b-5p<br>miR-219a-2-3p<br>miR-222-3p<br>miR-28-3p<br>miR-301b-5p<br>miR-302a-3p<br>miR-3065-3p<br>miR-30b-5p<br>miR-30c-5p<br>miR-30e-3p<br>miR-3136-5p<br>miR-3195<br>miR-32-5p<br>miR-323b-5p<br>miR-329-5p<br>miR-509-5p<br>miR-510-5p<br>miR-512-5p<br>miR-522-3p<br>miR-523-3p<br>miR-526b-5p<br>miR-548a-3p<br>miR-548a-5p<br>miR-548h-5p<br>miR-548q<br>miR-551a<br>miR-552-3p<br>miR-556-3p<br>miR-556-5p<br>miR-564<br>miR-566<br>miR-568<br>miR-577<br>miR-582-3p<br>miR-582-5p<br>miR-584-5p<br>miR-590-5p<br>miR-620<br>miR-626<br>miR-642a-5p<br>miR-644a<br>miR-650<br>miR-6503-5p<br>miR-654-3p<br>miR-654-5p<br>miR-655-3p<br>miR-660-3p	miR-129-2-3p<br>miR-1290<br>miR-1295a<br>miR-1296-3p<br>miR-1297<br>miR-1305<br>miR-1307-5p<br>miR-130a-3p<br>miR-132-3p<br>miR-1323<br>miR-133a-5p<br>miR-134-3p<br>miR-138-5p<br>miR-142-3p<br>miR-143-3p<br>miR-144-3p<br>miR-147a<br>miR-148b-3p<br>miR-150-5p<br>miR-155-5p<br>miR-15a-5p<br>miR-15b-5p<br>miR-16-5p<br>miR-183-5p<br>miR-186-5p<br>miR-188-5p<br>miR-191-5p<br>miR-196a-5p<br>miR-197-3p<br>miR-1972<br>miR-199a-3p+miR-199b-3p<br>miR-33a-5p<br>miR-346<br>miR-34a-5p<br>miR-365a-3p+miR-365b-3p<br>miR-370-3p<br>miR-371a-5p<br>miR-374a-5p<br>miR-376a-3p<br>miR-377-3p<br>miR-378e<br>miR-378f<br>miR-378g<br>miR-378h<br>miR-378i<br>miR-380-3p<br>miR-411-5p<br>miR-421<br>miR-4286<br>miR-433-3p<br>miR-4454+miR-7975<br>miR-4455<br>miR-448<br>miR-450a-2-3p<br>miR-451a<br>miR-4647<br>miR-4707-5p<br>miR-487a-3p<br>miR-495-3p<br>miR-496<br>miR-498<br>miR-499a-5p<br>miR-607<br>miR-608<br>miR-612<br>miR-615-3p<br>miR-627-3p<br>miR-627-5p<br>miR-630<br>miR-639<br>miR-640<br>miR-643<br>miR-648<br>miR-651-5p<br>miR-656-3p<br>miR-663a<br>miR-664b-3p<br>miR-7-5p<br>miR-744-5p<br>miR-761<br>miR-764<br>miR-765<br>miR-766-3p<br>miR-767-5p<br>miR-802<br>miR-887-5p<br>miR-888-5p<br>miR-889-3p<br>miR-936<br>miR-939-5p<br>miR-95-3p	

miR-335-5p<br>miR-33b-5p<br>miR-340-5p<br>miR-3605-5p<br>miR-3613-3p<br>miR-3614-5p<br>miR-363-3p<br>miR-367-3p<br>miR-369-3p<br>miR-374a-3p<br>miR-376c-5p<br>miR-378d<br>miR-379-5p<br>miR-664a-3p<br>miR-6721-5p<br>miR-760<br>miR-770-5p<br>miR-873-3p<br>miR-873-5p<br>miR-876-5p<br>miR-877-5p<br>miR-892a<br>miR-93-5p<br>miR-937-3p<br>miR-99b-5p	miR-199a-5p<br>miR-200b-3p<br>miR-2053<br>miR-208b-3p<br>miR-20a-5p+miR-20b-5p<br>miR-21-5p<br>miR-211-3p<br>miR-211-5p<br>miR-2110<br>miR-215-5p<br>miR-219b-3p<br>miR-223-3p<br>miR-501-3p<br>miR-506-5p<br>miR-508-3p<br>miR-513b-5p<br>miR-514a-3p<br>miR-514b-5p<br>miR-516b-5p<br>miR-517a-3p<br>miR-517c-3p+miR-519a-3p<br>miR-518f-3p<br>miR-519c-3p<br>miR-520h	

Supplementary Table 9a: VENN diagram from Figure 7G in alphabetic order

Saliva HPV negative	Overlap saliva HPV negative and positive		Saliva HPV positive		
miR-148b-3p	let-7b-5p	miR-346	let-7a-5p	miR-365a-3p+miR-	miR-664b-3p
miR-29a-3p	let-7f-5p	miR-34a-5p	miR-10b-5p	365b-3p	miR-764
miR-377-3p	miR-1246	miR-376a-3p	miR-1197	miR-371a-5p	miR-873-5p
miR-378i	miR-1253	miR-378e	miR-1245b-5p	miR-380-3p	miR-936
miR-4286	miR-1255a	miR-378f	miR-1257	miR-382-3p	miR-939-5p
miR-4455	miR-1258	miR-378h	miR-125b-5p	miR-432-5p	miR-95-3p
miR-501-3p	miR-1283	miR-379-5p	miR-1268b	miR-450a-2-3p	
miR-548g-3p	miR-1285-5p	miR-411-5p	miR-127-3p	miR-4516	
miR-607	miR-1290	miR-421	miR-1271-5p	miR-4755-5p	
miR-612	miR-1296-3p	miR-4454+miR-7975	miR-1272	miR-499a-5p	
miR-640	miR-1297	miR-448	miR-128-1-5p	miR-502-3p	
	miR-1305	miR-495-3p	miR-1295a	miR-506-5p	
	miR-1323	miR-496	miR-134-3p	miR-508-3p	
	miR-133a-5p	miR-514a-3p	miR-149-5p	miR-516b-5p	
	miR-142-3p	miR-514b-5p	miR-186-5p	miR-517a-3p	
	miR-155-5p	miR-518f-3p	miR-200b-3p	miR-526a+518c-	
	miR-183-5p	miR-520h	miR-203a-3p	5p+518d-5p	
	miR-188-5p	miR-548ah-5p	miR-219b-3p	miR-542-3p	
	miR-199a-3p+miR-	miR-548ar-5p	miR-26a-5p	miR-548ad-3p	
	199b-3p	miR-548e-5p	miR-28-3p	miR-548ar-3p	
	miR-205-5p	miR-574-5p	miR-28-5p	miR-548v	
	miR-2053	miR-584-3p	miR-299-5p	miR-548y	
	miR-208b-3p	miR-585-3p	miR-301b-3p	miR-549a	
	miR-21-5p	miR-593-3p	miR-302a-3p	miR-575	
	miR-223-3p	miR-597-5p	miR-302b-3p	miR-608	
	miR-23a-3p	miR-615-3p	miR-3130-3p	miR-627-3p	
	miR-301a-5p	miR-644a	miR-323b-3p	miR-627-5p	
	miR-302d-3p	miR-744-5p	miR-324-3p	miR-643	
	miR-3144-3p		miR-340-5p	miR-656-3p	
	miR-3147				

Supplementary Table 9b: VENN diagram from Figure 7J in alphabetic order

Saliva UICC low	Overlap saliva UICC low and high			Saliva UICC high	
miR-1272	let-7b-5p	miR-301a-5p	miR-593-3p	let-7a-5p	miR-382-3p
miR-4516	let-7f-5p	miR-301b-3p	miR-597-5p	miR-10b-5p	miR-4286
	miR-1197	miR-302d-3p	miR-607	miR-1185-1-3p	miR-432-5p
	miR-1246	miR-3144-3p	miR-612	miR-1185-2-3p	miR-4455
	miR-1253	miR-346	miR-615-3p	miR-1245b-5p	miR-448
	miR-1255a	miR-34a-5p	miR-627-5p	miR-1257	miR-4707-5p
	miR-1258	miR-376a-3p	miR-644a	miR-1269a	miR-4755-5p
	miR-125b-5p	miR-378e	miR-744-5p	miR-127-3p	miR-501-3p
	miR-1268b	miR-378f		miR-1270	miR-506-5p
	miR-1283	miR-378h		miR-1271-5p	miR-508-3p
	miR-1285-5p	miR-378i		miR-1297	miR-511-5p
	miR-1290	miR-379-5p		miR-148b-3p	miR-514b-3p
	miR-1295a	miR-411-5p		miR-196a-5p	miR-517a-3p
	miR-1296-3p	miR-421		miR-197-3p	miR-519c-3p
	miR-1305	miR-4454+miR-7975		miR-1972	miR-525-5p
	miR-1323	miR-450a-2-3p		miR-200b-3p	miR-526a+518c-
	miR-133a-5p	miR-495-3p		miR-219b-3p	5p+518d-5p
	miR-134-3p	miR-496		miR-224-5p	miR-542-3p
	miR-142-3p	miR-499a-5p		miR-25-5p	miR-548g-3p
	miR-155-5p	miR-514a-3p		miR-29a-3p	miR-548v
	miR-183-5p	miR-514b-5p		miR-300	miR-549a
	miR-186-5p	miR-516b-5p		miR-302a-3p	miR-605-5p
	miR-188-5p	miR-518f-3p		miR-30c-5p	miR-627-3p
	miR-199a-3p+miR-	miR-520h		miR-30e-5p	miR-640
	199b-3p	miR-548ad-3p		miR-3127-5p	miR-643
	miR-203a-3p	miR-548ah-5p		miR-3130-3p	miR-656-3p
	miR-205-5p	miR-548ar-3p		miR-3147	miR-7-5p
	miR-2053	miR-548ar-5p		miR-324-3p	miR-761

	miR-208b-3p<br>miR-21-5p<br>miR-223-3p<br>miR-23a-3p<br>miR-26a-5p<br>miR-299-5p<br>miR-548e-5p<br>miR-548y<br>miR-574-5p<br>miR-575<br>miR-584-3p<br>miR-585-3p	miR-363-5p<br>miR-365a-3p+miR-365b-3p<br>miR-371a-5p<br>miR-377-3p<br>miR-378g<br>miR-764<br>miR-765<br>miR-766-3p<br>miR-873-3p<br>miR-937-3p<br>miR-939-5p

Supplementary Table 10: Significantly differentially present miRNAs between patients with HPV^{-} and HPV^{+} disease or low and high UICC stage

Exosomal miRNA	Biofluid	Clinical data	Expression ratio*	p-value
miR-138-5p	Plasma	HPV	1.577	0.024
miR-25-5p			1.603	0.024
miR-663a			1.592	0.024
let-7a-5p			1.503	0.036
miR-191-5p			1.633	0.038
miR-223-3p	Plasma	UICC	0.499	0.008
miR-451a			0.514	0.017
miR-142-3p			0.745	0.032
miR-205-5p	Saliva	HPV	1.749	0.016
miR-4454+miR-7975			3.631	0.019
miR-183-5p			1.601	0.038
hsa-miR-3144-3p	Saliva	UICC	1.486	0.009
hsa-miR-411-5p			1.285	0.030
hsa-miR-744-5p			1.606	0.030

* Expression ratio refers to HPV^{+} / HPV^{-} or UICC high / UICC low
